# Effects of incentive spirometer training on dyspnea and functional status in patients with long COVID

**DOI:** 10.1371/journal.pone.0351553

**Published:** 2026-06-22

**Authors:** Yao-Hsiang Chen, Chia-Huei Lin, Ju-Han Liu, Hsin-An Lin, Yu-Shan Hsieh

**Affiliations:** 1 School of Nursing, National Taipei University of Nursing and Health Sciences, Taipei City, Taiwan; 2 Department of Nursing, Tri-Service General Hospital Songshan Branch, Taipei City, Taiwan; 3 School of Nursing, National Defense Medical University, Taipei City, Taiwan; 4 Graduate Institute of Medical Sciences, National Defense Medical Center, Taipei City, Taiwan; 5 Institute of Traditional Medicine, School of Medicine, National Yang Ming Chiao Tung University, Taipei City, Taiwan; 6 Division of Infection, Department of Medicine, Tri-Service General Hospital Songshan, Branch, National Defense Medical Center, Taipei City, Taiwan; 7 Performance Examination center, National Taipei University of Nursing and Health Sciences, Taipei City, Taiwan; Srebrnjak Children’s Hospital, CROATIA

## Abstract

**Background:**

Since the emergence of Coronavirus Disease 2019 (COVID-19), it has become a global pandemic, profoundly affecting public health and daily life. Many recovering individuals report persistent or recurrent symptoms—fatigue, palpitations, cognitive impairment, shortness of breath, anxiety, and chest discomfort. These lingering effects impair work, daily function, and social interaction, placing a significant burden on individual quality of life and society.

**Objective:**

This study aims to evaluate the effectiveness of using an induced Incentive Spirometer as a respiratory training tool to relieve long COVID symptoms.

**Methods:**

This study, conducted from July 1, 2023, to May 11, 2024, at a regional teaching hospital in northern Taiwan, involved participants who had recovered from COVID-19 within the past year and had at least one long COVID respiratory symptom. Participants were assigned to one waiting control group and four experimental groups based on recovery time: within 3 months (Experimental Group 1), 3–6 months (Experimental Group 2), 6–9 months (Experimental Group 3), and 9–12 months (Experimental Group 4). The waiting control group received no interventions, while the experimental groups underwent inspiratory training using an induced Incentive Spirometer three times a week for 6 weeks (30 repetitions per session). Assessments were conducted before and after the intervention. Primary outcomes were the Dyspnoea-12 scale and Post-COVID-19 Functional Status scale. Secondary outcomes included the 6-minute walk distance and CaO₂.

**Results:**

Ninety participants were enrolled, with five withdrawing, leaving 85 for final analysis. After 6 weeks of intervention, the waiting control group showed no significant changes in dyspnea (*p* = 0.463) or post-COVID-19 functional status (*p* = 0.343). In contrast, all experimental groups showed significant improvements. Dyspnoea-12 scale scores improved in Experimental Groups 1 (*p* < 0.001), 2 (*p* = 0.008), 3 (*p* = 0.011), and 4 (*p* = 0.001). The Post-COVID-19 Functional Status scale also showed improvements in all Experimental Groups (Group 1: *p* < 0.001, Group 2: *p* = 0.003, Group 3: *p* = 0.002, and Group 4: *p* = 0.011). Significant improvements in 6-min walk distance were observed in some experimental groups, improvements were seen in Experimental Groups 1 (*p* < 0.001), 2 (*p* = 0.027), 3 (*p* = 0.68), and 4 (*p* = 0.172). No significant changes in CaO2 were observed (pre-test, *p* = 0.872 and post-test, *p* = 0.585).

**Conclusion:**

Respiratory training using an induced Incentive Spirometer may help alleviate dyspnea and improve post-COVID-19 functional status in individuals with Long COVID. Earlier intervention appeared to yield better outcomes, although improvements were also observed even 9–12 months after infection. However, further studies with comprehensive pulmonary assessments are needed to confirm these findings.

**Clinical trial number:**

NCT06165835, registered on 9 December 2023.

## Introduction

The Coronavirus Disease 2019 (COVID-19) has emerged as one of the most significant global public health crises of the 21st century. While acute-phase mortality has declined substantially—owing to viral evolution, medical advances, and widespread vaccination—many patients continue to experience persistent symptoms following infection. This condition, referred to as “Long COVID,” is defined as the persistence or onset of new symptoms 3 months after a SARS-CoV-2 infection, lasting for at least 2 months without an alternative diagnosis [1]. Long COVID affects approximately 80% of patients and commonly presents with fatigue, dyspnea, chest tightness, and cognitive or emotional disturbances, all of which significantly reduce the quality of life [2–7]. Currently, there is a lack of definitive treatment for Long COVID. Several systematic reviews and meta-analyses have investigated the effects of pulmonary rehabilitation in patients with long COVID, consistently suggesting improvements in post-COVID-19 symptoms [8–10]. However, the current evidence remains limited and heterogeneous. [11–14], and no standardized or definitive therapeutic approach has been established. Therefore, evaluating the effectiveness of respiratory training for Long COVID has emerged as an urgent public health priority in the post-pandemic era.

Although aerobic exercise is known to improve cardiopulmonary function, it often poses challenges for individuals with impaired lung function, potentially leading to hypoxemia or hypercapnia [15]. In contrast, pulmonary rehabilitation exercises present a lower risk and are easier to implement, making them a more viable alternative for patients experiencing dyspnea following COVID-19 recovery [16]. An incentive spirometer (IS) is a simple and safe handheld device that provides real-time visual feedback on inspiratory strength and volume, thereby promoting lung expansion and enhancing lung capacity [17–19]. Previous studies have shown that IS can improve maximum inspiratory volume and oxygenation levels and reduce anxiety in patients with COVID-19 [20–22]. However, most available evidence pertains primarily to acute or postoperative populations [23,24], with limited systematic investigation into the effects of IS on Long COVID symptoms.

Although evidence-based medicine supports the effectiveness of IS in improving pulmonary function, its role in managing Long COVID symptoms—especially concerning the optimal timing of intervention during recovery—remains unclear. Therefore, an important aim of the present study is to determine whether, although IS cannot replace pulmonary rehabilitation, it may serve as a low-cost, self-administered intervention option, and to evaluate the efficacy of IS as a respiratory training intervention for improving dyspnea and functional status in post-COVID-19 patients. Furthermore, it seeks to examine the differential outcomes based on varying recovery intervals, with the objective of developing a more specific, feasible, and evidence-based clinical rehabilitation strategy to address existing gaps in the study.

## Methods

Overall, 90 eligible participants were enrolled in this study at a regional teaching hospital in Taipei, Taiwan, from July 1, 2023, to May 11, 2024. Participants were randomly assigned to the experimental and control groups. The experimental group was further stratified based on recovery duration. This study was approved by the Institutional Review Board of the Tri-Service General Hospital (Approval No.: A202305044) and all participants provided written informed consent after completing a formal consent process.

### Study design

**Fig 1:**
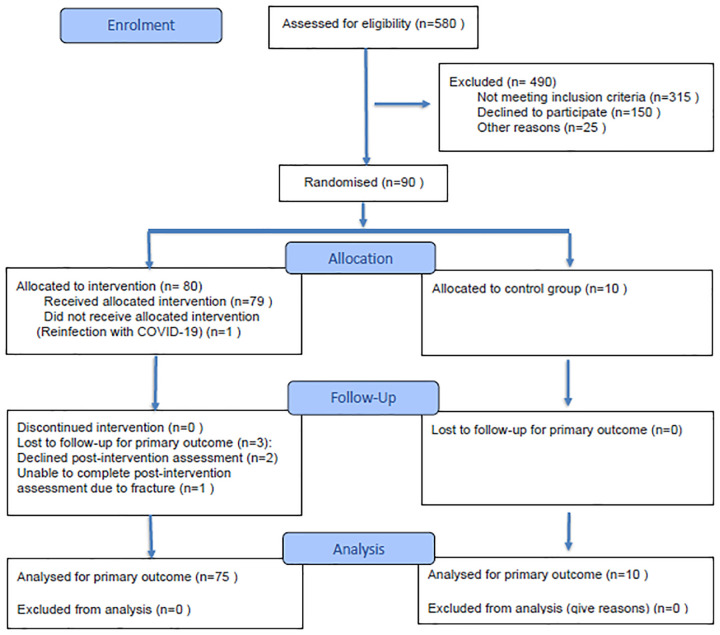
Flow diagram of participant enrollment and allocation.

The flow diagram of participant enrollment and allocation is shown in Fig 1. Due to the natural tendency for Long COVID symptoms to improve over time, strict randomization could lead to imbalance in disease severity at the time of intervention and introduce systematic bias resulting from spontaneous recovery. Moreover, it is not feasible in clinical practice to require patients who actively seek intervention to be assigned to a control group. To ensure ethical considerations for the control group, participants in the control group were offered the intervention after the study period if they wished; however, data collected after the intervention were not included in the analysis(Fig 1).

**Fig 2:**
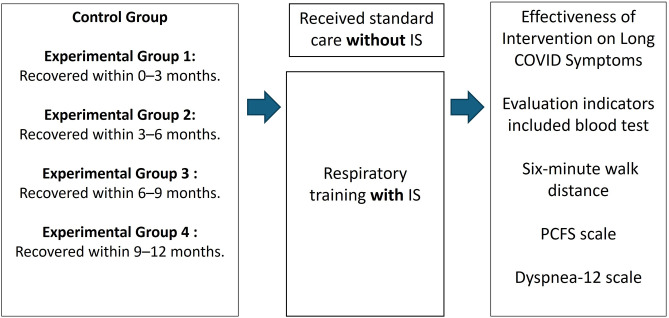
Study framework.

Accordingly, the study adopted an open-label randomized controlled trial design. Eligible participants were randomly assigned to the experimental group (EG) or waitlist control group (CG) using a computer-generated random number. Ten participants were randomly assigned to the CG, while the other participants were placed in the EG, which was further stratified into four subgroups based on the time elapsed since COVID-19 recovery. Only participants in the EG received a 6-week IS intervention, whereas those in the CG continued with routine care and received no additional pulmonary rehabilitation. Participants who dropped out were excluded from the final analysis (Fig 2).

Participants in the EG received inspiratory training using an IS (Taiwan FDA Device Approval No. 003427). They were instructed to complete three training sessions per week, each consisting of 30 sustained inhalations lasting at least 3 s. Training intensity was adjusted based on individual tolerance and physical condition. To support adherence and evaluate the feasibility of the intervention, the research team conducted regular telephone follow-ups at the third weeks. All participants completed pre- and post-intervention assessments over 6 weeks. Primary outcome measures included the D-12 scale, the PCFs scale and blood biomarkers (CaO₂). The secondary outcome is 6-min walk distance (6MWD). These assessments were conducted to evaluate the effects of the IS intervention on dyspnea and physical function among post-COVID-19 individuals

### Participants and group allocation criteria

Individuals who had recovered from COVID-19 within the past year but continued to experience at least one respiratory-related symptom associated with Long COVID were recruited in this study. Participants were assigned into five groups: one waiting control group and four experimental groups, defined a priori based on the study objective of comparing intervention effects across different recovery durations and following the commonly adopted stratification of Long COVID symptom persistence (within 3, 3–6, 6–9, and 9–12 months post-recovery).

**Fig 3:**
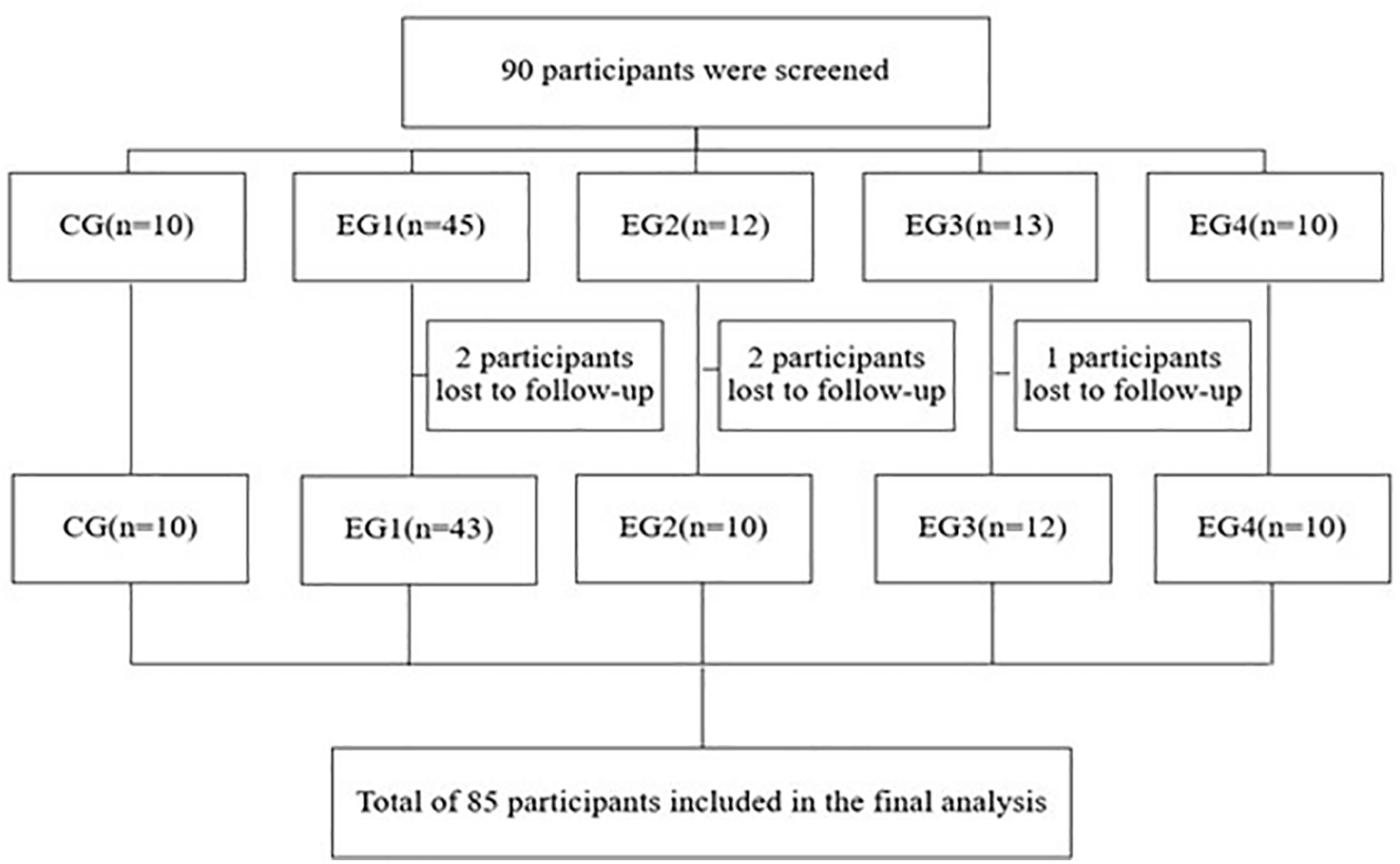
Participant recruitment flowchart.

Ten participants were randomly assigned to the CG, while the other participants were placed in the EG, which was further stratified into four subgroups based on the time elapsed since COVID-19 recovery. (Fig 3).

***Inclusion criteria:***

1. Individuals who had recovered from COVID-19 within the past year (confirmed by a negative rapid antigen test). The corresponding ICD-10 codes included U07.1 (COVID-19; virus identified) and U09.0 (Post COVID-19 condition; unspecified).
2. Presence of at least one respiratory-related symptom related to Long COVID (such as exertional dyspnea, shortness of breath, chest tightness, cough, or dyspnea).
3. Aged between 20 and 90 years.
4. Intact consciousness with normal cognitive function and behavioral stability.
5. Ability to communicate, verbally or non-verbally, and understand Mandarin Chinese or a Taiwanese dialect.
6. Willingness to participate in the study and accept random group assignments.

***Exclusion criteria:***

1. Individuals with severe disabilities or those who are permanently bedridden.
2. Diagnosed with dementia (such as Alzheimer’s disease and Parkinson’s disease)
3. Presence of acute psychiatric symptoms that impair communication.
4. Individuals with a high risk of litigation.
5. Diagnosed with chronic obstructive pulmonary disease (COPD) or any other respiratory disorders.
6. Diagnosed with moderate to severe heart disease.

### Quality control

The initial IS training session was conducted under the supervision of a nurse, who provided instructions and guidance to ensure participants fully understood the standardized training protocol. Written informed consent was obtained from all participants before study enrollment.

### Statistical analysis and sample size calculation

The required sample size was calculated using G*Power software (version 3.1.2, Germany), employing repeated measures of one-way analysis of variance (ANOVA) (within-between interaction) as the analysis method. The following parameters were used: statistical power of 0.95, effect size of 0.25, and significance level (α) of 0.05. With five groups and two measurement points, the estimated minimum sample size was 80. Considering a potential dropout rate of approximately 10%, the final target sample size was adjusted to 90 participants.

All data were organized using Excel 2019 (Microsoft, USA) and analyzed with SPSS version 18.0 (SPSS Inc., Chicago, IL). Descriptive statistics were reported as means, standard deviations, and percentages. Inferential analyses included independent and paired t-tests, ANOVA, chi-square tests, and both linear and logistic regression. Generalized Estimating Equations (GEEs) were used to evaluate group-by-time interaction effects and assess the efficacy of the intervention. A p-value of < 0.05 was considered statistically significant.

### Research instruments and measurements

Based on the study objectives, the research instruments were categorized as follows:

1 Demographic and Clinical Characteristics

The collected variables included age, sex, medical history, oxygen requirement, frequency of regular physical activity, number of previous COVID-19 infections, inspiratory volume (measured using an incentive spirometer), and medication use during the infection period.

2 Chinese Version of the Post-COVID-19 Functional Status Scale (PCFS Scale).

Klok et al. developed the Post-COVID-19 Functional Status Scale to assess the functional status of participants following COVID-19 recovery. The scale includes five levels, ranging from 0 (no functional limitations) to 4 (severe limitations), with level 5 indicating death. The scale has been validated and shown to effectively reflect the influence of Long COVID symptoms on daily functioning [25–27].

3 Dyspnoea-12 scale (D-12)

The Dyspnoea-12 scale was developed by Yorke et al. It was used to assess the subjective experience of breathlessness of the participants, encompassing physical and emotional dimensions across 12 items. Higher scores indicate more severe dyspnea. The Chinese version has demonstrated strong reliability and validity [28–30].

4 Blood Biomarkers

Hemoglobin (Hb) and hematocrit (Hct) levels were measured using the HemoSmart GOLD hemoglobin analyzer (Taiwan FDA Registration No. 004460/004455). Peripheral oxygen saturation (SpO₂), measured with a fingertip pulse oximeter, was used as a surrogate for arterial oxygen saturation (SaO₂), supported by prior validation studies [31–33]. Arterial oxygen content (CaO₂) was calculated using the formula: CaO₂ = Hb × SaO₂ × 1.34/ 100 (mL/dL). This calculation served as a reference for assessing pulmonary oxygenation function [34].

5 Six-Minute Walk Test

The 6MWT was used to assess the exercise tolerance and cardiopulmonary function of the participants. The main outcome measure was the 6-min walk distance (6MWD). Heart rate and SpO₂ were recorded before and after the test to assess physiological responses [35–37].

## Results

### Participant demographics and baseline characteristics

Overall, 90 eligible participants were enrolled in this study at a regional teaching hospital in Taipei, Taiwan, from July 1, 2023, to May 11, 2024. Participants were randomly assigned to the experimental and control groups. The experimental group was further stratified based on recovery duration. Five participants (5.6%) were lost to follow-up owing to reinfection, fractures, or voluntary withdrawal. Among the remaining 85 participants, 3 participants completed only the questionnaire assessments but did not undergo the physiological measurements. Therefore, their physiological data were excluded from the analysis, whereas their questionnaire data were retained for relevant statistical analyses. Ultimately, 85 participants were included in the final analysis. Group allocation was as follows: Control Group (CG): n = 10; Experimental Group 1 (EG1; recovered within 3 months): n = 43; Experimental Group 2 (EG2; recovered 3–6 months): n = 10; Experimental Group 3 (EG3; recovered 6–9 months): n = 12; Experimental Group 4 (EG4; recovered 9–12 months): n = 10. Table 1 provides a summary of the group-specific data (Table 1).

**Table 1:** Respiratory-related long COVID symptoms by group.

	CG	EG1	EG2	EG3	EG4
**Cough**	4(40%)	31(72%)	5(50%)	6(50%)	3(30%)
**Chest tightness**	0(0%)	3(7%)	0(0%)	0(0%)	0(0%)
**Short of breath**	7(70%)	17(40%)	8(80%)	9(75%)	8(80%)
**Excessive sputum**	0(0%)	15(35%)	2(20%)	2(17%)	0(0%)

Of the 85 participants included in the final analysis, 66% were female, with a mean age of 46.3 years. The most prevalent comorbidities were hypertension and cardiovascular disease. Most participants were non-smokers, and approximately 40% reported not engaging in regular physical activity. Regarding their COVID-19 history, 54% had been infected once, while 40% had experienced two infections. Treatments during infection included oral antiviral medications and the traditional Chinese medicine formula NRICM101; a minority of participants received no treatment. Table 2 presents a summary of the detailed demographic and clinical characteristics.

**Table 2:** Baseline demographic and clinical information of participants.

	All	CG	EG1	EG 2	EG 3	EG 4	p value
	(n = 85)	(n = 10)	(n = 43)	(n = 10)	(n = 12)	(n = 10)	
**Gender** [N(%)]							
Male	29(34%)	2(20%)	15(35%)	3(30%)	6(50%)	3(30%)	–
Female	56(66%)	8(80%)	28(65%)	7(70%)	6(50%)	7(70%)	–
**Age [Mean (Median)]**	46.29(45)	38.1(33)	49.4(52)	41.2(40.5)	49.1(53)	43.1(34.5)	0.28
**Height [Mean (SD)]**	164.38(8.25)	161.9(6.05)	163.7(9.05)	165.3(10.25)	167.6(7.1)	164.9(5.17)	0.541
**Weight[Mean (SD)]**	66.96(15.06)	61.6(8.13)	65.4(14.64)	73.9(17.69)	74.3(18.29)	63.9(12.32)	0.123
**Duration Post-Recovery [Mean (SD)]**	4.29(3.88)	6.2(4.5)	1.2(0.63)	5(0.74)	7.5(0.8)	11.1(1.13)	p < 0.001***
**Hypertension** [N(%)]							
Yes	14(16%)	2(20%)	9(21%)	2(20%)	1(8%)	0(0%)	–
No	71(84%)	8(80%)	34(79%)	8(80%)	11(92%)	10(100%)	–
**Cardiovascular Disease** [N(%)]							
Yes	13(15%)	0(0%)	10(23%)	1(10%)	1(8%)	1(10%)	–
No	72(85%)	10(100%)	33(77%)	9(90%)	11(92%)	9(90%)	–
**Diabetes** [N(%)]							
Yes	4(5%)	0(0%)	2(5%)	0(0%)	0(0%)	1(10%)	–
No	81(95%)	10(100%)	41(95%)	10(100%)	12(100%)	9(90%)	–
**Smoking** [N(%)]							
Yes	10(12%)	1(10%)	6(14%)	1(10%)	0(0%)	2(20%)	–
No	74(87%)	9(90%)	36(84%)	9(90%)	12(100%)	8(80%)	–
Quit smoking	1(1%)	0(0%)	1(2%)	0(0%)	0(0%)	0(0%)	–
**Medication** [N(%)]							
No	28(33%)	5(50%)	18(42%)	1(10%)	2(17%)	2(20%)	–
Injectable Antivirals	3(4%)	1(10%)	1(2%)	1(10%)	0(0%)	0(0%)	–
Oral Antivirals	23(27%)	0(0%)	14(33%)	4(40%)	2(17%)	3(30%)	–
NRICM101	23(27%)	4(40%)	6(14%)	3(30%)	7(58%)	3(30%)	–
Other Folk Remedies	7(8%)	0(0%)	3(7%)	1(10%)	1(8%)	2(20%)	–
Injectable Antivirals and NRICM101	1(1%)	0(0%)	1(2%)	0(0%)	0(0%)	0(0%)	–
**Exercise** [N(%)]							
No	36(42%)	2(20%)	19(44%)	5(50%)	5(42%)	5(50%)	–
1 ~ 3 times/week	32(38%)	7(70%)	14(33%)	3(30%)	4(33%)	4(40%)	–
4 ~ 5 times/week	9(11%)	1(10%)	6(14%)	1(10%)	1(8%)	0(0%)	–
6 ~ 7 times/week	8(9%)	0(0%)	4(9%)	1(10%)	2(17%)	1(10%)	–
**Number of Infections** [N(%)]	1.52(0.61)	1.6(0.52)	1.6(0.66)	1.5(0.7)	1.3(0.45)	1.4(0.52)	0.448
1times	46(54%)	4(40%)	21(49%)	6(60%)	9(75%)	6(60%)	–
2 times	34(40%)	6(60%)	18(42%)	3(30%)	3(25%)	4(40%)	–
3 times	5(6%)	0(0%)	4(9%)	1(10%)	0(0%)	0(0%)	–
**Hb (g/dL) [Mean (SD)]**	11.61(1.98)	11.3(1.6)	11.6(2.12)	12.2(1.88)	11.5(1.82)	11.6(2.19)	0.88
**Hct (%) [Mean (SD)]**	34.89(5.86)	33.9(4.78)	34.8(6.25)	36.7(5.63)	34.7(5.42)	34.9(6.58)	0.874
**PCFS [Mean (SD)]**	1.56(0.57)	1.2(0.42)	1.6(0.54)	1.9(0.57)	1.3(0.49)	1.7(0.68)	0.029*
**D-12 [Mean (SD)]**	8.72(7.55)	8.2(7.47)	8.3(7.54)	10.2(9.58)	6(5.43)	12.8(7.13)	0.285
D-12 of physiological items	5.09(3.87)	4.1(3.76)	5.1(4.02)	5.9(4.86)	3.4(2.19)	7.1(3.32)	0.196
D-12 of psychological items	3.62(4.21)	4.1(3.99)	3.2(4.05)	4.3(5.12)	2.6(3.9)	5.7(4.48)	0.403
**pre HR (times/minute) [Mean (SD)]**	84.46(12.82)	88.1(13.26)	83.7(12.59)	85.4(12.69)	77.9(10.26)	91.4(14.17)	0.129
**preSpO**_**2**_ **(%) [Mean (SD)]**	98.68(0.68)	98.7(0.95)	98.7(0.63)	98.6(0.84)	98.8(0.39)	98.4(0.7)	0.63
**6MWD (meter) [Mean (SD)]**	346.7(79.59)	378.1(61.57)	340.1(71.9)	294.5(82.05)	374.1(53.85)	363.1(123.7)	0.088
**post HR (times/minute) [Mean (SD)]**	99.84(16.01)	104.1(25.31)	99.6(14.62)	97.7(15.3)	93.1(7.62)	107(17.46)	0.286
**postSpO**_**2**_ **(%) [Mean (SD)]**	98.35(1.34)	98.6(0.97)	98.6(0.76)	98.3(1.16)	97.5(2.81)	98.2(0.92)	0.159
**CaO2 (ml/dL) [Mean (SD)]**	15.3(2.56)	14.9(2.02)	15.3(2.78)	16.1(2.45)	15(2.37)	15.3(2.86)	0.872
**Ball [Mean (SD)]**	N/A	N/A	2(0.87)	2(0.82)	1.8(0.87)	1.9(1)	0.834

Note 1: Hb = Hemoglobin; Hct = Hematocrit; PCFS = Post-COVID-19 Functional Status total score; D-12 = Dyspnea-12 total score; 6MWD = Six-Minute Walk Distance; CaO₂ was calculated using post-exercise SpO₂; ball = Incentive spirometer ball-lift count.

Note 2: ***=p<0.001

At baseline, most physiological indicators (Hb, Hct, heart rate, SpO₂, and CaO₂) were within normal ranges. The mean 6MWD was 346.7 meters. The PCFS scale scores showed statistically significant differences across groups (*p* = 0.029), whereas no significant differences were found in other baseline indicators (*p* > 0.05) (Table 2).

### Within-group analysis

1 Comparison of Post-COVID-19 Functional Status Scale Within Each Group

Post-intervention comparisons of the PCFS scale demonstrated significant improvements across all EGs. EG1 experienced the most improvement, with scores decreasing from 1.6 to 0.58 (p < 0.001). The other experimental groups also exhibited statistically significant changes (p < 0.05). In contrast, the CG showed no significant change, with scores decreasing from 1.2 to 1.0 (p = 0.343). The trend indicates that earlier intervention is associated with better improvement in functional status, as demonstrated by more significant reductions in the PCFS scale. This emphasizes the importance of timely intervention in maximizing rehabilitation outcomes (Table 3).

**Table 3:** Results of within and between analysis compare of Groups.

	CG	EG1	EG2	EG3	EG4	*P value*
***Primary outcomes***						
**Pre-test PCFS scale [mean (SD)]**	1.2(0.42)	1.6(0.54)	1.9(0.57)	1.3(0.49)	1.7(0.68)	0.029*
**Post-test PCFS scale [mean (SD)]**	1(0.82)	0.6(0.63)	1.1(0.74)	0.5(0.52)	0.9(0.74)	0.099
**△**	−0.2	−1	−0.8	−0.8	−0.8	–
***p value***	0.343	<0.001***	0.003**	0.002**	0.011*	–
**Pre-test D-12[mean (SD)]**	8.2(7.47)	8.3(7.54)	10.2(9.58)	6(5.43)	12.8(7.13)	0.285
**Post-test D-12[mean (SD)]**	6.7(8.67)	1.8(3.04)	4.2(7.27)	2.3(3.65)	5.5(4.93)	0.028*
**△**	−1.5	−6.5	−6	−3.7	−7.3	–
***p value***	0.463	<0.001***	0.008**	0.011*	0.001**	–
**Pre-test D-12 of physiological items** **[mean (SD)]**	4.1(3.76)	5.1(4.01)	5.9(4.86)	3.4(2.19)	7.1(3.32)	0.196
**Post-test D-12 of physiological items** **[mean (SD)]**	3.5(5.02)	1.3(1.78)	2.8(3.88)	1.3(1.92)	3(2.31)	0.044*
**△**	−0.6	−3.8	−3.1	−2.1	−4.1	–
***p value***	0.546	<0.001***	0.007**	0.014*	<0.001***	–
**Pre-test D-12 psychological items** **[mean (SD)]**	4.1(3.99)	3.2(4.05)	4.3(5.12)	2.6(3.9)	5.7(4.48)	0.403
**Post-test D-12 psychological items** **[mean (SD)]**	3.2(3.97)	0.6(1.53)	1.4(3.44)	1(1.76)	2.5(3.06)	0.017*
**△**	−0.9	−2.6	−2.9	−1.6	−3.2	–
***p value***	0.434	<0.001***	0.028*	0.086	0.016*	–
**Pre-test CaO2[mean (SD)]**	14.9(2.02)	15.3(2.78)	16.1(2.45)	15(2.37)	15.3(2.86)	0.872
**Post-test CaO2[mean (SD)]**	15.4(2.52)	15.5(2.75)	14.7(2.25)	16.3(1.27)	16.1(1.81)	0.585
**△**	0.5	0.2	−1.4	1.3	0.8	–
***p value***	0.299	0.514	0.046*	0.061	0.104	–
***Secondary outcomes***						
**Pre-test 6MWD [mean (SD)]**	378.1(61.57)	340.1(71.9)	294.5(82.05)	374.1(53.85)	363.1(123.7)	0.088
**Post-test 6MWD [mean (SD)]**	359.6(113.8)	391.1(81.24)	354.2(84.9)	393.3(49.28)	375.5(110.83)	0.664
**△**	−18.5	51	59.7	19.2	12.4	–
***p value***	0.573	<0.001***	0.027*	0.068	0.172	–
**Pre-test Hb [mean g/dL(SD)]**	11.3 (1.6)	11.5(2.12)	12.2(1.88)	11.5(1.82)	11.6(2.19)	0.88
**Post-test Hb [mean g/dL (SD)]**	11.6(1.94)	11.7(2.06)	11.2(1.68)	12.3(1.06)	12.2(1.42)	0.571
**△**	0.3	0.2	1	0.8	0.6	–
***p value***	0.327	0.513	0.037*	0.082	0.111	–
**Pre-test Hct [mean% (SD)]**	33.9(4.78)	34.8(6.25)	36.7(5.63)	34.7(5.42)	34.9(6.58)	0.874
**Post-test Hct [mean% (SD)]**	34.8(5.56)	35.2(6.25)	33.7(5.05)	37(3.22)	36.8(4.21)	0.582
△	0.9	0.6	3	2.3	1.9	–
***p value***	0.42	0.463	0.036*	0.085	0.111	–
**Pre-test Hb [mean g/dL(SD)]**	11.3 (1.6)	11.5(2.12)	12.2(1.88)	11.5(1.82)	11.6(2.19)	0.88
**Post-test Hb [mean g/dL (SD)]**	11.6(1.94)	11.7(2.06)	11.2(1.68)	12.3(1.06)	12.2(1.42)	0.571
**△**	0.3	0.2	1	0.8	0.6	–
***p value***	0.327	0.513	0.037*	0.082	0.111	–
**Pre-HR (before 6MWT) [mean (SD)]**	88.1(13.26)	83.7(12.59)	85.4(12.69)	77.9(10.26)	91.4(14.17)	0.129
**Post-HR (before 6MWT) mean (SD)]**	83.5(13.13)	79.7(11.44)	84.8(14.25)	78.3(11.15)	91.9(17.92)	0.077
**△**	−4.6	−4	−0.6	0.4	0.5	–
***p value***	0.284	0.019*	0.917	0.867	0.905	–
**Pre-SpO2 (before 6MWT)** **[mean% (SD)]**	98.7(0.95)	98.7(0.63)	98.6(0.84)	98.8(0.39)	98.4(0.7)	0.63
**Post-SpO2 (before 6MWT)** **[mean% (SD)]**	97.7(2.54)	98.7(0.72)	98.9(0.32)	98.9(0.29)	98.9(0.32)	0.037*
**△**	−1	0	0.3	0.1	0.5	–
***p value***	0.128	1	0.343	0.339	0.096	–
**Pre-HR (after 6MWT)** **[mean (SD)]**	104.1(25.31)	99.6(14.62)	97.7(15.3)	93.1(7.62)	107(17.46)	0.286
**Post-HR (after 6MWT)** **[mean (SD)]**	99.9 ± 17.83	98.1(14.42)	105.9(22.56)	93.3 ± 13.61	107.5(18.88)	0.22
**△**	−4.2	−1.5	8.2	0.2	0.5	–
***p value***	0.464	0.462	0.238	0.926	0.884	–
**Pre-SpO2 (after 6MWT)** **[mean% (SD)]**	98.6(0.97)	98.6(0.76)	98.3(1.16)	97.5(2.81)	98.2(0.92)	0.159
**Post-SpO2 (after 6MWT)** **[mean% (SD)]**	98.8(0.63)	98.6(1.48)	98.4(1.08)	98.5(1.45)	98.4(1.08)	0.747
**△**	0.2	0	0.1	1	0.2	–
***p value***	0.168	0.78	0.868	0.197	0.509	–
**Pre-test ball [mean (SD)]**	N/A	2(0.87)	2(0.82)	1.8(0.87)	1.9(1)	0.834
**Post-test ball [mean (SD)]**	N/A	2.8(0.45)	2.7(0.68)	2.8(0.62)	2.9(0.32)	0.906
**△**	N/A	0.8	0.7	1	1	–
***p value***	N/A	<0.001***	0.025*	0.002**	0.008**	–

Note 1: Hb = Hemoglobin; Hct = Hematocrit; PCFS = Post-COVID-19 Functional Status total score; D-12 = Dyspnea-12 total score; 6MWD = Six-Minute Walk Distance; CaO₂ was calculated using post-exercise SpO₂; ball = Incentive spirometer ball-lift count; △ = Difference between pre- and post-test values. *P value* = The statistical significance of between-group differences; *p value*=Within-group pre–post differences

Note 2: *=*p*<0.05； **=*p*<0.01；***=*p*<0.001

2 Comparison of Dyspnea-12 Scores Within Each Group

The pre- and post-intervention comparisons of D-12 scores showed significant reductions across all EGs, thereby indicating improvements in perceived dyspnea symptoms. EG1 exhibited the most significant reduction, with scores decreasing from 8.3 to 1.84 (p < 0.001). The other experimental groups also experienced statistically significant improvements (p < 0.05). In contrast, the CG did not exhibit a statistically significant change in D-12 scores, with values decreasing from 8.2 to 6.7 (p = 0.463).

These findings indicate that earlier intervention is associated with more significant improvements in dyspnea symptoms. The post-test mean score of 1.84 for EG1 approached the threshold, indicating the absence of symptoms, highlighting the potential benefits of early rehabilitation in relieving symptoms.

3 Comparison of Cardiopulmonary Function

Pre- and post-intervention comparisons were conducted within each of the five groups. No statistically significant changes were observed in the CG. For example, the 6MWD decreased from 378.1 to 359.6 meters (p = 0.573), and the resting heart rate declined from 88.1 to 83.5 beats per minute (bpm) (p = 0.284), indicating no meaningful variation (p > 0.05). In contrast, EG1 demonstrated the most prominent improvements. Resting heart rate significantly decreased from 83.7 to 79.7 bpm (p = 0.019), 6MWD increased significantly from 340.1 to 391.1 meters (p < 0.001), and inspiratory ball counts improved from 2.0 to 2.8 (p < 0.001). EG2 also demonstrated significant improvements in resting heart rate and 6MWD (p < 0.05) but concurrently experienced slight reductions in hematological indices such as Hb, Hct, and CaO₂. EG3 and EG4 showed significant improvements only in inspiratory volume: EG3 increased from 1.8 to 2.8 (p = 0.002), and EG4 from 1.9 to 2.9 (p = 0.008). No other physiological or functional indicators in these groups showed statistically significant changes.

Overall, the respiratory training intervention yielded the most significant improvements in participants who had recovered from COVID-19 within 3 months. These participants demonstrated significant enhancements in physical endurance and inspiratory capacity, along with a trend toward reduced heart rate. In contrast, the improvements in the other EGs were more limited, suggesting that the timing of the intervention plays a critical role in determining its therapeutic efficacy.

### Between-group comparisons

1 Between-Group Comparisons of PCFS Scores

At baseline, the PCFS scale differed significantly among all groups (p = 0.029), and the CG showed better functional status than that of EG1, EG2, and EG4 (mean score: CG = 1.2, EG1 = 1.6, EG2 = 1.9, and EG4 = 1.7). However, after 6 weeks of intervention, these differences were no longer statistically significant (*p* = 0.099).

2 Between-Group Comparisons of D-12

No significant differences were observed in D-12 scores among all groups at baseline (*p* = 0.285). However, post-intervention scores showed a statistically significant difference (*p* = 0.028). Further analysis revealed significant differences between the CG and EG1 (*p* = 0.006), CG and EG3 (*p* = 0.040), and between EG1 and EG4 (*p* = 0.036).

3 Between-Group Comparisons of Oxygenation-Related Parameters

No significant between-group differences were observed in blood parameters (Hb and Hct), physiological indices (pre-activity and post-activity heart rate), or oxygenation indicators (CaO₂ and SpO₂) at pre-intervention and post-intervention (p > 0.05), except for pre-activity SpO₂ at the post-test, which showed a significant difference among groups (p = 0.037). Further analysis revealed that the CG exhibited lower SpO₂ than that of all EGs at the post-test.

4 Between-Group Comparisons of 6MWD

No significant differences were observed in 6MWD at either pre-test (p = 0.088) or post-test (*p* = 0.664) in between-groups. Nonetheless, the within-group analysis revealed significant improvements in EG1 (*p* < 0.001) and EG2 (*p* = 0.027), with a trend toward improvement also observed in other EGs. Conversely, CG showed a decline in 6MWD. The initial difference between CG and EG2 at baseline (*p* = 0.018) was no longer present post-intervention (*p* = 0.890), suggesting that the intervention timing may influence physical endurance recovery (Table 3).

### Generalized Estimating Equations (GEEs) Analysis of Group-by-Time Interactions

1 Interaction Between Group and Time on the PCFS Scale

GEE analysis of the group-by-time interaction effects on the PCFS scale revealed statistically significant differences in change between the EGs and the CG following the intervention. Compared with the CG, significant reductions in PCFS scores were observed in EG1 (p < 0.001, B = −3.487), EG2 (p = 0.026, B = −2.754), EG3 (p = 0.012, B = −2.892), and EG4 (p = 0.047, B = −2.387), indicating greater functional recovery in the intervention groups (Table 4).

**Table 4:** Interaction effects between group and time.

	B	95% Wald CI	
		Lower	Upper
***PCFS Scale***			
EG4	−0.600	−1.194	−0.006
EG3	−0.633	−1.171	−0.095
EG2	−0.600	−1.126	−0.074
EG1	−0.823	−1.255	−0.392
CG	–		
***D-12***			
EG4	−5.800	−10.760	−0.840
EG3	−2.167	−6.916	2.583
EG2	−4.500	−9.460	0.460
EG1	−4.965	−8.859	−1.071
CG	–		
***Oxygenation Parameters***			
EG4	0.337	−1.321	1.995
EG3	0.719	−0.868	2.307
EG2	−1.832	−3.490	−0.173
EG1	−0.278	−1.588	1.031
CG	–		
***6MWD***			
EG4	30.990	−7.620	69.600
EG3	37.718	0.752	74.685
EG2	78.260	39.650	116.870
EG1	68.498	37.989	99.007
CG	–		

*=*p*< 0.05; ***=*p*<0.001

Note 1: The β coefficients represent the interaction effects (group × time) estimated from the GEE model. The model included group, time, and group × time as independent variables. The interaction terms indicate the differences in the magnitude of change before and after the intervention between each experimental group and the control group.

2 Interaction Between Group and Time on D-12

GEE analysis of the group-by-time interaction effects on D-12 scores demonstrated statistically significant differences in change between the EGs and the CG following the intervention. Compared with the CG, significant reductions in D-12 scores were observed in EG1 (p = 0.012, B = −4.965) and EG4 (p = 0.022, B = −5.800), indicating greater improvements in perceived dyspnea in these intervention groups. EG2 showed a reduction relative to the CG (B = −4.500), although this difference did not reach statistical significance (p = 0.075). No statistically significant difference in change was observed between EG3 and the CG (p = 0.371, B = −2.167) (Table 4).

3 Interaction Effects on Oxygenation Parameters

GEE analysis of the group-by-time interaction effects on oxygenation-related parameters demonstrated that only EG2 showed a statistically significant difference in change compared with the CG following the intervention (p = 0.030, B = −1.832). No significant differences in change relative to the CG were observed in EG1 (p = 0.677, B = −0.278), EG3 (p = 0.374, B = 0.719), or EG4 (p = 0.690, B = 0.337). Although EG2 exhibited a relative decline in oxygenation compared with the CG, participants in this group still demonstrated improvements in functional status and physical performance. The clinical significance of this seemingly paradoxical finding warrants further investigation (Table 4).

4 Interaction Between Group and Time on 6MWD

GEE analysis of the group-by-time interaction effects on 6MWD demonstrated that, relative to the CG, EG1 (p < 0.0001, B = 68.498), EG2 (p < 0.0001, B = 78.260), and EG3 (p = 0.046, B = 37.718) showed statistically significant greater improvements in 6MWD following the intervention. In contrast, the change observed in EG4 was not statistically different from that of the CG (p = 0.116, B = 30.990). These findings indicate that the intervention was associated with greater improvements in physical performance in most experimental groups compared with the CG (Table 4).

### Item-level analysis of D-12 responses across groups

A detailed item-level analysis of the D-12 revealed no statistically significant changes in total or individual item scores in the CG (*p* > 0.05). In EG1, all items showed significant improvement except for Item 6 (“My breathing is uncomfortable”), which was not statistically significant. This item demonstrated significant improvement in the other EGs, suggesting that symptom responsiveness may vary depending on the timing of the intervention.

EG1 exhibited the most pronounced pre-to-post differences across most items (*p* < 0.0001). As the intervention was administered later in EG2 and EG3, the magnitude of improvement declined; however, a significant resurgence was observed in EG4, forming a bimodal trend. This pattern warrants further investigation into the potential roles of recovery motivation and intervention timing.

Additionally, an analysis of score composition revealed that, both before and after the intervention, the scores of physiological items (Items 1–6) were consistently higher than those of psychological items (Items 7–12) across all groups. This indicates that individuals with Long COVID experience greater distress from the physiological aspects of dyspnea than from the psychological components (supplementary table 1)

## Discussion

The findings indicate that IS is an effective, safe, and low-cost respiratory training modality for improving dyspnea and functional status in individuals with Long COVID. IS significantly alleviated subjective dyspnea and enhanced overall functional capacity in post-COVID-19 participants. In terms of functional status (PCFS scale) and dyspnea severity (D-12), the EGs demonstrated significantly greater improvements after the intervention compared with the CG, reaching statistical significance; in contrast, the CG did not show statistically significant changes.

A study reports that the PCFS scale is highly correlated with other indicators, such as quality of life and mental health [25]. In the present study, all EGs demonstrated statistically significant improvements in functional status following the intervention, with notable reductions in limitations to daily activities. These findings align with those of prior research, which supports the efficacy of respiratory training in enhancing overall function and quality of life for post-COVID-19 [38–42]. Collectively, this study confirms that IS is a practical, low-risk respiratory training modality that effectively reduces subjective dyspnea and improves functional limitations among COVID-19 survivors. The therapeutic benefits were most pronounced with early implementation, underscoring the clinical significance of timely intervention.

Based on D-12 scale assessments, all EGs showed improvements in perceived dyspnea post-intervention. In addition, the generalized estimating equation (GEE) analysis examining the time × group interaction demonstrated that improvements in dyspnea severity were statistically significant in both the EG1 and EG4 groups. Moreover, during the telephone follow-up conducted in week 3, many participants reported noticeable relief from dyspnea within three weeks of initiating IS training, along with enhanced subjective comfort and adherence. These findings align with those of Altmann et al. [43], who report that earlier initiation of respiratory rehabilitation leads to superior pulmonary recovery and overall clinical outcomes. Similarly, studies by Kusumawardani et al [44] and Abo Elyazed et al. [45] support the superiority of IS over other respiratory training modalities in improving pulmonary function.

Dyspnea and functional limitations are closely linked to psychological well-being, as persistent symptoms can significantly impair quality of life and contribute to emotional distress. Studies suggest that prolonged dyspnea in post-COVID-19 patients may stem from direct viral effects on the central nervous system or from a bidirectional interaction between physiological dysfunction and psychological stressors [46–49].

Harenwall et al. [50] and Abelson et al. [51] propose that chronic dyspnea may result in overstimulation of the hypothalamic-pituitary-adrenal axis, leading to dysregulation that exacerbates respiratory symptoms. Conversely, Gudivada et al. [21] report that respiratory training could alleviate psychological disturbances such as anxiety and depression commonly observed during post-COVID-19 recovery. Furthermore, Bai et al. [52] and Malesevic et al. [53] show that gender-related differences in coping strategies significantly influence respiratory and psychological outcomes. Female patients are more likely to report pronounced symptoms and higher levels of psychological distress [54,55]. Huang et al. [56] further attribute these effects to a combination of factors, including delayed physical recovery, social isolation, and financial strain following infection.

Regarding the timing of IS-based respiratory training and its influence on the improvement of Long COVID symptoms, a bimodal trend was observed in the D-12 analysis. Although the earliest intervention group (EG1) exhibited the greatest improvement (*p* < 0.001), the therapeutic effect declined modestly in EG2 and EG3 before resurging significantly in EG4 (9–12 months post-recovery). Further analysis revealed that EG4 participants exhibited higher baseline levels of perceived dyspnea and psychological distress—particularly in the item “My breathing is distressing,” which averaged 1.2 points—suggesting that persistent symptoms may exacerbate anxiety, thus enhancing motivation to actively engage in respiratory training.

McGregor et al. [57] also report a similar bimodal pattern in the effectiveness of interventions for Long COVID, with the most significant improvements observed at 3 and 12 months post-intervention, while outcomes around 6 months were less pronounced. Studies also confirm the persistence of anxiety and depression among COVID-19 survivors, with a higher prevalence in female patients [58–60]. Hanania et al. [50] propose that anxiety and dyspnea may form a vicious cycle wherein activity avoidance behaviors exacerbate respiratory symptoms. Beyond its physiological benefits, respiratory training may also alleviate psychological symptoms by modulating emotional stress pathways via vagal nerve activation [61]. Moreover, psychological expectation and placebo effects have been suggested as potential contributors to symptom improvement [62].

In contrast, the improvement observed in EG3 (6–9 months post-recovery) was relatively modest. Participant data revealed that this group exhibited milder initial symptoms, and 58% had taken NRICM101 (Traditional Chinese Medicine, Qing-guan Yi-hau) during their COVID-19 infection.

Developed by the National Research Institute of Chinese Medicine in Taiwan, NRICM101 is widely used across the region. Previous studies report its antiviral properties, ability to alleviate pulmonary damage in patients with mild COVID-19 [63,64], and potential to enhance cardiopulmonary function and reduce the risk of severe illness [65,66]. These factors may explain the limited post-intervention gains observed in EG3. This study confirms that early intervention with IS yields the greatest improvement in dyspnea. Moreover, a bimodal response pattern was observed, with significant effects also present when intervention occurred 9–12 months post-recovery. Future research is warranted to clarify the psychological drivers and physiological mechanisms underlying this phenomenon, with the goal of optimizing clinical intervention strategies.

However, regarding oxygenation-related parameters, no significant changes were observed in Hb, Hct, SpO₂, or CaO₂ across groups, except for a significant decline in Hb and Hct levels in EG2. These findings suggest that the effect of IS on pulmonary oxygenation capacity may be limited. This observation is consistent with that of Hockele et al. [39], who report that despite no improvement in SpO₂, patients still exhibited significant reductions in dyspnea following inspiratory muscle training.

This outcome may indicate that nonphysiological factors contribute to dyspnea regulation. Hanania et al. [50] propose that anxiety elevates respiratory rate and promotes gas trapping, thereby exacerbating perceived dyspnea. Beyond enhancing lung expansion and ventilation, IS training may also modulate autonomic nervous system activity, potentially alleviating anxiety and stress perception. Furthermore, Lladós et al. [67] report that vagal and phrenic nerve dysfunction is common among COVID-19 survivors, which may contribute to inspiratory muscle weakness and perceived breathlessness. Thus, IS training may mitigate symptoms indirectly by enhancing respiratory muscle function.

After 6 weeks of IS training, EG1 and EG2 exhibited significant improvements in 6MWD, while EG3 and EG4 demonstrated upward trends that were not statistically significant. These outcomes may be influenced by baseline values, intervention timing, and initial symptom severity. These findings align with those of previous studies. Andrea et al. [68] show that IS enhances 6MWD in patients with COPD without prolonging hospitalization. Vallier et al. [69] also report that pulmonary rehabilitation improves 6MWD and quality of life in patients recovering from COVID-19 [70]. Aljazeeri et al. [58] further show that although pulmonary training significantly improves 6MWD, its effects on perceived dyspnea remain limited.

Additionally, in this study, the inspiratory ball count significantly increased across all EGs following the intervention, indicating a significant improvement in inspiratory capacity. Srinivasan et al. [24] report that IS training leads to significant increases in forced expiratory volume in one second and forced vital capacity. Gudivada et al. [21] also show that 50% of participants who underwent IS training achieved restored pulmonary function, outperforming those in nonintervention groups. Although direct evidence linking inspiratory ball count to perceived dyspnea is currently lacking, the present findings suggest promising clinical potential. Further research incorporating comprehensive pulmonary function assessments and long-term follow-up is warranted to clarify its physiological relevance and practical applicability.

Given that this study was conducted during the transitional period following the COVID-19 pandemic, when uncertainties regarding recurrent outbreaks and infection control remained substantial, several practical and methodological limitations should be acknowledged. To minimize unnecessary hospital exposure and maintain infection-control precautions, the study was designed primarily as a community- and home-based intervention, which inevitably influenced certain aspects of participant assessment and follow-up. First, recruitment challenges resulted in unequal group distribution, particularly among participants with prolonged Long COVID symptoms, who were less likely to seek intervention. In addition, the control group had a relatively small sample size and was not stratified according to recovery duration, which may have affected between-group comparisons. Participants who withdrew were excluded from the final analysis, and per-protocol rather than intention-to-treat analysis was applied, potentially introducing attrition bias. Furthermore, the use of NRICM101 during acute infection was not controlled for and may have influenced post-COVID recovery trajectories.

Second, several clinical and environmental variables could not be fully controlled. Variability in SARS-CoV-2 variants, intercurrent respiratory symptoms, repeated infections, antiviral treatment exposure, and residual differences in baseline cardiopulmonary status may have influenced intervention responsiveness [71]. Environmental exposures and age-related differences in exercise tolerance were also not specifically adjusted for in the analyses.

Third, the quality of intervention execution may have been influenced by the self-administered nature of IS training and reliance on self-reported adherence. Although standardized instruction and telephone follow-up were provided, variations in technique and compliance may still have introduced performance-related variability. In addition, because of the open-label design and absence of a sham intervention group, placebo and expectation-related effects on subjective symptom outcomes could not be completely excluded.

Another important limitation is the absence of diffusion capacity of the lung for carbon monoxide assessment (DLco). As the present study primarily focused on a simple home-based intervention among non-hospitalized individuals recovering in community settings, comprehensive pulmonary function testing was difficult to perform routinely during the study period. Future studies incorporating DLco and additional pulmonary function parameters may help further clarify the physiological effects of IS intervention.

Finally, most participants had mild-to-moderate Long COVID with relatively preserved functional status. Therefore, the findings may not be generalizable to individuals with severe pulmonary sequelae or advanced functional impairment.

Despite these limitations, this study provides preliminary evidence supporting IS as a safe, simple, and accessible respiratory training strategy for improving dyspnea and functional status in individuals with Long COVID. Future studies should incorporate larger multicenter cohorts, comprehensive pulmonary assessments, and longer follow-up periods to further clarify the optimal timing and mechanisms of IS intervention.

## Conclusion

This study evaluated the effects of a six-week IS training program on dyspnea and functional status in individuals with respiratory-related Long COVID symptoms. The findings suggest that IS is a feasible and low-intensity intervention that may help alleviate persistent respiratory symptoms associated with Long COVID. Participants who initiated IS training within 3 months of recovery demonstrated the greatest improvements, although benefits were also observed in those who began the intervention at later recovery stages.

In conclusion, IS may represent a practical adjunctive strategy for improving dyspnea and functional limitations in individuals with Long COVID. Further large-scale studies incorporating comprehensive pulmonary function assessments are warranted to confirm these findings and clarify the optimal timing of intervention.

## Supporting information

S1 Table Item-Level Analysis of D-12 scale. (DOCX)

S1 File CONSORT checklist and Protocol documents. (PDF)
