# Supplementary material for: Effects of incentive spirometer training on dyspnea and functional status in patients with long COVID
Source: PLoS One. 2026 Jun 22;21(6):e0351553. doi: 10.1371/journal.pone.0351553 (PMC13286201; doi:10.1371/journal.pone.0351553)
Supplement: S1 Table — (DOCX) [file pone.0351553.s001.docx]

| **Supplementary table 1.** Item-Level Analysis of D-12 scale | | | | | | | | | | | | | | |
| --- | --- | --- | --- | --- | --- | --- | --- | --- | --- | --- | --- | --- | --- | --- |
|  |  | **CG** | | **EG1** | | | **EG2** | | | **EG3** | | | **EG4** | |
|  |  | **Mean(SD)** | **Mean(SD)** | | ***P value*** | **Mean(SD)** | | ***P value*** | **Mean(SD)** | | ***P value*** | **Mean(SD)** | | ***P value*** |
| **1. My breath does not go in all the way.** | **Pre-test** | 0.9(0.88) | 1.1(0.81) | | 0.465 | 1(0.67) | | 0.766 | 0.7(0.49) | | 0.469 | 0.8(0.63) | | 0.766 |
|  | **Post-test** | 0.9(0.88) | 0.4(0.5) | | 0.02* | 0.6(0.7) | | 0.248 | 0.3(0.5) | | 0.024* | 0.3(0.48) | | 0.022* |
|  | **△** | 0 | 0.7 | | - | -0.4 | | - | -0.4 | | - | -0.5 | | - |
|  | ***p value*** | 1 | <0.001*** | | - | 0.037* | | - | 0.104 | | - | 0.015* | | - |
| **2. My breathing requires more work.** | **Pre-test** | 0.8(0.79) | 0.9(0.91) | | 0.78 | 0.9(0.99) | | 0.794 | 0.5(0.67) | | 0.413 | 1.3(0.68) | | 0.193 |
|  | **Post-test** | 0.5(0.97) | 0.2(0.37) | | 0.102 | 0.6(0.97) | | 0.701 | 0.2(0.39) | | 0.183 | 0.5(0.53) | | 1 |
|  | **△** | -0.3 | -0.7 | | - | -0.3 | | - | -0.3 | | - | -0.8 | | - |
|  | ***p value*** | 0.279 | <0.001*** | | - | 0.193 | | - | 0.166 | | - | <0.001*** | | - |
| **3. I feel short of breath.** | **Pre-test** | 0.8(0.79) | 1(0.89) | | 0.554 | 1.1(0.99) | | 0.43 | 0.8(0.62) | | 0.891 | 1.2(0.79) | | 0.294 |
|  | **Post-test** | 0.6(0.97) | 0.2(0.43) | | 0.066 | 0.4(0.7) | | 0.428 | 0.3(0.45) | | 0.149 | 0.5(0.53) | | 0.692 |
|  | **△** | -0.2 | -0.8 | | - | -0.7 | | - | -0.5 | | - | -0.7 | | - |
|  | ***p value*** | 0.443 | <0.001*** | |  | 0.01* | | - | 0.007** | | - | 0.01* | | - |
| **4. I have difficulty catching my breath.** | **Pre-test** | 0.5(0.71) | 0.9(0.86) | | 0.216 | 1.3(0.95) | | 0.033* | 0.8(0.62) | | 0.48 | 1.3(0.82) | | 0.033* |
|  | **Post-test** | 0.5(0.97) | 0.1(0.42) | | 0.07 | 0.5(0.7) | | 1 | 0.3(0.45) | | 0.488 | 0.7(0.53) | | 0.425 |
|  | **△** | 0 | -0.8 | | - | -0.8 | | - | -0.5 | | - | -0.6 | | - |
|  | ***p value*** | 1 | <0.001*** | | - | 0.003** | | - | 0.054 | | - | 0.024* | | - |
| **5. I cannot get enough air.** | **Pre-test** | 0.4(0.7) | 0.8(0.81) | | 0.175 | 0.7(0.82) | | 0.383 | 0.4(0.52) | | 0.959 | 1.2(0.79) | | 0.022* |
|  | **Post-test** | 0.4(0.97) | 0.2(0.48) | | 0.412 | 0.5(0.7) | | 0.7 | 0.2(0.39) | | 0.349 | 0.5(0.53) | | 0.7 |
|  | **△** | 0 | -0.5 | | - | -0.2 | | - | -0.2 | | - | -0.7 | | - |
|  | ***p value*** | 1 | <0.001*** | | - | 0.443 | | - | 0.082 | | - | 0.001** | | - |
| **6. My breathing is uncomfortable.** | **Pre-test** | 0.7(0.68) | 0.6(0.83) | | 0.618 | 0.9(1.1) | | 0.581 | 0.4(0.52) | | 0.415 | 1.2(0.79) | | 0.17 |
|  | **Post-test** | 0.6(0.7) | 0.5(0.26) | | 0.001** | 0.2(0.63) | | 0.037* | 0.1(0.29) | | 0.005** | 0.5(0.53) | | 0.597 |
|  | **△** | -0.1 | -0.1 | | - | -0.7 | | - | -0.3 | | - | -0.7 | | - |
|  | ***p value*** | 0.726 | 1 | | - | 0.025* | | - | 0.039* | | - | 0.01* | | - |
| **7. My breathing is exhausting.** | **Pre-test** | 0.6(0.52) | 0.7(0.9) | | 0.863 | 0.9(0.99) | | 0.429 | 0.4(0.79) | | 0.613 | 1.1(0.74) | | 0.189 |
|  | **Post-test** | 0.6(0.7) | 0.1(0.26) | | 0.002** | 0.3(0.68) | | 0.154 | 0.2(0.39) | | 0.033* | 0.4(0.7) | | 0.34 |
|  | **△** | 0 | -0.6 | | - | -0.6 | | - | -0.2 | | - | -0.7 | | - |
|  | ***p value*** | 1 | <0.001*** | | - | 0.005** | | - | 0.082 | |  | 0.01* | | - |
| **8. My breathing makes me feel depressed.** | **Pre-test** | 0.7(0.82) | 0.5(0.8) | | 0.444 | 0.5(0.85) | | 0.57 | 0.3(0.62) | | 0.184 | 0.8(0.79) | | 0.776 |
|  | **Post-test** | 0.5(0.71) | 0.1(0.37) | | 0.024* | 0.2(0.63) | | 0.188 | 0.3(0.45) | | 0.251 | 0.4(0.7) | | 0.659 |
|  | **△** | -0.2 | -0.4 | | - | -0.3 | | - | 0 | | - | -0.4 | | - |
|  | ***p value*** | 0.443 | <0.001*** | | - | 0.193 | | - | 1 | | - | 0.01* | | - |
| **9. My breathing makes me feel miserable.** | **Pre-test** | 0.5(0.71) | 0.4(0.82) | | 0.642 | 0.6(0.84) | | 0.775 | 0.2(0.58) | | 0.321 | 0.7(0.82) | | 0.568 |
|  | **Post-test** | 0.4(0.7) | 0.1(0.31) | | 0.027* | 0.2(0.63) | | 0.32 | 0(0) | | 0.04* | 0.3(0.68) | | 0.619 |
|  | **△** | -0.1 | -0.3 | | - | -0.4 | | - | -0.2 | | - | -0.4 | | - |
|  | ***p value*** | 0.343 | 0.005** | | - | 0.104 | | - | 0.339 | | - | 0.168 | | - |
| **10. My breathing is distressing.** | **Pre-test** | 0.9(0.74) | 0.8(0.76) | | 0.757 | 1(1.05) | | 0.777 | 0.7(0.78) | | 0.491 | 1.2(0.63) | | 0.397 |
|  | **Post-test** | 0.7(0.68) | 0.3(0.44) | | 0.016* | 0.2(0.42) | | 0.032* | 0.3(0.65) | | 0.098 | 0.6(0.52) | | 0.663 |
|  | **△** | -0.2 | -0.5 | | - | -0.8 | | - | -0.4 | | - | -0.6 | | - |
|  | ***p value*** | 0.443 | <0.001*** | | - | 0.022* | | - | 0.166 | | - | 0.005** | | - |
| **11. My breathing makes me agitated.** | **Pre-test** | 0.7(0.82) | 0.5(0.77) | | 0.46 | 0.7(0.82) | | 1 | 0.5(0.8) | | 0.567 | 1.1(0.99) | | 0.274 |
|  | **Post-test** | 0.5(0.71) | 0.1(0.34) | | 0.01* | 0.2(0.63) | | 0.15 | 0.2(0.39) | | 0.096 | 0.5(0.53) | | 1 |
|  | **△** | -0.2 | -0.5 | | - | -0.5 | | - | -0.3 | | - | -0.6 | | - |
|  | ***p value*** | 0.443 | <0.001*** | | - | 0.052 | | - | 0.166 | | - | 0.051 | | - |
| **12. My breathing is irritating.** | **Pre-test** | 0.7(0.82) | 0.4(0.69) | | 0.223 | 0.6(0.84) | | 0.77 | 0.5(0.8) | | 0.542 | 0.9(0.88) | | 0.559 |
|  | **Post-test** | 0.5(0.71) | 0.1(0.21) | | 0.002** | 0.3(0.68) | | 0.28 | 0.1(0.29) | | 0.02* | 0.3(0.48) | | 0.28 |
|  | **△** | -0.2 | -0.3 | | - | -0.3 | | - | -0.4 | | - | -0.6 | | - |
|  | ***p value*** | 0.443 | 0.002** | | - | 0.193 | | - | 0.054 | | - | 0.005** | | - |
| **Score of physiological items (Items 1–6)** | **Pre-test** | 4.1(3.76) | 5.1(4.02) | | 0.441 | 5.9(4.86) | | 0.296 | 3.4(2.19) | | 0.677 | 7.1(3.32) | | 0.083 |
|  | **Post-test** | 3.5(5.02) | 1.3(1.78) | | 0.022* | 2.8(3.88) | | 0.564 | 1.3(1.92) | | 0.065 | 3(2.31) | | 0.68 |
|  | **△** | -0.6 | -3.8 | | - | -3.1 | | - | -2.1 | | - | -4.1 | | - |
|  | ***p value*** | 0.546 | <0.001*** | | - | 0.007** | | - | 0.014* | | - | <0.001*** | | - |
| **Score of psychological items (Items 7–12)** | **Pre-test** | 4.1(3.99) | 3.2(4.05) | | 0.528 | 4.3(5.12) | | 0.916 | 2.6(3.9) | | 0.402 | 5.7(4.47) | | 0.398 |
|  | **Post-test** | 3.2(3.97) | 1.3(1.53) | | 0.003** | 1.4(3.44) | | 0.099 | 1(1.76) | | 0.036* | 2.5(3.06) | | 0.518 |
|  | **△** | -0.9 | -1.9 | | - | -2.9 | | - | -1.6 | | - | -3.2 | | - |
|  | ***p value*** | 0.434 | <0.001*** | | - | 0.028* | | - | 0.086 | | - | 0.016* | | - |

Note 1: △ = Difference between pre- and post-test values. *P value* = The statistical significance of between-group differences; *p value*=Within-group pre–post differences
Note 2: *=*p*<0.05； **=*p*<0.01；***=*p*<0.001
