# Supplementary material for: Effects of incentive spirometer training on dyspnea and functional status in patients with long COVID
Source: PLoS One. 2026 Jun 22;21(6):e0351553. doi: 10.1371/journal.pone.0351553 (PMC13286201; doi:10.1371/journal.pone.0351553)
Supplement: S1 File — (PDF) [file pone.0351553.s002.pdf]

Section/topic	No	CONSORT 2025 checklist item description	Reported on page no.
**Title and abstract**			
Title and structured abstract	1a	Identification as a randomised trial	NA
	1b	Structured summary of the trial design, methods, results, and conclusions	P2 line 34
**Open science**			
Trial registration	2	Name of trial registry, identifying number (with URL) and date of registration	P2 line 63
Protocol and statistical analysis plan	3	Where the trial protocol and statistical analysis plan can be accessed	P2 line 137
Data sharing	4	Where and how the individual de-identified participant data (including data dictionary), statistical code and any other materials can be accessed	P29 line 603
Funding and conflicts of interest	5a	Sources of funding and other support (eg, supply of drugs), and role of funders in the design, conduct, analysis and reporting of the trial	P29 line 599
	5b	Financial and other conflicts of interest of the manuscript authors	P30 line 623
**Introduction**			
Background and rationale	6	Scientific background and rationale	P3 line 69
Objectives	7	Specific objectives related to benefits and harms	P3 line 99
**Methods**			
Patient and public involvement	8	Details of patient or public involvement in the design, conduct and reporting of the trial	P4 line 106
Trial design	9	Description of trial design including type of trial (eg, parallel group, crossover), allocation ratio, and framework (eg, superiority, equivalence, non-inferiority, exploratory)	P4 line 111
Changes to trial protocol	10	Important changes to the trial after it commenced including any outcomes or analyses that were not prespecified, with reason	NA
Trial setting	11	Settings (eg, community, hospital) and locations (eg, countries, sites) where the trial was conducted	P4 line 106
Eligibility criteria	12a	Eligibility criteria for participants	P5 line 130
	12b	If applicable, eligibility criteria for sites and for individuals delivering the interventions (eg, surgeons, physiotherapists)	
Intervention and comparator	13	Intervention and comparator with sufficient details to allow replication. If relevant, where additional materials describing the intervention and comparator (eg, intervention manual) can be accessed	P6 line 169
Outcomes	14	Prespecified primary and secondary outcomes, including the specific measurement variable (eg, systolic blood pressure), analysis metric (eg, change from baseline, final value, time to event), method of aggregation (eg, median, proportion), and time point for each outcome	P7 line 196
Harms	15	How harms were defined and assessed (eg, systematically, non-systematically)	NA
Sample size	16a	How sample size was determined, including all assumptions supporting the sample size calculation	P7 line 180
	16b	Explanation of any interim analyses and stopping guidelines	NA
Randomisation:			
Sequence generation	17a	Who generated the random allocation sequence and the method used	P4 line 110
	17b	Type of randomisation and details of any restriction (eg, stratification, blocking and block size)	NA

			Reported on page no.
Allocation concealment mechanism	18	Mechanism used to implement the random allocation sequence (eg, central computer/telephone; sequentially numbered, opaque, sealed containers), describing any steps to conceal the sequence until interventions were assigned	P4 line 110
Implementation	19	Whether the personnel who enrolled and those who assigned participants to the interventions had access to the random allocation sequence	P4 line 110
Blinding	20a	Who was blinded after assignment to interventions (eg, participants, care providers, outcome assessors, data analysts)	P7 line 177
	20b	If blinded, how blinding was achieved and description of the similarity of interventions	NA
Statistical methods	21a	Statistical methods used to compare groups for primary and secondary outcomes, including harms	P7 line 188
	21b	Definition of who is included in each analysis (eg, all randomised participants), and in which group	NA
	21c	How missing data were handled in the analysis	NA
	21d	Methods for any additional analyses (eg, subgroup and sensitivity analyses), distinguishing prespecified from post hoc	NA
**Results**			
Participant flow, including flow diagram	22a	For each group, the numbers of participants who were randomly assigned, received intended intervention, and were analysed for the primary outcome	P8 line 238
	22b	For each group, losses and exclusions after randomisation, together with reasons	P8 line 232
Recruitment	23a	Dates defining the periods of recruitment and follow-up for outcomes of benefits and harms	P8 line 233
	23b	If relevant, why the trial ended or was stopped	NA
Intervention and comparator delivery	24a	Intervention and comparator as they were actually administered (eg, where appropriate, who delivered the intervention/comparator, how participants adhered, whether they were delivered as intended (fidelity))	P6 line 172
	24b	Concomitant care received during the trial for each group	P4 line 109
Baseline data	25	A table showing baseline demographic and clinical characteristics for each group	P9
Numbers analysed, outcomes and estimation	26	For each primary and secondary outcome, by group:<br>● the number of participants included in the analysis<br>● the number of participants with available data at the outcome time point<br>● result for each group, and the estimated effect size and its precision (such as 95% confidence interval)<br>● for binary outcomes, presentation of both absolute and relative effect size	P15 line 350
Harms	27	All harms or unintended events in each group	NA
Ancillary analyses	28	Any other analyses performed, including subgroup and sensitivity analyses, distinguishing pre-specified from post hoc	NA
**Discussion**			
Interpretation	29	Interpretation consistent with results, balancing benefits and harms, and considering other relevant evidence	P19 line 420
Limitations	30	Trial limitations, addressing sources of potential bias, imprecision, generalisability, and, if relevant, multiplicity of analyses	P27 line 532

Citation: Hopewell S, Chan AW, Collins GS, Hróbjartsson A, Moher D, Schulz KF, et al. CONSORT 2025 Statement: updated guideline for reporting randomised trials. BMJ. 2025; 388:e081123.
https://dx.doi.org/10.1136/bmj-2024-081123
© 2025 Hopewell et al. This is an Open Access article distributed under the terms of the Creative Commons Attribution License (https://creativecommons.org/licenses/by/4.0/), which permits unrestricted use, distribution, and reproduction in any medium, provided the original work is properly cited.

*We strongly recommend reading this statement in conjunction with the CONSORT 2025 Explanation and Elaboration and/or the CONSORT 2025 Expanded Checklist for important clarifications on all the items. We also recommend reading relevant CONSORT extensions. See www.consort-spirit.org.

Protocol documents

An official website of the United States government Here’s how you know

National Library of Medicine
National Center for Biotechnology Information

Contact ClinicalTrials.gov

Brief Title
Study on Post-Acute COVID-19 Syndrome in Improvement of COVID-19 Rehabilitated Patients by Respiratory Training

NCT Number
NCT06165835

Unique Protocol Id
A202305044

Go to record in classic site

Edit Mode
Disabled

Protocol Summary
Study Identification
Study Status
Sponsors and Collaborators
Oversight
Study Description
Conditions

Protocol Summary

Study Identification

Organization's Unique Protocol ID
A202305044

Brief Title

Feedback

Study Design

Arms and Interventions

Outcome Measures

Eligibility

Contacts and Locations

IPD Sharing Statement

References

Study on Post-Acute COVID-19 Syndrome in Improvement of COVID-19 Rehabilitated Patients by Respiratory Training

Acronym

Study Type

Interventional

Official Title

Study on Post-Acute COVID-19 Syndrome in Improvement of COVID-19 Rehabilitated Patients by Respiratory Training

Secondary IDs

Edit Study Identification

Study Status

Record Verification Date

March 2025

Overall Recruitment Status

Completed

Study Start Date

July 01 2023

Actual

Primary Completion Date

December 30 2024

Actual

Study Completion Date

December 30 2024

Actual

Edit Study Status

Sponsors and Collaborators

Responsible Party, by Official Title

Principal Investigator

Investigator Name [Username]

Chen, Yao-Hsiang [yhchen]

Investigator Official Title

assistant hand nurse

Investigator Affiliation

Tri-Service General Hospital

Name of the Sponsor

Tri-Service General Hospital

Collaborators

Edit Sponsors and Collaborators

Oversight

U.S. FDA-Regulated Product

U.S. FDA-regulated Drug

No

U.S. FDA-regulated Device

No

Investigational New Drug Application (IND)/Investigational Device Exemption (IDE) Information

U.S. FDA IND/IDE

No

Human Subjects Protection Review

Board Status

Approved

Approval Number

A202305044

Board Name

Tri-Service General Hospital Institutional Review Board

Board Affiliation

Tri-Service General Hospital

Board Contact Information

886-2-87923311

17763

tsghirb@ndmctsgh.edu.tw

No.325,Sec.2, Cheng-Kung Rd. Neihu 11490, Taipei, Taiwan, R.O.C

Data Monitoring Committee

No

FDA Regulated Intervention

No

Edit Oversight

Study Description

Brief Summary

Since the emergence of Corona Virus Disease 2019(COVID-19), it has spread rapidly around the world, And it has had a profound impact on the lives and health of people around the world, most of the COVID-19 positive patients are 3-4 weeks after the onset of infection patients can turn negative and recover, however, increasing observational data suggest that these patients long-term or recurring symptoms such as: fatigue, palpitations, cognitive impairment, dyspnea, anxiety, chest tightness and Pain, etc., symptoms may last at least two months or even longer (for example, up to 12 months), the patient's quality of life and The ability to work and social activities also decline accordingly. Therefore, in addition to the acute symptoms after infection, patients also face the same. The challenge of long-term health sequelae associated with COVID-19.

Detailed Description

Relevant studies have shown that breathing training can improve the exercise capacity, lung function, and respiratory rate of patients who have recovered from COVID-19. Difficult and other aspects are safe and effective. However, because COVID-19 is an emerging disease, the clinical research literature so far has limited Clinical data are still insufficient, and the effect of respiratory training on the breathing and physical function of COVID-19 patients has not been determined, and most of the patients are not living in the hospital. How to carry out simple and effective breathing training to reduce the long-term impact of COVID-19 on patients is worthy of continuous discussion. This study will assist COVID-19 patients to perform respiratory training with incentive spirometer intervention, and collect patients before and after the intervention Oxygen requirements, blood draw values and Post-COVID-19 Functional Status scale(PCFS scale) and other data to

explore and evaluate the effect of interventional breathing training on the improvement of symptoms of new coronary pneumonia, as a reference for future clinical treatment and improvement of symptoms of COVID-19.

Edit Study Description

Conditions

Conditions or Focus of Study

COVID-19

Post-Acute COVID-19 Syndrome

Dyspnea

Incentive Spirometer

Keywords

Edit Conditions

Study Design

Primary Purpose

Treatment

Study Phase

N/A

Interventional Study Model

Parallel Assignment

Model Description

Number of Arms

5

Masking

None (Open Label)

Masking Description

Allocation

Randomized

Enrollment

Actual

90

Edit Study Design

Arms and Interventions

Arms	Interventions
**No Intervention : Experimental**<br>Patients who have recovered from COVID-19	

Arms	Interventions
**Experimental : Three months after recovering from COVID-19**<br>Patients who have from COVID-19 in three months	**Device: breathing training**<br>Breathing training for six weeks.<br>Other Name:<br>• Incentive Spirometer
**Experimental : Six months after recovering from COVID-19**<br>Patients who have from COVID-19 in three to six months	**Device: breathing training**<br>Breathing training for six weeks.<br>Other Name:<br>• Incentive Spirometer
**Experimental : Nine months after recovering from COVID-19**<br>Patients who have from COVID-19 in six to nine months	**Device: breathing training**<br>Breathing training for six weeks.<br>Other Name:<br>• Incentive Spirometer
**Experimental : Twelve months after recovering from COVID-19**<br>Patients who have from COVID-19 in nine to twelve months	**Device: breathing training**<br>Breathing training for six weeks.<br>Other Name:<br>• Incentive Spirometer

Edit Arms and Interventions

Outcome Measures

PRIMARY OUTCOME MEASURES

1. Oxygen requirements, blood draw values

Oxygen requirements, blood draw values

[Time Frame: 6 WEEKS]

Edit Outcome Measures

Eligibility

Accepts Healthy Volunteers

Yes

Sex

All

Gender Based

No

Age Limits

Minimum Age

20 Years

Maximum Age

90 Years

Eligibility Criteria

Inclusion Criteria:

1. Patients who have recovered from COVID-19

Exclusion Criteria:

(1) Have suffered from within one year. Those who have recovered from COVID-19 must present proof of diagnosis, such as a medical certificate or screening results, etc., and ICD10 when seeking medical treatment The diagnostic codes are: U07.1 Confirmed COVID-19 virus infection, U09.0 Severe specific infectious pneumonia (COVID-19), unspecified. (2) adult, aged 20-90 (3) conscious mind and behavior normal mental state examination (Mini-Mental State Examination, MMSE) score > 21 points. (4) Those who can communicate in verbal or non-verbal ways and understand Chinese and Taiwanese. (5) are willing to participate in the research and agree to accept random machine dispatcher

Edit Eligibility

Contacts and Locations

Central Contact Person

YAO-WEN HSU

+

fri13wen@hotmail.com

Central Contact Backup

Yao-Hsiang Chen

- +886923766226

810792me@gmail.com

Study Officials

Yao-Hsiang Chen

Tri Service General Hospital Sonshan Branch

Principal Investigator

Locations
1 Locations

Taiwan

Tri Service General Hospital Sonshan Branch
Taipei City,
, Taiwan 105309
Principal Investigator:

Edit Contacts and Locations

IPD Sharing Statement

Plan to Share IPD
Undecided

Plan Description

Edit IPD Sharing Statement

References

Citations
0 Citations

Links
0 Links

Available IPD and Supporting Information

0 Available IPD and Supporting Information

Edit References

U.S. National Library of Medicine
U.S. National Institutes of Health
U.S. Department of Health and Human Services
HHS Vulnerability Disclosure
