# Supplementary material for: Effects of incentive spirometer training on dyspnea and functional status in patients with long COVID
Source: PLoS One. 2026 Jun 22;21(6):e0351553. doi: 10.1371/journal.pone.0351553 (PMC13286201; doi:10.1371/journal.pone.0351553)
Supplement: Attachment — Submitted filename: Response to Reviewers-0217.pdf [file pone.0351553.s005.pdf]

Reviewer #1: The paper is more understandable now.

1. Why wasn't the control group stratified on the basis of long covid? How long had the control group been suffering from long covid? How could this have affected the results?

Thank you for the reviewer's suggestion. This study employed a quasi-experimental design rather than a fully randomized controlled trial. The primary objective was to compare the effectiveness of incentive spirometry initiated at different recovery time points; therefore, stratification based on recovery duration from Long COVID was applied only within the experimental groups. The control group was designed to observe the natural course of symptom changes over time without intervention, and thus was not further stratified according to the duration of Long COVID.

The duration from COVID-19 infection to enrollment for participants in the control group has been presented in Table 2 for reference.

We acknowledge that the absence of stratification in the control group may have introduced a degree of within-group heterogeneity. However, as recruitment occurred during the later phase of the pandemic, the distribution of Long COVID cases was inherently uneven. Imposing additional stratification criteria might have further reduced the sample size and compromised the feasibility of statistical analyses. Nevertheless, baseline comparisons revealed no significant differences among groups. Therefore, we believe that this design limitation is unlikely to have materially affected the main conclusions. Future larger-scale studies may consider stratified designs to further enhance internal validity. We appreciate the reviewer's insightful suggestion.

2. The tables are now in separate documents - this is hard to review.

Thank you for the reviewer's suggestion. We have now reintegrated all tables into the main manuscript to facilitate review and improve readability.

4. Table 4-7 - these tables are very hard to understand, and interpret. But I am happy to see the p for interaction. Can you please instead integrate the p for interaction into Table 3.

Thank you for the reviewer's valuable suggestion. Original Table 3 presents the between-group comparisons as well as the within-group pre–post differences and their corresponding p-values. In contrast, Tables 4–7 report the results of the generalized estimating equations (GEE) analysis examining the time × group interaction. As these analyses are based on different statistical methods and analytical frameworks, they were not combined into a single table.

However, we acknowledge that presenting Tables 4–7 separately may have affected

the readability and clarity of the results. After considering the reviewer's suggestion, we have consolidated the GEE results from Tables 4–7 into a single table and reorganized them according to primary and secondary outcomes to improve overall clarity and interpretability.

5. Also - re-arrange table 3 into Primary and Secondary outcomes.
Thank you for the reviewer's suggestion. The order of presentation in Table 3 has been revised to distinguish between primary and secondary outcomes.

6. Please integrate the sample size information into the main manuscript. I cannot replicate the sample size calculation - thus more details are needed. With two groups you would need a much larger sample size - I am not clear how you get to 90 total subjects.
Thank you for the reviewer's comment. The sample size estimation in this study was not based on a two-group comparison. Instead, it was calculated according to a five-group design (four experimental groups and one control group) using a repeated-measures within–between interaction framework, with all groups analyzed concurrently. The control group received no intervention. We have clearly specified this design in the Methods section (Line 202-208) to avoid misunderstanding that the study was structured as a two-group comparison trial.

7. the discussion says that "Among these, EG1, who underwent the intervention within 3 months of recovery, showed the greatest improvement, with a 79% reduction in D-12 scores." Is this statistically different than the changes observed in the other EGs? Your reply did not address my question.
Thank you for the reviewer's further clarification. Although EG1 demonstrated the highest percentage reduction in D-12 scores, EG4 showed a relatively larger absolute mean difference between pre- and post-intervention measurements. This may be attributable to more severe baseline symptoms in EG4, resulting in greater room for improvement. However, to avoid overinterpretation, we have revised the wording in the Discussion. The original statement emphasizing that EG1 showed the "greatest" improvement has been removed and replaced with a statement indicating that both EG1 and EG4 demonstrated statistically significant improvements in the GEE analysis (Line 451-453), in order to more accurately reflect the statistical findings.

8. The discussion emphasizes the wrong thing. The first paragraph should talk about whether D-12 change from baseline to follow up was different in the

control group to the intervention group. Within group différences could have occurrence due to usual improvement of symptoms over time.

1. Thank you for the reviewer's important comment. We have revised the first paragraph of the Discussion to explicitly emphasize the difference in change between the EGs and the CG, rather than focusing solely on within-group improvements. (line 436-439)
2. We fully agree that within-group improvement may partly reflect the natural resolution of symptoms over time. Therefore, this potential effect was considered at both the study design and statistical analysis stages. In this study, GEE were used to examine the time × group interaction, allowing us to assess whether the intervention effect was significantly greater than the natural change observed in the CG, thereby minimizing the influence of time effects alone.

9. The second paragraph of discussion: " Moreover, many participants reported noticeable relief from dyspnea within 3 weeks of initiating IS training, along with enhanced subjective comfort and adherence." Where are these results presented? Similarly for results pertaining to the inspiratory ball count?
Thank you for the reviewer's comment. Regarding the statement that "many participants reported noticeable relief from dyspnea within 3 weeks of initiating IS training, along with enhanced subjective comfort and adherence," we have clarified in the revised manuscript that this information was obtained during the mid-intervention telephone follow-up conducted at week 3. During this follow-up, participants provided verbal reports regarding changes in their symptoms and training experiences. (line454-456)
As for the inspiratory ball count, its primary purpose was to serve as a training reference during the intervention, helping participants monitor their inspiratory effort. The corresponding data are presented in Table 3. However, because this measure represents only a semi-quantitative visual feedback provided by the incentive spirometer and does not precisely reflect actual inspiratory volume, and because it was not predefined as a primary or secondary outcome in the study design, it was not described as a formal efficacy outcome in the Results section. Instead, it is presented as supplementary information for reference.

10. The interpretations of the effects in lines 357-389 should emphasize the difference of differences vs the control group.
Thank you for the reviewer's comment. We have revised the interpretation

accordingly, shifting the emphasis from within-group pre–post changes to highlighting the difference-in-differences between the EGs and the CG.

11. Everywhere - the order of results should first describe the primary results.
Thank you for the reviewer's comment. We have reorganized the Results section to present the primary outcomes first, followed by the secondary outcomes, in order to improve clarity and better align with the study design.

12. For the between group differences - should these be adjusted for relevant confounders since the EG groups are not randomized?
Thank you for the reviewer's comment. We agree with the reviewer's perspective. Although randomization was not performed, we made efforts to ensure comparability at baseline, and no significant differences were observed among groups in baseline characteristics (as presented in Table 2). In addition, the GEE approach accounts for within-subject correlations and time effects, which partially addresses potential confounding related to repeated measurements. Therefore, no additional covariate adjustments were included in the primary analysis. We appreciate this suggestion and will take it into consideration in future larger-scale studies.

13. lines 339-40 "indicating that participants in EGs improved their functional status to a comparable functional level as the CG." This is an incorrect interpretation. An equivalence test would need to be conducted to make this statement.
Thank you for the reviewer's correction. We agree that this statement may constitute an overinterpretation, and that the term "comparable" should not be used without conducting an equivalence test. Therefore, we have removed this sentence from the main text to avoid potential misunderstanding.

Reviewer #2: Replies from the authors suffice the questions raised in the review. I appreciate your effort in revising several key areas of the draft.
Thank you very much for the reviewer's recognition. We sincerely appreciate your thoughtful comments and encouragement. We will continue to refine our work in this field and consider conducting larger-scale studies in the future to further strengthen the evidence base.

Reviewer #3: I have reviewed the revised manuscript and the authors' response to my previous concerns regarding the study design and the standard of care provided to the control group. I appreciate the authors'

thorough engagement with these points.

1. Regarding the Standard Care Arm and Pulmonary Rehabilitation: I accept the authors' explanation that at the time of the study, a standardized pulmonary rehabilitation program for Long COVID was not routinely available in clinical practice in Taiwan. This context satisfactorily justifies why pulmonary rehabilitation could not be included as the "standard of care" for the control group. It resolves the ethical and scientific concern regarding the withholding of optimal care, as the study design reflected the real-world clinical reality of that specific period and region.

2. Regarding the Role of Incentive Spirometry (IS): I commend the authors for revising the Introduction to clarify the positioning of the intervention. The added text explicitly states that "although IS cannot replace pulmonary rehabilitation, it may serve as a low-cost, self-administered intervention option... to address existing gaps". This successfully shifts the framing of the study from proposing IS as a substitute for comprehensive rehab to evaluating it as a feasible, accessible alternative for resource-limited settings.

General Observations: The inclusion of Generalized Estimating Equations (GEE) analysis and the expanded Limitations section—addressing comorbidities and pre-existing conditions —have significantly strengthened the rigor of the manuscript. The revisions have adequately addressed my methodological concerns.

I have no further comments and recommend the manuscript for publication.

Thank you very much for the reviewer’s thoughtful comments and encouragement. We truly appreciate your support, and will continue to refine our work and aim to conduct larger-scale studies in the future to further strengthen the evidence in this field.
