# Supplementary material for: Effects of incentive spirometer training on dyspnea and functional status in patients with long COVID
Source: PLoS One. 2026 Jun 22;21(6):e0351553. doi: 10.1371/journal.pone.0351553 (PMC13286201; doi:10.1371/journal.pone.0351553)
Supplement: Attachment — Submitted filename: Response to Reviewer 0520.pdf [file pone.0351553.s007.pdf]

Reviewer #1: Thank you for the revisions.
I think there is a problem with the values for the PCFS interaction terms presented in Table 4. These do not seem to line up with what would be expected given the results presented in Table3. Please verify.
The other values presented are close to what is presented in Table 3.

Reply: Thank you for your careful review and for identifying this issue. After re-examining the analysis, we found that the previous GEE model for the PCFS scale had been conducted using an ordinal-variable approach, whereas Table 3 presented the PCFS results as continuous mean score changes. This difference in modeling strategy may have contributed to inconsistency in the interpretation of the interaction coefficients presented in Table 4.

To improve the consistency and interpretability of the results, we reanalyzed the PCFS outcomes using a continuous-variable GEE model and updated the corresponding interaction coefficients in Table 4 accordingly. We sincerely appreciate the reviewer's careful observation, which helped us further improve the clarity of the manuscript. (P.19 Table4)

Reviewer #3: The authors have successfully maintained the crucial clarifications requested in the previous review rounds, effectively resolving my primary methodological and ethical concerns.
Specifically, the manuscript correctly contextualizes the control arm by acknowledging the absence of standardized pulmonary rehabilitation programs during the study period. This provides a solid ethical and scientific justification for the study design based on the clinical reality of the time.
Furthermore, the authors have appropriately defined the role of Incentive Spirometry. By framing it as a practical, self-administered adjunct rather than a replacement for comprehensive pulmonary rehabilitation, the study offers a highly relevant, low-cost option. This distinction is particularly valuable for optimizing respiratory care in resource-limited settings where formal, multidisciplinary rehabilitation programs may be inaccessible to patients.
The revisions have significantly strengthened the manuscript's clinical applicability and scientific integrity. The authors have adequately addressed all of my feedback, and I have no further concerns. I recommend this article for publication.

Reply: We sincerely thank the reviewer for the thoughtful comments and positive evaluation of our manuscript. Your feedback has been highly valuable in strengthening the clinical applicability and scientific rigor of this study. We are grateful for your encouragement and will continue striving to improve the quality and clinical relevance of our future research.

Reviewer #4: Dear colleagues,
Thank you for giving me the opportunity to review your article. This is an interesting idea but unfortunately, I don't believe that you have scientifically proof your conclusion: „In conclusion, IS represents an effective and practical strategy for alleviating dyspnea and improving functional limitations in individuals with Long COVID.“

The design of your study has many limitations as you have correctly stated in the limitations section but there is also one big limitation and you have not mentioned it. The lung function analysis of the patients with CO diffusion capacity which is known to be the important parameter considering the exercise limitation in post-COVID patients. From my point of view, no studies of post-COVID respiratory investigations should be done or at least published without the DLco data. Also, the intervention itself could have important placebo effect that was not possible to exclude by the study design.
If you can correct for previously stated problems, I would be glad to re-review your article.
Best regards

Reply: We sincerely thank the reviewer for the thoughtful and constructive comments. We agree that the conclusions of our study should be interpreted cautiously given the limitations of the study design. Accordingly, we have revised the limitation and conclusions throughout the manuscript to avoid overly definitive statements regarding the effectiveness of IS intervention.

Regarding the absence of DLco assessment, we fully acknowledge that diffusion capacity is an important parameter when evaluating pulmonary impairment and exercise limitation in patients with post-COVID conditions. However, the primary aim of this study was to evaluate the feasibility and potential benefits of a simple, low-cost, home-based respiratory training

intervention that could be implemented in non-hospitalized individuals recovering from COVID-19.

During the study period (2023–2024), most participants had already returned to community or home settings and were not hospitalized. Performing DLco measurements would have required participants to repeatedly return to the hospital pulmonary function laboratory, which posed practical difficulties and potentially increased unnecessary exposure risks during the post-pandemic period.

In addition, at the time the study protocol was initially designed, there was still limited evidence regarding standardized respiratory assessment strategies for Long COVID. Therefore, DLco measurement was not incorporated into the original study design. We appreciate the reviewer for highlighting this important issue, and we agree that future studies should incorporate comprehensive pulmonary function assessments, including DLco analysis, to further clarify the physiological mechanisms underlying symptom improvement. (P.25 line580-585)

We also agree that placebo effects and expectation-related responses could not be fully excluded because of the open-label design and the absence of a sham intervention group. Therefore, we have further emphasized this issue in the Limitations section and revised the interpretation of the findings accordingly. (P.26 line 577-579)
