# Supplementary material for: Effects of incentive spirometer training on dyspnea and functional status in patients with long COVID
Source: PLoS One. 2026 Jun 22;21(6):e0351553. doi: 10.1371/journal.pone.0351553 (PMC13286201; doi:10.1371/journal.pone.0351553)
Supplement: Attachment — Submitted filename: PLOS Reviews 30.10.2025.docx [file pone.0351553.s003.docx]

PLOS Reviews

| Line | Comment |  |
| --- | --- | --- |
| 101 | Please note that there are no figures in this manuscript, and I believe it should be Table 1. |  |
| 115 | Same as above. I believe you are referring to Table 2. |  |
| 113-114 | I believe pulmonary rehabilitation should be a part of standard care for long COVID syndrome; I would be grateful if the authors could rationalize avoiding pulmonary rehabilitation to the control group. |  |
| 122 | Please clarify the blood tests. |  |
| 125 | Same issue as the comment 1 |  |
| 131 | I would be grateful if you could clarify whether these respiratory symptoms were new onset symptoms or whether they were worsened by COVID infection. |  |
