# Supplementary material for: Effects of incentive spirometer training on dyspnea and functional status in patients with long COVID
Source: PLoS One. 2026 Jun 22;21(6):e0351553. doi: 10.1371/journal.pone.0351553 (PMC13286201; doi:10.1371/journal.pone.0351553)
Supplement: Attachment — Submitted filename: Response to Reviewers.docx [file pone.0351553.s004.docx]

**Review Comments to the Author**

**Reviewer #1:** This reports the results of an RCT that compared the efficacy of IS for improving dyspnea and functional status in post COVID 19 patients. Unfortunately, this paper is impossible to read and understand.
-A ton of outcomes are presented - with no distinction about which are the primary outcomes and which are secondary.
-It is unclear how the randomization was carried out.
-The experimental group is stratified by time since infection, but it is unclear whether this was defined a priori or not.
-The analysis compares within changes from baseline to post intervention in each group. What is important is if these changes are different in the experimental arms vs. the control arm. See suggestions below.

1. How exactly was the randomization carried out? Please see the consort checklist and ensure that all the details are given, in one section. Right now these are scattered throughout the manuscript.

Reply: Thank you for the reviewer’s suggestion. Because Long COVID symptoms tend to improve naturally over time, strict randomization could result in imbalance in disease status at the time of intervention and introduce systematic bias related to spontaneous recovery. In addition, it is not feasible in clinical practice to require patients who actively seek intervention to be randomly assigned to a control group. Therefore, this study adopted a predefined stratification based on recovery duration rather than random allocation. We have added this design consideration and its rationale to the Study Design section. (line124-132)

2. Why was such a high power used for the sample size calculation? What outcome was the sample size based on? What detectable difference was used? What SD was used? Please justify each choice.

Reply: Thank you for the reviewer’s insightful comment. We adopted a power of 0.95 based on the following considerations. **First,** the final analysis of this trial was performed using generalized estimating equations (GEE) with five study groups and a group × time interaction term. Given the nature of Long COVID, patients’ symptoms may improve spontaneously over time, and we anticipated heterogeneous recovery trajectories across the four experimental groups (0–3, 3–6, 6–9, and 9–12 months post-recovery). Such heterogeneity can disperse the overall effect across groups and reduce the effective power for detecting the interaction effect, which is the key parameter of interest in this design. To mitigate this potential dilution of statistical power, we chose a more conservative power level (0.95) to ensure adequate sensitivity for detecting clinically meaningful differences. **Second,** randomized controlled trials in Long COVID populations remain limited, and there is insufficient preliminary evidence to precisely estimate the true effect size of incentive spirometry in this population. Under circumstances in which the expected effect size is uncertain, using a higher power helps reduce the risk of type II error, preventing the study from overlooking a potentially meaningful intervention effect. **In addition,** several prior studies in similar rehabilitation contexts have also adopted a high statistical power (e.g., Power = 0.95) to ensure that clinically meaningful intervention effects can be reliably detected.( Çetin, Ş., Çetin, M., Kaplan, A., Kaplan, Ö., & Çelik, İ. (2024). The Effect of Respiratory Exercises on People with Ongoing Dyspnea and Recovered from COVID-19. EJMA, 4(1), 13-21. <https://doi.org/> 10.14744/ ejma.2023.29491.)

For these reasons, adopting a power of 0.95 was deemed an appropriate and conservative strategy for a multi-group design requiring interaction analyses.
The sample size calculation in this study was based on the primary outcome, the Dyspnea-12 (D-12) score. In studies involving Long COVID–related dyspnea, the variability of the D-12 measure generally falls within a moderate range, with standard deviations commonly reported between 5 and 7 points, which represents a typical variation observed in the literature. (Ekström, M. P., Bornefalk, H., Sköld, C. M., Janson, C., Blomberg, A., Bornefalk-Hermansson, A., Igelström, H., Sandberg, J., & Sundh, J. (2020). Minimal Clinically Important Differences and Feasibility of Dyspnea-12 and the Multidimensional Dyspnea Profile in Cardiorespiratory Disease. Journal of pain and symptom management, 60(5), 968–975.e1. <https://doi.org/10.1016/> j.jpainsymman.2020.05.028).The G*Power parameters adopted in this study are consistent with those later reported in similar respiratory training intervention research. For example, Çetin et al. (2024) employed sample size estimation settings comparable to those used in our study.

3. Please be specific about the primary outcome. Was this 6MT walk at follow up or was it the change from baseline? OR was it dyspnea?

Reply: Thank you for the reviewer’s insightful comment. The primary outcomes of this study were D-12 (Dyspnea-12), PCFS (Post-COVID Functional Status), and CaO₂ (arterial oxygen content). These measures were selected as the primary endpoints because Long COVID is characterized mainly by dyspnea, post-exertional discomfort, and functional limitations. D-12 and PCFS directly assess symptom burden and functional impairment, while CaO₂ reflects physiological oxygenation status. Together, these indicators more accurately capture the direct effects of the respiratory intervention on patients’ respiratory symptoms and functional recovery.

In contrast, the 6-minute walk distance (6MWD) was designated as a secondary outcome. Although 6MWD is widely used to evaluate overall cardiopulmonary endurance, it is an indirect functional measure and can be influenced by multiple non-respiratory factors. For instance, during the early phases of the pandemic in Taiwan, confirmed COVID-19 cases were required to undergo home isolation or stay in designated quarantine facilities. This markedly reduced physical activity levels and led to muscle deconditioning in many individuals. Consequently, lower 6MWD values in some participants may reflect reduced muscle strength or deconditioning rather than impaired cardiopulmonary function alone. Therefore, 6MWD was considered more suitable as a complementary functional measure rather than a primary endpoint.
Accordingly, the primary outcomes in this study were D-12, PCFS, and CaO₂, while 6MWD served as a secondary outcome assessing overall exercise tolerance. (line143-153)

4. Were the groups defined a priori?
Reply: Thank you for the reviewer’s comment. The grouping strategy in this study was defined a priori, prior to the initiation of data collection. Because one of the objectives of the study was to compare whether the effectiveness of incentive spirometry differed across varying recovery durations, the groups were predetermined during the study design phase based on the principle of Long COVID symptom duration stratification. Accordingly, the recovery periods were divided into four stages, rather than being created post hoc after data collection. We have clarified the grouping rationale and criteria in the Methods section. (line 158-167)

5. Please remove the p value from Table 2. Also standardize the number of decimal places. For the continuous variables present mean (SD). Also - please fix the format - it is impossible to read as is. What does a mean 1.8 for gender mean??

Reply: Thank you for the reviewer’s suggestion. Table 2 has been revised accordingly.

6. The primary analysis should compare the change in 6 minute walk between the intervention groups vs the control group overall. And then, the same thing for dyspnea. Then, use a regression model that includes arm and the group, and the interaction between arm and group - the crucial thing is if the effect of the intervention differs by group.

Reply: Thank you for the reviewer’s suggestion. We have revised the footnotes of Table 3 to clearly explain the meaning of the p-values and P-values, allowing readers to directly interpret the pre–post changes in 6MWD and dyspnea measures for each group, as well as the statistical significance of these changes.

In addition, we thank the reviewer for this valuable suggestion. While our study was not originally designed to test a predictive hypothesis (e.g., that a longer duration of triflow training would result in greater improvement), we understand the rationale for exploring such an analysis. Accordingly, we conducted an exploratory regression model including arm, group, and their interaction. However, the results did not show a clear or robust interaction effect. Given this, and considering the exploratory nature of our study, we chose not to emphasize these findings in the main analysis, but have reported them in the below.

| **Model Summary** | | | | | |
| --- | --- | --- | --- | --- | --- |
| Model | Change Statistics | | | | |
|  | R Square Change | F Change | Sig. F Change |  |  |
| D12 | 0.060^a^ | 5.279 | 0.024 |  |  |
| PCFS | 0.101^a^ | 9.318 | 0.003 |  |  |
| CaO2 | 0.001^a^ | 0.101 | 0.752 |  |  |
| 6WMD | 0.113^a^ | 10.191 | 0.002 |  |  |
| Arm | | | | | |
|  |  |  |  |  |  |
| **Model Summary** | | | | | |
| Model | Change Statistics | | | | |
|  | R Square Change | F Change | Sig. F Change |  |  |
| D12 | 0.016^a^ | 1.390 | 0.242 |  |  |
| PCFS | 0.044^a^ | 3.777 | 0.055 |  |  |
| CaO2 | 0.018^a^ | 1.440 | 0.234 |  |  |
| 6WMD | 0.127^a^ | 11.673 | 0.001 |  |  |
| Group | | | | | |
|  |  |  |  |  |  |
| **Tests of Between-Subjects Effects** | | | | | |
| Dependent Variable: | | | | | |
| Corrected Model | Type III Sum of Squares | F | Sig. |  |  |
| D12 | 360.512^a^ | 1.725 | 0.126 |  |  |
| PCFS | 7.468^a^ | 2.455 | 0.032 |  |  |
| CaO2 | 44.637^a^ | 1.676 | 0.139 |  |  |
| 6WMD | 73629.524^a^ | 4.584 | 0.001 |  |  |
| arm * group2 | | | | | |

7. How exactly were the GEE fit? What correlation structure was used? Were robust standard errors used? Table 4 presents group by time interaction - this is a little better - now we can see that some of the groups have different changes than the control group.

Reply: Thanks to the reviewer for the insightful comments. The GEE model in this study was constructed using a repeated-measures framework to evaluate changes before and after the intervention across the five study groups, as well as the group × time interaction. An autoregressive correlation structure of order 1 (AR(1)) was applied, and robust standard errors were used to ensure stable parameter estimation even if the working correlation structure was not perfectly specified.

8. Table 8 is extraneous, and not part of the objectives. Remove all these results.

Reply: Thank you for the reviewer’s suggestion. Table 8 has been removed from the main text and placed in the Appendix as supplementary table 1. (line 409)

9. the discussion says that "Among these, EG1, who underwent the intervention within 3 months of recovery, showed the greatest improvement, with a 79% reduction in D-12 scores." Is this statistically different than the changes observed in the other EGs?

Reply: Thank you for the reviewer’s comment. The improvement in D-12 was calculated as (post − pre) / pre × 100%, and the corresponding values are presented in Table 3. EG1 showed a 79% reduction, whereas EG2, EG3, and EG4 showed reductions of 59%, 62%, and 57%, respectively, indicating that EG1 demonstrated the greatest relative improvement among the experimental groups. We have referenced Table 3 in the main text for readers’ convenience. (line 418)

**Reviewer #2: This is an excellent article focusing on a highly relevant and common issue currently encountered in pulmonary and medicine clinics.**

The study effectively evaluates the use of incentive spirometry as a component of respiratory training to relieve symptoms associated with long COVID. While there are currently no established treatment alternatives proven to resolve long COVID, respiratory training—including techniques like incentive spirometry—has long been utilized in the management of other chronic lung diseases. This approach aims to improve lung function, reduce dyspnea, and enhance overall functional status, suggesting that such interventions may play a valuable role in the multidisciplinary management of post-COVID patients

The study features an innovative approach by employing multiple experimental groups alongside a control group. These groups are evaluated longitudinally, according to the duration elapsed since recovery from COVID-19. This design allows for assessment of intervention effectiveness over time, providing valuable insights into how respiratory training may impact functional recovery and symptom resolution in the post-COVID population.

A key strength of the study lies in the intervention's simplicity, which is easy to implement and associated with limited to no immediate perceived harm to the patient. Moreover, the fact that incentive spirometry can be self-performed by patients adds to its practicality and feasibility, making it an attractive option for managing long COVID symptoms in the outpatient setting. The sample size for this study was appropriately calculated using statistical methods, including repeated measures one-way ANOVA. The investigators also accounted for a potential 10% dropout rate by enrolling an adequate number of additional participants. This approach enhances the robustness of the findings and ensures that sufficient data would be available for meaningful analysis despite potential attrition.

The diagnosis of long COVID is a complex and nuanced topic that warrants discussion on its own. Unlike many other conditions, long COVID remains primarily a diagnosis of exclusion, as there is currently no definitive imaging or blood-based test to confirm its presence. Clinicians must therefore rely on clinical evaluation, history, and the persistence of characteristic symptoms beyond the acute phase of infection, while actively ruling out alternative explanations. This diagnostic uncertainty presents a significant challenge in both research and clinical practice.

A significant limitation of the study is its lack of consideration for the inherent severity or risk posed by pre-existing cardiopulmonary disease. For example, the study does not account for whether participants’ initial COVID-19 illness was complicated by respiratory failure, myocarditis, hypoxemic respiratory failure, or pulmonary embolism. Instead, it includes individuals who have recovered from COVID-19 within the past year, without stratifying them according to the severity or type of their prior cardiopulmonary involvement. A mean 6-minute walk distance of 347 meters indicates that the study group consisted of a relatively healthy population with moderate baseline functional capacity. This may limit the applicability of the findings to individuals with more severe impairment or lower exercise tolerance. Subjective performance in interventions such as respiratory training may vary significantly with age, as younger patients often tolerate these exercises better than older individuals. There is no age-based stratified analysis to address these potential differences. Furthermore, the study does not fully account for underlying health risk factors such as pre-existing cardiopulmonary disease and environmental exposures, including urban air pollution. Although hypertension and cardiovascular disease were the most reported underlying medical conditions among participants, it remains uncertain whether individuals with chronic lung diseases were excluded. These factors could substantially influence both baseline functional status and response to intervention, and their omission may limit the generalizability of the study’s findings. The study comments on baseline exercise tolerance, noting that 40% of participants did not engage in regular physical activity. However, it is unclear whether this lack of activity reflects participants’ habits prior to their COVID-19 episode or is a consequence of their illness and recovery. This ambiguity makes it difficult to interpret the impact of the intervention on overall exercise capacity and recovery trajectories. Although participants of the study have at least 1 or 2 episodes of covid infection, the study does not address whether outcomes differed between individuals with repeated COVID-19 infections compared to those who experienced a single episode. This information is not provided, leaving uncertainty about whether recurrent infection may impact functional recovery or response to respiratory training in long COVID patients. Injectable antivirals were only used in 3 out of 85 participants (4% of study population). so, I would expect the participants to have a lower acuity of covid infection at base line. What is the difference among those who were treated with the medications vs no medication group (28 of 85)

Reply: Thank you for the reviewer’s valuable comments. This study has indeed presented the major cardiopulmonary comorbidities in Table 1, and chronic obstructive pulmonary disease (COPD) was excluded based on the predefined inclusion and exclusion criteria. However, certain comorbidities that could not be fully identified or controlled—such as undiagnosed mild pulmonary conditions or other underlying health risks—may still influence baseline functional status or response to the intervention. In addition, environmental exposures (e.g., urban air pollution) may serve as potential confounding factors, but related measurements were not available within the scope of our data sources and study design.

We fully agree that the finding that 40% of participants did not engage in regular physical activity may reflect two potential sources: (1) pre-existing lifestyle patterns prior to COVID-19 infection, and (2) reduced activity levels resulting from isolation and the recovery process after infection.

During the pandemic in Taiwan, confirmed cases were required to undergo home isolation or stay in designated quarantine facilities, which prevented most patients from maintaining their usual physical activity during both the acute phase and early recovery. This often led to reduced muscle strength and lower exercise tolerance. Such non-cardiopulmonary factors could directly influence performance on the 6-minute walk distance (6MWD) test, making it difficult to interpret 6MWD as a pure indicator of cardiopulmonary function or intervention effect. This ambiguity is also one of the reasons why 6MWD was not selected as a primary outcome in this study.

At present, the literature has not reached a consistent conclusion regarding whether multiple COVID-19 infections worsen Long COVID symptoms or affect functional recovery (for example, findings from Bowe et al. also indicate ongoing uncertainty). During participant enrollment and group allocation in our study, we made efforts to distribute participants with different numbers of prior infections evenly across the groups, in order to minimize imbalance and reduce the potential influence of infection frequency on comparisons of intervention effects.

In this study, three participants received injectable antiviral therapy. We examined their baseline characteristics and post-intervention improvements, and did not observe notable differences compared with participants who did not receive injectable antiviral agents. However, because the number of such cases was very small, it was not possible to perform statistically meaningful subgroup or stratified analyses.

We have incorporated these considerations into the “Limitations” section of the Discussion to clarify their potential impact on the interpretation and generalizability of the findings. (line 541-571)

Despite robust sample calculation several subgroups had a limited number of patients; detailed interpretation in some of the subgroup analyses was extremely limited.

Reply: Thank you for the reviewer’s comment. We agree that the limited number of participants in some subgroups represents a key limitation and constrains the interpretability of subgroup analyses. Although we carefully attempted to ensure comparable baseline characteristics across groups during enrollment and allocation to preserve group homogeneity and minimize confounding, the small subgroup sample sizes inevitably limited our ability to conduct more detailed stratified analyses.

The effect of the Chinese medicine formula on the results is not clearly addressed in the study. It is unclear whether a separate analysis was conducted for patients who did not receive the Chinese medicine formula, which raises concerns about the generalizability of the findings—particularly to populations that do not use traditional Chinese medicine. Additionally, the possible interaction between the Chinese medicine formula and the intervention being studied (such as respiratory training) remains uncertain. Without subgroup analysis, it is difficult to determine whether the observed improvements in recovery are attributable to the respiratory intervention itself, the Chinese medicine formula, or a combination of both. This limitation should be considered when interpreting the applicability of the study to broader patient populations

Reply: Thank you for the reviewer’s insightful comment. We have already addressed this issue in line 524-533 of the revised manuscript and will take it into careful consideration in future research. In addition, we have added a statement in the Discussion to explicitly acknowledge this as a study limitation, particularly with regard to the potential confounding effects of the Chinese herbal formula and the absence of subgroup analyses among participants who did not receive it. Notably, patients who received NRICM101 (Traditional Chinese Medicine, Qing-guan Yi-hau) were separately identified in Table 2. However, the number of such patients was insufficient to permit a meaningful subgroup analysis. Moreover, during the study period, these patients did not demonstrate any particularly significant treatment effects or distinct clinical symptoms compared with those who did not receive NRICM101.

Quality control in the study was addressed by having a nurse perform the initial training for participants. However, subsequent performance of the intervention relied on patient self-reporting, which introduces the potential for variation in both execution and documentation of the exercise. While telephone follow-up calls were used to assess compliance, a video call could have provided a more objective assessment, allowing direct observation of patient technique without the confounding factor of patients talking during incentive spirometry sessions. Employing video-based monitoring might enhance the accuracy and consistency of performance evaluation in future studies.

Reply: Thank you for the reviewer’s comment. We have incorporated the relevant statements into the limitations section.

The study mentions regular telephone follow-ups; however, it does not specify the exact frequency of these calls. Further clarification regarding the timing and intervals of follow-up contacts would be useful to assess the consistency of monitoring and its potential impact on patient compliance and outcomes.

Reply: Thank you for the reviewer’s comment. We have revised the Study Design section to clearly specify the timing and frequency of the telephone follow-up in order to enhance transparency of the research process. The research team conducted a telephone follow-up at Week 3 of the intervention to assess participants’ adherence, confirm correct use of the incentive spirometer, and identify any discomfort or adverse reactions. These details have now been incorporated into the manuscript to provide readers with a clearer understanding of the follow-up arrangement and its role in monitoring the intervention. (line 124-132)

There appears to be some lack of clarity regarding the final number of participants included in the analysis. The study notes that five patients were lost to follow-up, and an additional three participants, who only completed the questionnaire assessments, were excluded from the final analysis. Based on these exclusions, the total analysis group was stated as 85; however, by calculation, one might expect the final number to be 82. Clarifying the exact criteria for inclusion and ensuring consistency in reporting participant numbers is important for accurate interpretation of study results.

Reply: Thank you for the reviewer’s comment. A total of 90 participants were enrolled in this study, of whom 5 were lost to follow-up, leaving 85 participants who completed at least part of the assessments. Among these 85 participants, 3 completed only the questionnaire assessments but did not complete the physiological measurements. Therefore, their physiological data were excluded from the corresponding analyses, while their questionnaire data remained included. We have revised the manuscript to clarify the inclusion criteria and participant flow to ensure consistency throughout the text. (line 259-262)

Smoking prevalence in Taiwan, where this study was conducted, is low at around 13% overall. This aligns with the study population, in which female smoking prevalence is reported at only 2% and male smoking at 23%. These rates reflect national averages and provide important context for interpreting study outcomes, especially when considering risk factors commonly associated with cardiopulmonary health.

In table 2
When we look at the average duration of post recovery. For experimental Group 2 it is mentioned that the average duration of post recovery is only 1.2 months, I am not sure. If this is represented adequately as experimental, group 2 should be Three to six months. It is unclear why there are numbers in the row beside Hypertension in the mean and SD, I do not think we need this is necessary for categorical variables

Reply: Thank you for the reviewer’s comment. The numerical value has been corrected accordingly. (table2)

Based on the PCFS score, there are significant differences that are noted in the Experimental Groups. It indicates that the study population is a healthier population, and the underlying COVID infection was less severe. Without any long-term Needs for supplemental oxygen. So, I would expect that the patients would not have any diseases like COVID-induced ILD.

Reply: Thank you for the reviewer’s comment. The PCFS scores in our study indicate that the participants generally had relatively preserved functional status, suggesting that most individuals belonged to a milder Long COVID population and were less likely to have conditions such as COVID-induced fibrotic interstitial lung disease. Therefore, the applicability of our findings is primarily limited to patients with mild to moderate symptoms. Caution is warranted when extrapolating these results to individuals with severe disease or significant structural lung impairment. We have added this clarification at limitation. (line 541-571)

There is a paragraph repetition between lines 110 and 120 and this could be corrected.

Reply: Thank you for pointing this out. The duplicated paragraph between lines 110 and 120 has been removed and corrected.

Reviewer #3:

I appreciate the authors' commitment to rigorously evaluating a low-cost strategy for managing Long COVID syndrome, especially given the crucial need for accessible interventions in low and middle-income countries.
However, I have a significant methodological concern regarding the composition of the standard care arm and its relationship to the intervention being tested.
My concern is two-fold:
1. Exclusion of Pulmonary Rehabilitation:
Pulmonary rehabilitation is widely recognized and increasingly accepted as a cornerstone of management for persistent respiratory and functional impairment in Long COVID. Excluding it as a component of standard care for both the control and intervention groups may create a potential ethical and scientific deficit by not providing the currently accepted optimal care to the participants.

Reply: Thank you for raising this important point. During the period in which this study was conducted, there was no standardized pulmonary rehabilitation program for Long COVID patients in Taiwan. Even when patients sought medical care for persistent post-COVID respiratory symptoms, pulmonary rehabilitation was not routinely provided as part of standard clinical practice. Therefore, incorporating pulmonary rehabilitation into the standard care protocol of this trial would not have reflected the actual clinical context at the time.
Moreover, one of the key aims of this study was to evaluate whether incentive spirometry could serve as a low-cost, immediately implementable, and self-administered intervention option in healthcare settings where formal pulmonary rehabilitation services are not available. Prior to initiating the study, we conducted a literature review and confirmed that many regions worldwide similarly lack standardized pulmonary rehabilitation resources for Long COVID patients, underscoring the clinical relevance of investigating such an accessible intervention.

2. Role of Incentive Spirometry:
I believe that both groups should have received a structured pulmonary rehabilitation program. The authors' chosen low-cost intervention, such as incentive spirometry, should have been evaluated as an additive or specific, targeted component in addition to comprehensive pulmonary rehabilitation, rather than a potential replacement for it.
I would appreciate a strong justification from the authors for this trial design choice, particularly on why they elected to avoid including pulmonary rehabilitation in the standard care protocol, which is critical for fully assessing the incremental benefit of their low-cost strategy.

Reply: Thank you for the reviewer’s valuable comment. We fully concur that incentive spirometry (IS) should be considered an adjunctive or targeted intervention, rather than a replacement for pulmonary rehabilitation. Accordingly, this study was not intended to promote IS as an alternative to standard pulmonary rehabilitation, but to assess its feasibility and potential benefit as a supplementary, low-cost, and readily deployable strategy in real-world clinical settings where access to structured pulmonary rehabilitation programs is limited.
We have strengthened this clarification in the Introduction to better define the role of IS and to avoid any misunderstanding that it was intended as a substitute for comprehensive pulmonary rehabilitation. (line107-109)
