# Supplementary material for: Effects of incentive spirometer training on dyspnea and functional status in patients with long COVID
Source: PLoS One. 2026 Jun 22;21(6):e0351553. doi: 10.1371/journal.pone.0351553 (PMC13286201; doi:10.1371/journal.pone.0351553)
Supplement: Attachment — Submitted filename: Response to Reviewers 0420.docx [file pone.0351553.s006.docx]

Review Comments to the Author

Please use the space provided to explain your answers to the questions above. You may also include additional comments for the author, including concerns about dual publication, research ethics, or publication ethics. (Please upload your review as an attachment if it exceeds 20,000 characters)

**Reviewer #1**:

1. Table 3 remove the t-value from the table.

Reply: Thank you for the reviewer’s comment. The t-values have been removed from Table 3. (line367)

2. Table 4; Present only the beta and a 95% CI. Remove Se, Wald chi square, df and p value.
Reply: Thank you for the reviewer’s suggestion. We have revised Table 4 to present only the β coefficients and 95% confidence intervals, and have removed the SE, Wald chi-square, degrees of freedom, and p-values accordingly. (line 414)

3. table 4 is described as interaction effects between group and time. Are you sure that is what you are presenting here? Please write out the model and show which parameters are included in the table.

Reply: Thank you for the reviewer’s comment. We confirm that the results presented in Table 4 represent the interaction effects (group × time) in the GEE analysis.

In our study, the GEE model included group, time, and the group × time interaction term as the main independent variables. The β coefficients presented in Table 4 correspond to the interaction terms, reflecting the differences in the magnitude of change before and after the intervention between each EG and the CG. A clarification has also been added below Table 4. (line 416-419)

4. How were the subjects who dropped out handled? If these are excluded then this is not an intention to treat analysis and this should be acknowledged as a potential source of bias.

Reply: Thank you for the reviewer’s comment. In this study, participants who dropped out were excluded from the final analysis; therefore, a per-protocol analysis was conducted rather than an intention-to-treat (ITT) analysis. We acknowledge that this approach may introduce attrition bias, particularly if the characteristics of participants who dropped out differ from those who completed the study, which may affect the internal validity of the results. This potential limitation has been addressed in both the Methods and Limitations sections to provide a more comprehensive explanation. (line 142 and line 564-566)

5. Please also acknowledge as a limitation the very small size of the control group, which was not stratified by length of long COVID.
Reply: Thank you for the reviewer’s comment. This study adopted a quasi-experimental design, with the primary aim of comparing intervention effects across different recovery durations; therefore, stratification based on recovery time was applied only within the EGs. The CG was used as a reference for the natural progression of symptoms without intervention and was not further stratified. We agree that the small sample size of the CG and the lack of stratification by duration of long COVID may affect the stability of between-group comparisons and the internal validity of the study, thereby influencing the interpretation of the results. This limitation has been acknowledged in the revised manuscript. (line 560-564)

**Reviewer #2**: (No Response)

**Reviewer #3**: The authors have thoroughly and satisfactorily addressed my previous concerns regarding the study design and the intervention's clinical role.
Specifically, the authors provided crucial context regarding the lack of standardized pulmonary rehabilitation programs in the study's region during the data collection period. This adequately justifies the composition of the standard care control arm and resolves my ethical and methodological concerns. Furthermore, the revised Introduction accurately positions Incentive Spirometry not as a replacement for comprehensive pulmonary rehabilitation, but as a practical, low-cost, and accessible adjunct for resource-limited settings.
These clarifications significantly strengthen the manuscript's clinical relevance and scientific integrity. I have no further concerns and recommend the manuscript for publication.

Reply: Thank you for the reviewer’s comments. We will continue to refine our work and contribute further evidence in this field in future studies.

**Reviewer #4**: Dear colleagues, thank you for giving me the opportunity to review your article. Basically, the article is of interest in the sense of post-covid rehabilitation possibilities. I had not the opportunity to review previous versions, but this version is much improved. I would have some minor suggestions to even more improve your article:

1. “Study on Post-Acute COVID-19 Syndrome in Improvement of 2 COVID-19 Rehabilitated Patients by Respiratory Training.”
The article title can be misleading and not informative enough what the article is about so I would suggest you to rephrase it.
Reply: Thank you for the reviewer’s suggestion. We agree that the original title was not sufficiently precise and did not fully reflect the focus of the study. Therefore, we have revised the title to “Effects of Incentive Spirometer Training on Dyspnea and Functional Status in Patients with Long COVID,” emphasizing the main intervention and key outcome measures (dyspnea and functional status) to improve clarity and informativeness.

2. “Currently, no definitive treatment for Long COVID.”
This is not a valid sentence, correct it.
Reply: Thank you for the reviewer’s suggestion. We have corrected the grammatical error and revised the sentence to avoid an overly absolute statement. (line 86)

3. “Respiratory training—commonly used to manage symptoms in chronic pulmonary disease—is considered a potentially beneficial intervention for Long COVID; however, supporting evidence remains limited.”
This is not truth, evidence exist so please rephrase this sentence and add new references. That should be corrected throughout the article.
Reply: Thank you for the reviewer’s comment. We agree that existing studies have supported the potential benefits of respiratory training for long COVID. Therefore, we have revised the statement to emphasize the heterogeneity of the current evidence and the lack of consistent conclusions, and have added relevant references to provide a more comprehensive and accurate description. (line87-91)
4. “provides patients with an alternative choice”
No truth because it was not compared with pulmonary rehabilitation. You should omit this sentence.
Reply: Thank you for the reviewer’s suggestion. We agree with this concern and have removed the statement accordingly. (line 111)

5. “Participants were randomly assigned to groups based on recovery duration.”
If they were assigned by recovery duration what was random? The later explanation in the article is correct, but this sentence is not.
Reply: Thank you for the reviewer’s comment. We agree that the original statement was not sufficiently precise and may have caused misunderstanding regarding the group allocation process. In this study, participants were first randomly assigned to the experimental and control groups, and the experimental group was subsequently stratified based on recovery duration. Therefore, the grouping based on recovery duration was not part of the randomization process. The relevant sentence has been revised to more clearly describe the study design. (line120 and line 258-260)

6. “The flow diagram of participant enrollment and allocation in figure 1.”
Incorrect sentence, please correct it.

Reply: Thank you for the reviewer’s comment. We have revised the sentence for clarity and accuracy. (line 126)
