# Supplementary material for: Effects of incentive spirometer training on dyspnea and functional status in patients with long COVID
Source: PLoS One. 2026 Jun 22;21(6):e0351553. doi: 10.1371/journal.pone.0351553 (PMC13286201; doi:10.1371/journal.pone.0351553)
Supplement: Peer review history

# Peer review: Decision Letter 0

**DOI:** 10.1371/journal.pone.0351553.r001

**Authors:** Toufic Ahmad Chaaban

20 Nov 2025

PONE-D-25-41209Study on Post-Acute COVID-19 Syndrome in Improvement of COVID-19 Rehabilitated Patients by Respiratory TrainingPLOS ONE

Dear Dr. Hsieh,

Thank you for submitting your manuscript to PLOS ONE. After careful consideration, we feel that it has merit but does not fully meet PLOS ONE’s publication criteria as it currently stands. Therefore, we invite you to submit a revised version of the manuscript that addresses the points raised during the review process.

Please submit your revised manuscript by Jan 02 2026 11:59PM. If you will need more time than this to complete your revisions, please reply to this message or contact the journal office at plosone@plos.org. When you're ready to submit your revision, log on to https://www.editorialmanager.com/pone/ and select the 'Submissions Needing Revision' folder to locate your manuscript file.

Please include the following items when submitting your revised manuscript:

- A rebuttal letter that responds to each point raised by the academic editor and reviewer(s). You should upload this letter as a separate file labeled 'Response to Reviewers'.
- A marked-up copy of your manuscript that highlights changes made to the original version. You should upload this as a separate file labeled 'Revised Manuscript with Track Changes'.
- An unmarked version of your revised paper without tracked changes. You should upload this as a separate file labeled 'Manuscript'.

If you would like to make changes to your financial disclosure, please include your updated statement in your cover letter. Guidelines for resubmitting your figure files are available below the reviewer comments at the end of this letter.

If applicable, we recommend that you deposit your laboratory protocols in protocols.io to enhance the reproducibility of your results. Protocols.io assigns your protocol its own identifier (DOI) so that it can be cited independently in the future. For instructions see: https://journals.plos.org/plosone/s/submission-guidelines#loc-laboratory-protocols. Additionally, PLOS ONE offers an option for publishing peer-reviewed Lab Protocol articles, which describe protocols hosted on protocols.io. Read more information on sharing protocols at https://plos.org/protocols?utm_medium=editorial-email&utm_source=authorletters&utm_campaign=protocols.

We look forward to receiving your revised manuscript.

Kind regards,

Toufic Ahmad Chaaban, M.D.

Academic Editor

PLOS ONE

Journal Requirements:

When submitting your revision, we need you to address these additional requirements.

1. Please ensure that your manuscript meets PLOS ONE's style requirements, including those for file naming. The PLOS ONE style templates can be found at

https://journals.plos.org/plosone/s/file?id=wjVg/PLOSOne_formatting_sample_main_body.pdf and

https://journals.plos.org/plosone/s/file?id=ba62/PLOSOne_formatting_sample_title_authors_affiliations.pdf

2. Thank you for stating in your Funding Statement:

“Initials of the authors who received : Yao-Hsiang Chen

Enter a statement with the following details:

Initials of the authors who received award: Conceptualization, Data collection, Formal analysis, Visualization, Investigation

This work was support by Tri-service General Hospital Songshan Branch (Grant number is TSGH-SS_D_113004).”

Please provide an amended statement that declares *all* the funding or sources of support (whether external or internal to your organization) received during this study, as detailed online in our guide for authors at http://journals.plos.org/plosone/s/submit-now. Please also include the statement “There was no additional external funding received for this study.” in your updated Funding Statement.

Please include your amended Funding Statement within your cover letter. We will change the online submission form on your behalf.

3. Thank you for stating the following financial disclosure:

“Initials of the authors who received : Yao-Hsiang Chen

Enter a statement with the following details:

Initials of the authors who received award: Conceptualization, Data collection, Formal analysis, Visualization, Investigation

This work was support by Tri-service General Hospital Songshan Branch (Grant number is TSGH-SS_D_113004).”

Please state what role the funders took in the study. If the funders had no role, please state: "The funders had no role in study design, data collection and analysis, decision to publish, or preparation of the manuscript."

If this statement is not correct you must amend it as needed.

Please include this amended Role of Funder statement in your cover letter; we will change the online submission form on your behalf.

4. Your ethics statement should only appear in the Methods section of your manuscript. If your ethics statement is written in any section besides the Methods, please move it to the Methods section and delete it from any other section. Please ensure that your ethics statement is included in your manuscript, as the ethics statement entered into the online submission form will not be published alongside your manuscript.

5. We are unable to open your Supporting Information file table 1-8.rar. Please kindly revise as necessary and re-upload.

6. Please include captions for your Supporting Information files at the end of your manuscript, and update any in-text citations to match accordingly. Please see our Supporting Information guidelines for more information: http://journals.plos.org/plosone/s/supporting-information.

7. If the reviewer comments include a recommendation to cite specific previously published works, please review and evaluate these publications to determine whether they are relevant and should be cited. There is no requirement to cite these works unless the editor has indicated otherwise.

Additional Editor Comments:

This manuscript require significant edits and revisions before deciding on publication. Please address reviewers comments and consider sharing the protocol prior to the study and if it was published

[Note: HTML markup is below. Please do not edit.]

Reviewers' comments:

Reviewer's Responses to Questions

**Comments to the Author**

1. Is the manuscript technically sound, and do the data support the conclusions?

The manuscript must describe a technically sound piece of scientific research with data that supports the conclusions. Experiments must have been conducted rigorously, with appropriate controls, replication, and sample sizes. The conclusions must be drawn appropriately based on the data presented.

Reviewer #1: Yes

Reviewer #2: Yes

Reviewer #3: Partly

**********

2. Has the statistical analysis been performed appropriately and rigorously?

Reviewer #1: No

Reviewer #2: Yes

Reviewer #3: Yes

**********

3. Have the authors made all data underlying the findings in their manuscript fully available?

The PLOS Data policy requires authors to make all data underlying the findings described in their manuscript fully available without restriction, with rare exception (please refer to the Data Availability Statement in the manuscript PDF file). The data should be provided as part of the manuscript or its supporting information, or deposited to a public repository. For example, in addition to summary statistics, the data points behind means, medians and variance measures should be available. If there are restrictions on publicly sharing data—e.g. participant privacy or use of data from a third party—those must be specified.

Reviewer #1: No

Reviewer #2: Yes

Reviewer #3: Yes

**********

4. Is the manuscript presented in an intelligible fashion and written in standard English?

PLOS ONE does not copyedit accepted manuscripts, so the language in submitted articles must be clear, correct, and unambiguous. Any typographical or grammatical errors should be corrected at revision, so please note any specific errors here.

Reviewer #1: Yes

Reviewer #2: Yes

Reviewer #3: Yes

**********

5. Review Comments to the Author

Please use the space provided to explain your answers to the questions above. You may also include additional comments for the author, including concerns about dual publication, research ethics, or publication ethics. (Please upload your review as an attachment if it exceeds 20,000 characters)

Reviewer #1: This reports the results of an RCT that compared the efficacy of IS for improving dyspnea and functional status in post COVID 19 patients. Unfortunately, this paper is impossible to read and understand.

-A ton of outcomes are presented - with no distinction about which are the primary outcomes and which are secondary.

-It is unclear how the randomization was carried out.

-The experimental group is stratified by time since infection, but it is unclear whether this was defined a priori or not.

-The analysis compares within changes from baseline to post intervention in each group. What is important is if these changes are different in the experimental arms vs. the control arm. See suggestions below.

1. How exactly was the randomization carried out? Please see the consort checklist and ensure that all the details are given, in one section. Right now these are scattered throughout the manuscript.

2. Why was such a high power used for the sample size calculation? What outcome was the sample size based on? What detectable difference was used? What SD was used? Please justify each choice.

3. Please be specific about the primary outcome. Was this 6MT walk at follow up or was it the change from baseline? OR was it dyspnea?

4. Were the groups defined a priori?

5. Please remove the p value from Table 2. Also standardize the number of decimal places. For the continuous variables present mean (SD). Also - please fix the format - it is impossible to read as is. What does a mean 1.8 for gender mean??

6. The primary analysis should compare the change in 6 minute walk between the intervention groups vs the control group overall. And then, the same thing for dyspnea. Then, use a regression model that includes arm and the group, and the interaction between arm and group - the crucial thing is if the effect of the intervention differs by group.

7. How exactly were the GEE fit? What correlation structure was used? Were robust standard errors used? Table 4 presents group by time interaction - this is a little better - now we can see that some of the groups have different changes than the control group.

8. Table 8 is extraneous, and not part of the objectives. Remove all these results.

9. the discussion says that "Among these, EG1, who underwent the intervention within 3 months of recovery, showed the greatest improvement, with a 79% reduction in D-12 scores." Is this statistically different than the changes observed in the other EGs?

Reviewer #2: This is an excellent article focusing on a highly relevant and common issue currently encountered in pulmonary and medicine clinics.

The study effectively evaluates the use of incentive spirometry as a component of respiratory training to relieve symptoms associated with long COVID. While there are currently no established treatment alternatives proven to resolve long COVID, respiratory training—including techniques like incentive spirometry—has long been utilized in the management of other chronic lung diseases. This approach aims to improve lung function, reduce dyspnea, and enhance overall functional status, suggesting that such interventions may play a valuable role in the multidisciplinary management of post-COVID patients

The study features an innovative approach by employing multiple experimental groups alongside a control group. These groups are evaluated longitudinally, according to the duration elapsed since recovery from COVID-19. This design allows for assessment of intervention effectiveness over time, providing valuable insights into how respiratory training may impact functional recovery and symptom resolution in the post-COVID population.

A key strength of the study lies in the intervention's simplicity, which is easy to implement and associated with limited to no immediate perceived harm to the patient. Moreover, the fact that incentive spirometry can be self-performed by patients adds to its practicality and feasibility, making it an attractive option for managing long COVID symptoms in the outpatient setting. The sample size for this study was appropriately calculated using statistical methods, including repeated measures one-way ANOVA. The investigators also accounted for a potential 10% dropout rate by enrolling an adequate number of additional participants. This approach enhances the robustness of the findings and ensures that sufficient data would be available for meaningful analysis despite potential attrition.

The diagnosis of long COVID is a complex and nuanced topic that warrants discussion on its own. Unlike many other conditions, long COVID remains primarily a diagnosis of exclusion, as there is currently no definitive imaging or blood-based test to confirm its presence. Clinicians must therefore rely on clinical evaluation, history, and the persistence of characteristic symptoms beyond the acute phase of infection, while actively ruling out alternative explanations. This diagnostic uncertainty presents a significant challenge in both research and clinical practice.

A significant limitation of the study is its lack of consideration for the inherent severity or risk posed by pre-existing cardiopulmonary disease. For example, the study does not account for whether participants’ initial COVID-19 illness was complicated by respiratory failure, myocarditis, hypoxemic respiratory failure, or pulmonary embolism. Instead, it includes individuals who have recovered from COVID-19 within the past year, without stratifying them according to the severity or type of their prior cardiopulmonary involvement. A mean 6-minute walk distance of 347 meters indicates that the study group consisted of a relatively healthy population with moderate baseline functional capacity. This may limit the applicability of the findings to individuals with more severe impairment or lower exercise tolerance. Subjective performance in interventions such as respiratory training may vary significantly with age, as younger patients often tolerate these exercises better than older individuals. There is no age-based stratified analysis to address these potential differences. Furthermore, the study does not fully account for underlying health risk factors such as pre-existing cardiopulmonary disease and environmental exposures, including urban air pollution. Although hypertension and cardiovascular disease were the most reported underlying medical conditions among participants, it remains uncertain whether individuals with chronic lung diseases were excluded. These factors could substantially influence both baseline functional status and response to intervention, and their omission may limit the generalizability of the study’s findings. The study comments on baseline exercise tolerance, noting that 40% of participants did not engage in regular physical activity. However, it is unclear whether this lack of activity reflects participants’ habits prior to their COVID-19 episode or is a consequence of their illness and recovery. This ambiguity makes it difficult to interpret the impact of the intervention on overall exercise capacity and recovery trajectories. Although participants of the study have at least 1 or 2 episodes of covid infection, the study does not address whether outcomes differed between individuals with repeated COVID-19 infections compared to those who experienced a single episode. This information is not provided, leaving uncertainty about whether recurrent infection may impact functional recovery or response to respiratory training in long COVID patients. Injectable antivirals were only used in 3 out of 85 participants (4% of study population). so, I would expect the participants to have a lower acuity of covid infection at base line. What is the difference among those who were treated with the medications vs no medication group (28 of 85)

Despite robust sample calculation several subgroups had a limited number of patients; detailed interpretation in some of the subgroup analyses was extremely limited.

The effect of the Chinese medicine formula on the results is not clearly addressed in the study. It is unclear whether a separate analysis was conducted for patients who did not receive the Chinese medicine formula, which raises concerns about the generalizability of the findings—particularly to populations that do not use traditional Chinese medicine. Additionally, the possible interaction between the Chinese medicine formula and the intervention being studied (such as respiratory training) remains uncertain. Without subgroup analysis, it is difficult to determine whether the observed improvements in recovery are attributable to the respiratory intervention itself, the Chinese medicine formula, or a combination of both. This limitation should be considered when interpreting the applicability of the study to broader patient populations

Quality control in the study was addressed by having a nurse perform the initial training for participants. However, subsequent performance of the intervention relied on patient self-reporting, which introduces the potential for variation in both execution and documentation of the exercise. While telephone follow-up calls were used to assess compliance, a video call could have provided a more objective assessment, allowing direct observation of patient technique without the confounding factor of patients talking during incentive spirometry sessions. Employing video-based monitoring might enhance the accuracy and consistency of performance evaluation in future studies.

The study mentions regular telephone follow-ups; however, it does not specify the exact frequency of these calls. Further clarification regarding the timing and intervals of follow-up contacts would be useful to assess the consistency of monitoring and its potential impact on patient compliance and outcomes.

There appears to be some lack of clarity regarding the final number of participants included in the analysis. The study notes that five patients were lost to follow-up, and an additional three participants, who only completed the questionnaire assessments, were excluded from the final analysis. Based on these exclusions, the total analysis group was stated as 85; however, by calculation, one might expect the final number to be 82. Clarifying the exact criteria for inclusion and ensuring consistency in reporting participant numbers is important for accurate interpretation of study results.

Smoking prevalence in Taiwan, where this study was conducted, is low at around 13% overall. This aligns with the study population, in which female smoking prevalence is reported at only 2% and male smoking at 23%. These rates reflect national averages and provide important context for interpreting study outcomes, especially when considering risk factors commonly associated with cardiopulmonary health.

In table 2

When we look at the average duration of post recovery. For experimental Group 2 it is mentioned that the average duration of post recovery is only 1.2 months, I am not sure. If this is represented adequately as experimental, group 2 should be Three to six months. It is unclear why there are numbers in the row beside Hypertension in the mean and SD, I do not think we need this is necessary for categorical variables

Based on the PCFS score, there are significant differences that are noted in the Experimental Groups. It indicates that the study population is a healthier population, and the underlying COVID infection was less severe. Without any long-term Needs for supplemental oxygen. So, I would expect that the patients would not have any diseases like COVID-induced ILD.

There is a paragraph repetition between lines 110 and 120 and this could be corrected.

Reviewer #3: I appreciate the authors' commitment to rigorously evaluating a low-cost strategy for managing Long COVID syndrome, especially given the crucial need for accessible interventions in low and middle-income countries.

However, I have a significant methodological concern regarding the composition of the standard care arm and its relationship to the intervention being tested.

My concern is two-fold:

1. Exclusion of Pulmonary Rehabilitation:

Pulmonary rehabilitation is widely recognized and increasingly accepted as a cornerstone of management for persistent respiratory and functional impairment in Long COVID. Excluding it as a component of standard care for both the control and intervention groups may create a potential ethical and scientific deficit by not providing the currently accepted optimal care to the participants.

2. Role of Incentive Spirometry:

I believe that both groups should have received a structured pulmonary rehabilitation program. The authors' chosen low-cost intervention, such as incentive spirometry, should have been evaluated as an additive or specific, targeted component in addition to comprehensive pulmonary rehabilitation, rather than a potential replacement for it.

I would appreciate a strong justification from the authors for this trial design choice, particularly on why they elected to avoid including pulmonary rehabilitation in the standard care protocol, which is critical for fully assessing the incremental benefit of their low-cost strategy.

**********

6. PLOS authors have the option to publish the peer review history of their article (what does this mean?). If published, this will include your full peer review and any attached files.

If you choose “no”, your identity will remain anonymous but your review may still be made public.

**Do you want your identity to be public for this peer review?**  For information about this choice, including consent withdrawal, please see our Privacy Policy.

Reviewer #1: No

Reviewer #2: **Yes:** Madhu Kalyan Pendurthi

Reviewer #3: **Yes:** Dapanaduwage Amila Vindana Rathnapala

**********

[NOTE: If reviewer comments were submitted as an attachment file, they will be attached to this email and accessible via the submission site. Please log into your account, locate the manuscript record, and check for the action link "View Attachments". If this link does not appear, there are no attachment files.]

To ensure your figures meet our technical requirements, please review our figure guidelines: https://journals.plos.org/plosone/s/figures

You may also use PLOS’s free figure tool, NAAS, to help you prepare publication quality figures: https://journals.plos.org/plosone/s/figures#loc-tools-for-figure-preparation.

NAAS will assess whether your figures meet our technical requirements by comparing each figure against our figure specifications.

Attachment Submitted filename: PLOS Reviews 30.10.2025.docx

# Author comment: Author response to Decision Letter 1

**DOI:** 10.1371/journal.pone.0351553.r002

26 Dec 2025

We sincerely thank the reviewers for their extensive and valuable comments and suggestions, which have greatly contributed to improving the quality and clarity of our manuscript. In response to these constructive critiques, we have carefully revised the manuscript and provided detailed, point-by-point responses in the accompanying Response to Reviewers document.

<Review Comments to the Author>

Reviewer #1: This reports the results of an RCT that compared the efficacy of IS for improving dyspnea and functional status in post COVID 19 patients. Unfortunately, this paper is impossible to read and understand.

-A ton of outcomes are presented - with no distinction about which are the primary outcomes and which are secondary.

-It is unclear how the randomization was carried out.

-The experimental group is stratified by time since infection, but it is unclear whether this was defined a priori or not.

-The analysis compares within changes from baseline to post intervention in each group. What is important is if these changes are different in the experimental arms vs. the control arm. See suggestions below.

1. How exactly was the randomization carried out? Please see the consort checklist and ensure that all the details are given, in one section. Right now these are scattered throughout the manuscript.

Reply: Thank you for the reviewer’s suggestion. Because Long COVID symptoms tend to improve naturally over time, strict randomization could result in imbalance in disease status at the time of intervention and introduce systematic bias related to spontaneous recovery. In addition, it is not feasible in clinical practice to require patients who actively seek intervention to be randomly assigned to a control group. Therefore, this study adopted a predefined stratification based on recovery duration rather than random allocation. We have added this design consideration and its rationale to the Study Design section. (line124-132)

2. Why was such a high power used for the sample size calculation? What outcome was the sample size based on? What detectable difference was used? What SD was used? Please justify each choice.

Reply: Thank you for the reviewer’s insightful comment. We adopted a power of 0.95 based on the following considerations. First, the final analysis of this trial was performed using generalized estimating equations (GEE) with five study groups and a group × time interaction term. Given the nature of Long COVID, patients’ symptoms may improve spontaneously over time, and we anticipated heterogeneous recovery trajectories across the four experimental groups (0–3, 3–6, 6–9, and 9–12 months post-recovery). Such heterogeneity can disperse the overall effect across groups and reduce the effective power for detecting the interaction effect, which is the key parameter of interest in this design. To mitigate this potential dilution of statistical power, we chose a more conservative power level (0.95) to ensure adequate sensitivity for detecting clinically meaningful differences. Second, randomized controlled trials in Long COVID populations remain limited, and there is insufficient preliminary evidence to precisely estimate the true effect size of incentive spirometry in this population. Under circumstances in which the expected effect size is uncertain, using a higher power helps reduce the risk of type II error, preventing the study from overlooking a potentially meaningful intervention effect. In addition, several prior studies in similar rehabilitation contexts have also adopted a high statistical power (e.g., Power = 0.95) to ensure that clinically meaningful intervention effects can be reliably detected.( Çetin, Ş., Çetin, M., Kaplan, A., Kaplan, Ö., & Çelik, İ. (2024). The Effect of Respiratory Exercises on People with Ongoing Dyspnea and Recovered from COVID-19. EJMA, 4(1), 13-21. https://doi.org/10.14744/ejma.2023.29491.)

For these reasons, adopting a power of 0.95 was deemed an appropriate and conservative strategy for a multi-group design requiring interaction analyses.

The sample size calculation in this study was based on the primary outcome, the Dyspnea-12 (D-12) score. In studies involving Long COVID–related dyspnea, the variability of the D-12 measure generally falls within a moderate range, with standard deviations commonly reported between 5 and 7 points, which represents a typical variation observed in the literature. (Ekström, M. P., Bornefalk, H., Sköld, C. M., Janson, C., Blomberg, A., Bornefalk-Hermansson, A., Igelström, H., Sandberg, J., & Sundh, J. (2020). Minimal Clinically Important Differences and Feasibility of Dyspnea-12 and the Multidimensional Dyspnea Profile in Cardiorespiratory Disease. Journal of pain and symptom management, 60(5), 968–975.e1. https://doi.org/10.1016/j.jpainsymman.2020.05.028).The G*Power parameters adopted in this study are consistent with those later reported in similar respiratory training intervention research. For example, Çetin et al. (2024) employed sample size estimation settings comparable to those used in our study.

3. Please be specific about the primary outcome. Was this 6MT walk at follow up or was it the change from baseline? OR was it dyspnea?

Reply: Thank you for the reviewer’s insightful comment. The primary outcomes of this study were D-12 (Dyspnea-12), PCFS (Post-COVID Functional Status), and CaO₂ (arterial oxygen content). These measures were selected as the primary endpoints because Long COVID is characterized mainly by dyspnea, post-exertional discomfort, and functional limitations. D-12 and PCFS directly assess symptom burden and functional impairment, while CaO₂ reflects physiological oxygenation status. Together, these indicators more accurately capture the direct effects of the respiratory intervention on patients’ respiratory symptoms and functional recovery.

In contrast, the 6-minute walk distance (6MWD) was designated as a secondary outcome. Although 6MWD is widely used to evaluate overall cardiopulmonary endurance, it is an indirect functional measure and can be influenced by multiple non-respiratory factors. For instance, during the early phases of the pandemic in Taiwan, confirmed COVID-19 cases were required to undergo home isolation or stay in designated quarantine facilities. This markedly reduced physical activity levels and led to muscle deconditioning in many individuals. Consequently, lower 6MWD values in some participants may reflect reduced muscle strength or deconditioning rather than impaired cardiopulmonary function alone. Therefore, 6MWD was considered more suitable as a complementary functional measure rather than a primary endpoint.

Accordingly, the primary outcomes in this study were D-12, PCFS, and CaO₂, while 6MWD served as a secondary outcome assessing overall exercise tolerance. (line143-153)

4. Were the groups defined a priori?

Reply: Thank you for the reviewer’s comment. The grouping strategy in this study was defined a priori, prior to the initiation of data collection. Because one of the objectives of the study was to compare whether the effectiveness of incentive spirometry differed across varying recovery durations, the groups were predetermined during the study design phase based on the principle of Long COVID symptom duration stratification. Accordingly, the recovery periods were divided into four stages, rather than being created post hoc after data collection. We have clarified the grouping rationale and criteria in the Methods section. (line 158-167)

5. Please remove the p value from Table 2. Also standardize the number of decimal places. For the continuous variables present mean (SD). Also - please fix the format - it is impossible to read as is. What does a mean 1.8 for gender mean??

Reply: Thank you for the reviewer’s suggestion. Table 2 has been revised accordingly.

6. The primary analysis should compare the change in 6 minute walk between the intervention groups vs the control group overall. And then, the same thing for dyspnea. Then, use a regression model that includes arm and the group, and the interaction between arm and group - the crucial thing is if the effect of the intervention differs by group.

Reply: Thank you for the reviewer’s suggestion. We have revised the footnotes of Table 3 to clearly explain the meaning of the p-values and P-values, allowing readers to directly interpret the pre–post changes in 6MWD and dyspnea measures for each group, as well as the statistical significance of these changes.

In addition, we thank the reviewer for this valuable suggestion. While our study was not originally designed to test a predictive hypothesis (e.g., that a longer duration of triflow training would result in greater improvement), we understand the rationale for exploring such an analysis. Accordingly, we conducted an exploratory regression model including arm, group, and their interaction. However, the results did not show a clear or robust interaction effect.

7. How exactly were the GEE fit? What correlation structure was used? Were robust standard errors used? Table 4 presents group by time interaction - this is a little better - now we can see that some of the groups have different changes than the control group.

Reply: Thanks to the reviewer for the insightful comments. The GEE model in this study was constructed using a repeated-measures framework to evaluate changes before and after the intervention across the five study groups, as well as the group × time interaction. An autoregressive correlation structure of order 1 (AR(1)) was applied, and robust standard errors were used to ensure stable parameter estimation even if the working correlation structure was not perfectly specified.

8. Table 8 is extraneous, and not part of the objectives. Remove all these results.

Reply: Thank you for the reviewer’s suggestion. Table 8 has been removed from the main text and placed in the Appendix as supplementary table 1. (line 409)

9. the discussion says that "Among these, EG1, who underwent the intervention within 3 months of recovery, showed the greatest improvement, with a 79% reduction in D-12 scores." Is this statistically different than the changes observed in the other EGs?

Reply: Thank you for the reviewer’s comment. The improvement in D-12 was calculated as (post − pre) / pre × 100%, and the corresponding values are presented in Table 3. EG1 showed a 79% reduction, whereas EG2, EG3, and EG4 showed reductions of 59%, 62%, and 57%, respectively, indicating that EG1 demonstrated the greatest relative improvement among the experimental groups. We have referenced Table 3 in the main text for readers’ convenience. (line 418)

Reviewer #2: This is an excellent article focusing on a highly relevant and common issue currently encountered in pulmonary and medicine clinics.

The study effectively evaluates the use of incentive spirometry as a component of respiratory training to relieve symptoms associated with long COVID. While there are currently no established treatment alternatives proven to resolve long COVID, respiratory training—including techniques like incentive spirometry—has long been utilized in the management of other chronic lung diseases. This approach aims to improve lung function, reduce dyspnea, and enhance overall functional status, suggesting that such interventions may play a valuable role in the multidisciplinary management of post-COVID patients

The study features an innovative approach by employing multiple experimental groups alongside a control group. These groups are evaluated longitudinally, according to the duration elapsed since recovery from COVID-19. This design allows for assessment of intervention effectiveness over time, providing valuable insights into how respiratory training may impact functional recovery and symptom resolution in the post-COVID population.

A key strength of the study lies in the intervention's simplicity, which is easy to implement and associated with limited to no immediate perceived harm to the patient. Moreover, the fact that incentive spirometry can be self-performed by patients adds to its practicality and feasibility, making it an attractive option for managing long COVID symptoms in the outpatient setting. The sample size for this study was appropriately calculated using statistical methods, including repeated measures one-way ANOVA. The investigators also accounted for a potential 10% dropout rate by enrolling an adequate number of additional participants. This approach enhances the robustness of the findings and ensures that sufficient data would be available for meaningful analysis despite potential attrition.

The diagnosis of long COVID is a complex and nuanced topic that warrants discussion on its own. Unlike many other conditions, long COVID remains primarily a diagnosis of exclusion, as there is currently no definitive imaging or blood-based test to confirm its presence. Clinicians must therefore rely on clinical evaluation, history, and the persistence of characteristic symptoms beyond the acute phase of infection, while actively ruling out alternative explanations. This diagnostic uncertainty presents a significant challenge in both research and clinical practice.

A significant limitation of the study is its lack of consideration for the inherent severity or risk posed by pre-existing cardiopulmonary disease. For example, the study does not account for whether participants’ initial COVID-19 illness was complicated by respiratory failure, myocarditis, hypoxemic respiratory failure, or pulmonary embolism. Instead, it includes individuals who have recovered from COVID-19 within the past year, without stratifying them according to the severity or type of their prior cardiopulmonary involvement. A mean 6-minute walk distance of 347 meters indicates that the study group consisted of a relatively healthy population with moderate baseline functional capacity. This may limit the applicability of the findings to individuals with more severe impairment or lower exercise tolerance. Subjective performance in interventions such as respiratory training may vary significantly with age, as younger patients often tolerate these exercises better than older individuals. There is no age-based stratified analysis to address these potential differences. Furthermore, the study does not fully account for underlying health risk factors such as pre-existing cardiopulmonary disease and environmental exposures, including urban air pollution. Although hypertension and cardiovascular disease were the most reported underlying medical conditions among participants, it remains uncertain whether individuals with chronic lung diseases were excluded. These factors could substantially influence both baseline functional status and response to intervention, and their omission may limit the generalizability of the study’s findings. The study comments on baseline exercise tolerance, noting that 40% of participants did not engage in regular physical activity. However, it is unclear whether this lack of activity reflects participants’ habits prior to their COVID-19 episode or is a consequence of their illness and recovery. This ambiguity makes it difficult to interpret the impact of the intervention on overall exercise capacity and recovery trajectories. Although participants of the study have at least 1 or 2 episodes of covid infection, the study does not address whether outcomes differed between individuals with repeated COVID-19 infections compared to those who experienced a single episode. This information is not provided, leaving uncertainty about whether recurrent infection may impact functional recovery or response to respiratory training in long COVID patients. Injectable antivirals were only used in 3 out of 85 participants (4% of study population). so, I would expect the participants to have a lower acuity of covid infection at base line. What is the difference among those who were treated with the medications vs no medication group (28 of 85)

Reply: Thank you for the reviewer’s valuable comments. This study has indeed presented the major cardiopulmonary comorbidities in Table 1, and chronic obstructive pulmonary disease (COPD) was excluded based on the predefined inclusion and exclusion criteria. However, certain comorbidities that could not

Attachment Submitted filename: Response to Reviewers.docx

# Peer review: Decision Letter 1

**DOI:** 10.1371/journal.pone.0351553.r003

**Authors:** Toufic Ahmad Chaaban

28 Jan 2026

PONE-D-25-41209R1Study on Post-Acute COVID-19 Syndrome in Improvement of COVID-19 Rehabilitated Patients by Respiratory TrainingPLOS One

Dear Dr. Hsieh,

Thank you for submitting your manuscript to PLOS ONE. After careful consideration, we feel that it has merit but does not fully meet PLOS ONE’s publication criteria as it currently stands. Therefore, we invite you to submit a revised version of the manuscript that addresses the points raised during the review process.

Please address some residual minor comments related to structuring and results presentation

Please submit your revised manuscript by Mar 14 2026 11:59PM. If you will need more time than this to complete your revisions, please reply to this message or contact the journal office at plosone@plos.org. When you're ready to submit your revision, log on to https://www.editorialmanager.com/pone/ and select the 'Submissions Needing Revision' folder to locate your manuscript file.

Please include the following items when submitting your revised manuscript:

- A letter that responds to each point raised by the academic editor and reviewer(s). You should upload this letter as a separate file labeled 'Response to Reviewers'.
- A marked-up copy of your manuscript that highlights changes made to the original version. You should upload this as a separate file labeled 'Revised Manuscript with Track Changes'.
- An unmarked version of your revised paper without tracked changes. You should upload this as a separate file labeled 'Manuscript'.

If you would like to make changes to your financial disclosure, please include your updated statement in your cover letter. Guidelines for resubmitting your figure files are available below the reviewer comments at the end of this letter.

If applicable, we recommend that you deposit your laboratory protocols in protocols.io to enhance the reproducibility of your results. Protocols.io assigns your protocol its own identifier (DOI) so that it can be cited independently in the future. For instructions see: https://journals.plos.org/plosone/s/submission-guidelines#loc-laboratory-protocols. Additionally, PLOS ONE offers an option for publishing peer-reviewed Lab Protocol articles, which describe protocols hosted on protocols.io. Read more information on sharing protocols at https://plos.org/protocols?utm_medium=editorial-email&utm_source=authorletters&utm_campaign=protocols.

We look forward to receiving your revised manuscript.

Kind regards,

Toufic Ahmad Chaaban, M.D.

Academic Editor

PLOS One

Journal Requirements:

If the reviewer comments include a recommendation to cite specific previously published works, please review and evaluate these publications to determine whether they are relevant and should be cited. There is no requirement to cite these works unless the editor has indicated otherwise.

Please review your reference list to ensure that it is complete and correct. If you have cited papers that have been retracted, please include the rationale for doing so in the manuscript text, or remove these references and replace them with relevant current references. Any changes to the reference list should be mentioned in the rebuttal letter that accompanies your revised manuscript. If you need to cite a retracted article, indicate the article’s retracted status in the References list and also include a citation and full reference for the retraction notice.

[Note: HTML markup is below. Please do not edit.]

Reviewers' comments:

Reviewer's Responses to Questions

**Comments to the Author**

1. If the authors have adequately addressed your comments raised in a previous round of review and you feel that this manuscript is now acceptable for publication, you may indicate that here to bypass the “Comments to the Author” section, enter your conflict of interest statement in the “Confidential to Editor” section, and submit your "Accept" recommendation.

Reviewer #1: (No Response)

Reviewer #2: All comments have been addressed

Reviewer #3: All comments have been addressed

**********

2. Is the manuscript technically sound, and do the data support the conclusions?

The manuscript must describe a technically sound piece of scientific research with data that supports the conclusions. Experiments must have been conducted rigorously, with appropriate controls, replication, and sample sizes. The conclusions must be drawn appropriately based on the data presented.

Reviewer #1: No

Reviewer #2: Yes

Reviewer #3: Yes

**********

3. Has the statistical analysis been performed appropriately and rigorously?

Reviewer #1: No

Reviewer #2: Yes

Reviewer #3: N/A

**********

4. Have the authors made all data underlying the findings in their manuscript fully available?

The PLOS Data policy requires authors to make all data underlying the findings described in their manuscript fully available without restriction, with rare exception (please refer to the Data Availability Statement in the manuscript PDF file). The data should be provided as part of the manuscript or its supporting information, or deposited to a public repository. For example, in addition to summary statistics, the data points behind means, medians and variance measures should be available. If there are restrictions on publicly sharing data—e.g. participant privacy or use of data from a third party—those must be specified.

Reviewer #1: No

Reviewer #2: Yes

Reviewer #3: Yes

**********

5. Is the manuscript presented in an intelligible fashion and written in standard English?

PLOS ONE does not copyedit accepted manuscripts, so the language in submitted articles must be clear, correct, and unambiguous. Any typographical or grammatical errors should be corrected at revision, so please note any specific errors here.

Reviewer #1: Yes

Reviewer #2: Yes

Reviewer #3: Yes

**********

6. Review Comments to the Author

Please use the space provided to explain your answers to the questions above. You may also include additional comments for the author, including concerns about dual publication, research ethics, or publication ethics. (Please upload your review as an attachment if it exceeds 20,000 characters)

Reviewer #1: The paper is more understandable now.

1. Why wasn't the control group stratified on the basis of long covid? How long had the control group been suffering from long covid? How could this have affected the results?

2. The tables are now in separate documents - this is hard to review.

4. Table 4-7 - these tables are very hard to understand, and interpret. But I am happy to see the p for interaction. Can you please instead integrate the p for interaction into Table 3.

5. Also - re-arrange table 3 into Primary and Secondary outcomes.

6. Please integrate the sample size information into the main manuscript. I cannot replicate the sample size calculation - thus more details are needed. With two groups you would need a much larger sample size - I am not clear how you get to 90 total subjects.

7. the discussion says that "Among these, EG1, who underwent the intervention within 3 months of recovery, showed the greatest improvement, with a 79% reduction in D-12 scores." Is this statistically different than the changes observed in the other EGs? Your reply did not address my question.

8. The discussion emphasizes the wrong thing. The first paragraph should talk about whether D-12 change from baseline to follow up was different in the control group to the intervention group.  Within group différences could have occurrence due to usual improvement of symptoms over time.

9. The second paragraph of discussion: " Moreover, many participants reported noticeable relief from dyspnea within 3 weeks of initiating IS training, along with enhanced subjective comfort and adherence." Where are these results presented? Similarly for results pertaining to the inspiratory ball count?

10. The interpretations of the effects in lines 357-389 should emphasize the difference of differences vs the control group.

11. Everywhere - the order of results should first describe the primary results.

12. For the between group differences - should these be adjusted for relevant confounders since the EG groups are not randomized?

13. lines 339-40 "indicating that participants in EGs improved their functional status to a comparable functional level as the CG." This is an incorrect interpretation. An equivalence test would need to be conducted to make this statement.

Reviewer #2: Replies from the authors suffice the questions raised in the review. I appreciate your effort in revising several key areas of the draft.

Reviewer #3: I have reviewed the revised manuscript and the authors' response to my previous concerns regarding the study design and the standard of care provided to the control group. I appreciate the authors' thorough engagement with these points.

1. Regarding the Standard Care Arm and Pulmonary Rehabilitation: I accept the authors' explanation that at the time of the study, a standardized pulmonary rehabilitation program for Long COVID was not routinely available in clinical practice in Taiwan. This context satisfactorily justifies why pulmonary rehabilitation could not be included as the "standard of care" for the control group. It resolves the ethical and scientific concern regarding the withholding of optimal care, as the study design reflected the real-world clinical reality of that specific period and region.

2. Regarding the Role of Incentive Spirometry (IS): I commend the authors for revising the Introduction to clarify the positioning of the intervention. The added text explicitly states that "although IS cannot replace pulmonary rehabilitation, it may serve as a low-cost, self-administered intervention option... to address existing gaps". This successfully shifts the framing of the study from proposing IS as a substitute for comprehensive rehab to evaluating it as a feasible, accessible alternative for resource-limited settings.

General Observations: The inclusion of Generalized Estimating Equations (GEE) analysis and the expanded Limitations section—addressing comorbidities and pre-existing conditions —have significantly strengthened the rigor of the manuscript. The revisions have adequately addressed my methodological concerns.

I have no further comments and recommend the manuscript for publication.

**********

7. PLOS authors have the option to publish the peer review history of their article (what does this mean?). If published, this will include your full peer review and any attached files.

If you choose “no”, your identity will remain anonymous but your review may still be made public.

**Do you want your identity to be public for this peer review?**  For information about this choice, including consent withdrawal, please see our Privacy Policy.

Reviewer #1: No

Reviewer #2: **Yes:** Madhu Kalyan Pendurthi

Reviewer #3: **Yes:** Dapanaduwage Amila Vindana Rathnapala

**********

[NOTE: If reviewer comments were submitted as an attachment file, they will be attached to this email and accessible via the submission site. Please log into your account, locate the manuscript record, and check for the action link "View Attachments". If this link does not appear, there are no attachment files.]

To ensure your figures meet our technical requirements, please review our figure guidelines: https://journals.plos.org/plosone/s/figures

You may also use PLOS’s free figure tool, NAAS, to help you prepare publication quality figures: https://journals.plos.org/plosone/s/figures#loc-tools-for-figure-preparation.

NAAS will assess whether your figures meet our technical requirements by comparing each figure against our figure specifications.

# Author comment: Author response to Decision Letter 2

**DOI:** 10.1371/journal.pone.0351553.r004

21 Feb 2026

Reviewer #1: The paper is more understandable now.

1. Why wasn't the control group stratified on the basis of long covid? How long had the control group been suffering from long covid? How could this have affected the results?

Reply:

Thank you for the reviewer’s suggestion. This study employed a quasi-experimental design rather than a fully randomized controlled trial. The primary objective was to compare the effectiveness of incentive spirometry initiated at different recovery time points; therefore, stratification based on recovery duration from Long COVID was applied only within the experimental groups. The control group was designed to observe the natural course of symptom changes over time without intervention, and thus was not further stratified according to the duration of Long COVID.

The duration from COVID-19 infection to enrollment for participants in the control group has been presented in Table 2 for reference.

We acknowledge that the absence of stratification in the control group may have introduced a degree of within-group heterogeneity. However, as recruitment occurred during the later phase of the pandemic, the distribution of Long COVID cases was inherently uneven. Imposing additional stratification criteria might have further reduced the sample size and compromised the feasibility of statistical analyses. Nevertheless, baseline comparisons revealed no significant differences among groups. Therefore, we believe that this design limitation is unlikely to have materially affected the main conclusions. Future larger-scale studies may consider stratified designs to further enhance internal validity. We appreciate the reviewer’s insightful suggestion.

2. The tables are now in separate documents - this is hard to review.

Reply:Thank you for the reviewer’s suggestion. We have now reintegrated all tables into the main manuscript to facilitate review and improve readability.

4. Table 4-7 - these tables are very hard to understand, and interpret. But I am happy to see the p for interaction. Can you please instead integrate the p for interaction into Table 3.

Reply:

Thank you for the reviewer’s valuable suggestion. Original Table 3 presents the between-group comparisons as well as the within-group pre–post differences and their corresponding p-values. In contrast, Tables 4–7 report the results of the generalized estimating equations (GEE) analysis examining the time × group interaction. As these analyses are based on different statistical methods and analytical frameworks, they were not combined into a single table.

However, we acknowledge that presenting Tables 4–7 separately may have affected the readability and clarity of the results. After considering the reviewer’s suggestion, we have consolidated the GEE results from Tables 4–7 into a single table and reorganized them according to primary and secondary outcomes to improve overall clarity and interpretability.

5. Also - re-arrange table 3 into Primary and Secondary outcomes.

Reply:

Thank you for the reviewer’s suggestion. The order of presentation in Table 3 has been revised to distinguish between primary and secondary outcomes.

6. Please integrate the sample size information into the main manuscript. I cannot replicate the sample size calculation - thus more details are needed. With two groups you would need a much larger sample size - I am not clear how you get to 90 total subjects.

Reply:

Thank you for the reviewer’s comment. The sample size estimation in this study was not based on a two-group comparison. Instead, it was calculated according to a five-group design (four experimental groups and one control group) using a repeated-measures within–between interaction framework, with all groups analyzed concurrently. The control group received no intervention. We have clearly specified this design in the Methods section (Line 202-208) to avoid misunderstanding that the study was structured as a two-group comparison trial.

7. the discussion says that "Among these, EG1, who underwent the intervention within 3 months of recovery, showed the greatest improvement, with a 79% reduction in D-12 scores." Is this statistically different than the changes observed in the other EGs? Your reply did not address my question.

Reply:Thank you for the reviewer’s further clarification. Although EG1 demonstrated the highest percentage reduction in D-12 scores, EG4 showed a relatively larger absolute mean difference between pre- and post-intervention measurements. This may be attributable to more severe baseline symptoms in EG4, resulting in greater room for improvement. However, to avoid overinterpretation, we have revised the wording in the Discussion. The original statement emphasizing that EG1 showed the “greatest” improvement has been removed and replaced with a statement indicating that both EG1 and EG4 demonstrated statistically significant improvements in the GEE analysis (Line 451-453), in order to more accurately reflect the statistical findings.

8. The discussion emphasizes the wrong thing. The first paragraph should talk about whether D-12 change from baseline to follow up was different in the control group to the intervention group. Within group différences could have occurrence due to usual improvement of symptoms over time.

Reply:

1. Thank you for the reviewer’s important comment. We have revised the first paragraph of the Discussion to explicitly emphasize the difference in change between the EGs and the CG, rather than focusing solely on within-group improvements. (line 436-439)

2. We fully agree that within-group improvement may partly reflect the natural resolution of symptoms over time. Therefore, this potential effect was considered at both the study design and statistical analysis stages. In this study, GEE were used to examine the time × group interaction, allowing us to assess whether the intervention effect was significantly greater than the natural change observed in the CG, thereby minimizing the influence of time effects alone.

9. The second paragraph of discussion: " Moreover, many participants reported noticeable relief from dyspnea within 3 weeks of initiating IS training, along with enhanced subjective comfort and adherence." Where are these results presented? Similarly for results pertaining to the inspiratory ball count?

Reply:

Thank you for the reviewer’s comment. Regarding the statement that “many participants reported noticeable relief from dyspnea within 3 weeks of initiating IS training, along with enhanced subjective comfort and adherence,” we have clarified in the revised manuscript that this information was obtained during the mid-intervention telephone follow-up conducted at week 3. During this follow-up, participants provided verbal reports regarding changes in their symptoms and training experiences. (line454-456)

As for the inspiratory ball count, its primary purpose was to serve as a training reference during the intervention, helping participants monitor their inspiratory effort. The corresponding data are presented in Table 3. However, because this measure represents only a semi-quantitative visual feedback provided by the incentive spirometer and does not precisely reflect actual inspiratory volume, and because it was not predefined as a primary or secondary outcome in the study design, it was not described as a formal efficacy outcome in the Results section. Instead, it is presented as supplementary information for reference.

10. The interpretations of the effects in lines 357-389 should emphasize the difference of differences vs the control group.

Reply:

Thank you for the reviewer’s comment. We have revised the interpretation accordingly, shifting the emphasis from within-group pre–post changes to highlighting the difference-in-differences between the EGs and the CG.

11. Everywhere - the order of results should first describe the primary results.

Reply:T

hank you for the reviewer’s comment. We have reorganized the Results section to present the primary outcomes first, followed by the secondary outcomes, in order to improve clarity and better align with the study design.

12. For the between group differences - should these be adjusted for relevant confounders since the EG groups are not randomized?

Reply:Thank you for the reviewer’s comment. We agree with the reviewer’s perspective. Although randomization was not performed, we made efforts to ensure comparability at baseline, and no significant differences were observed among groups in baseline characteristics (as presented in Table 2). In addition, the GEE approach accounts for within-subject correlations and time effects, which partially addresses potential confounding related to repeated measurements. Therefore, no additional covariate adjustments were included in the primary analysis. We appreciate this suggestion and will take it into consideration in future larger-scale studies.

13. lines 339-40 "indicating that participants in EGs improved their functional status to a comparable functional level as the CG." This is an incorrect interpretation. An equivalence test would need to be conducted to make this statement.

Reply:

Thank you for the reviewer’s correction. We agree that this statement may constitute an overinterpretation, and that the term “comparable” should not be used without conducting an equivalence test. Therefore, we have removed this sentence from the main text to avoid potential misunderstanding.

Reviewer #2: Replies from the authors suffice the questions raised in the review. I appreciate your effort in revising several key areas of the draft.

Reply:

Thank you very much for the reviewer’s recognition. We sincerely appreciate your thoughtful comments and encouragement. We will continue to refine our work in this field and consider conducting larger-scale studies in the future to further strengthen the evidence base.

Reviewer #3: I have reviewed the revised manuscript and the authors' response to my previous concerns regarding the study design and the standard of care provided to the control group. I appreciate the authors' thorough engagement with these points.

1. Regarding the Standard Care Arm and Pulmonary Rehabilitation: I accept the authors' explanation that at the time of the study, a standardized pulmonary rehabilitation program for Long COVID was not routinely available in clinical practice in Taiwan. This context satisfactorily justifies why pulmonary rehabilitation could not be included as the "standard of care" for the control group. It resolves the ethical and scientific concern regarding the withholding of optimal care, as the study design reflected the real-world clinical reality of that specific period and region.

2. Regarding the Role of Incentive Spirometry (IS): I commend the authors for revising the Introduction to clarify the positioning of the intervention. The added text explicitly states that "although IS cannot replace pulmonary rehabilitation, it may serve as a low-cost, self-administered intervention option... to address existing gaps". This successfully shifts the framing of the study from proposing IS as a substitute for comprehensive rehab to evaluating it as a feasible, accessible alternative for resource-limited settings.

General Observations: The inclusion of Generalized Estimating Equations (GEE) analysis and the expanded Limitations section—addressing comorbidities and pre-existing conditions —have significantly strengthened the rigor of the manuscript. The revisions have adequately addressed my methodological concerns.

I have no further comments and recommend the manuscript for publication.

Reply:

Thank you very much for the reviewer’s thoughtful comments and encouragement. We truly appreciate your support, and will continue to refine our work and aim to conduct larger-scale studies in the future to further strengthen the evidence in this field.

Attachment Submitted filename: Response to Reviewers-0217.pdf

# Peer review: Decision Letter 2

**DOI:** 10.1371/journal.pone.0351553.r005

**Authors:** Toufic Ahmad Chaaban

10 Apr 2026

PONE-D-25-41209R2Study on Post-Acute COVID-19 Syndrome in Improvement of COVID-19 Rehabilitated Patients by Respiratory TrainingPLOS One

Dear Dr. Hsieh,

Thank you for submitting your manuscript to PLOS ONE. After careful consideration, we feel that it has merit but does not fully meet PLOS ONE’s publication criteria as it currently stands. Therefore, we invite you to submit a revised version of the manuscript that addresses the points raised during the review process.

==============================

**ACADEMIC EDITOR:** please make corrections according to reviewers' comments or write a detailed rebuttal on a point-by-point basis.

==============================

Please submit your revised manuscript by May 25 2026 11:59PM. If you will need more time than this to complete your revisions, please reply to this message or contact the journal office at plosone@plos.org. When you're ready to submit your revision, log on to https://www.editorialmanager.com/pone/ and select the 'Submissions Needing Revision' folder to locate your manuscript file.

Please include the following items when submitting your revised manuscript:

- A letter that responds to each point raised by the academic editor and reviewer(s). You should upload this letter as a separate file labeled 'Response to Reviewers'.
- A marked-up copy of your manuscript that highlights changes made to the original version. You should upload this as a separate file labeled 'Revised Manuscript with Track Changes'.
- An unmarked version of your revised paper without tracked changes. You should upload this as a separate file labeled 'Manuscript'.

If you would like to make changes to your financial disclosure, please include your updated statement in your cover letter. Guidelines for resubmitting your figure files are available below the reviewer comments at the end of this letter.

If applicable, we recommend that you deposit your laboratory protocols in protocols.io to enhance the reproducibility of your results. Protocols.io assigns your protocol its own identifier (DOI) so that it can be cited independently in the future. For instructions see: https://journals.plos.org/plosone/s/submission-guidelines#loc-laboratory-protocols. Additionally, PLOS ONE offers an option for publishing peer-reviewed Lab Protocol articles, which describe protocols hosted on protocols.io. Read more information on sharing protocols at https://plos.org/protocols?utm_medium=editorial-email&utm_source=authorletters&utm_campaign=protocols.

As the corresponding author, your ORCID iD is verified in the submission system and will appear in the published article. PLOS supports the use of ORCID, and we encourage all coauthors to register for an ORCID iD and use it as well. Please encourage your coauthors to verify their ORCID iD within the submission system before final acceptance, as unverified ORCID iDs will not appear in the published article. *Only*  the individual author can complete the verification step; PLOS staff *cannot*  verify ORCID iDs on behalf of authors.

We look forward to receiving your revised manuscript.

Kind regards,

Davor Plavec, MD, MSc, PhD, Prof.

Academic Editor

PLOS One

Journal Requirements:

If the reviewer comments include a recommendation to cite specific previously published works, please review and evaluate these publications to determine whether they are relevant and should be cited. There is no requirement to cite these works unless the editor has indicated otherwise.

Please review your reference list to ensure that it is complete and correct. If you have cited papers that have been retracted, please include the rationale for doing so in the manuscript text, or remove these references and replace them with relevant current references. Any changes to the reference list should be mentioned in the rebuttal letter that accompanies your revised manuscript. If you need to cite a retracted article, indicate the article’s retracted status in the References list and also include a citation and full reference for the retraction notice.

Additional Editor Comments:

Dear Authors,

please make corrections according to reviewers' comments or write a detailed rebuttal on a point-by-point basis.

[Note: HTML markup is below. Please do not edit.]

Reviewers' comments:

Reviewer's Responses to Questions

**Comments to the Author**

1. If the authors have adequately addressed your comments raised in a previous round of review and you feel that this manuscript is now acceptable for publication, you may indicate that here to bypass the “Comments to the Author” section, enter your conflict of interest statement in the “Confidential to Editor” section, and submit your "Accept" recommendation.

Reviewer #1: (No Response)

Reviewer #2: All comments have been addressed

Reviewer #3: All comments have been addressed

Reviewer #4: (No Response)

**********

2. Is the manuscript technically sound, and do the data support the conclusions?

The manuscript must describe a technically sound piece of scientific research with data that supports the conclusions. Experiments must have been conducted rigorously, with appropriate controls, replication, and sample sizes. The conclusions must be drawn appropriately based on the data presented.

Reviewer #1: Partly

Reviewer #2: Yes

Reviewer #3: Yes

Reviewer #4: Yes

**********

3. Has the statistical analysis been performed appropriately and rigorously?

Reviewer #1: Yes

Reviewer #2: Yes

Reviewer #3: N/A

Reviewer #4: I Don't Know

**********

4. Have the authors made all data underlying the findings in their manuscript fully available?

The PLOS Data policy requires authors to make all data underlying the findings described in their manuscript fully available without restriction, with rare exception (please refer to the Data Availability Statement in the manuscript PDF file). The data should be provided as part of the manuscript or its supporting information, or deposited to a public repository. For example, in addition to summary statistics, the data points behind means, medians and variance measures should be available. If there are restrictions on publicly sharing data—e.g. participant privacy or use of data from a third party—those must be specified.

Reviewer #1: No

Reviewer #2: Yes

Reviewer #3: Yes

Reviewer #4: Yes

**********

5. Is the manuscript presented in an intelligible fashion and written in standard English?

PLOS ONE does not copyedit accepted manuscripts, so the language in submitted articles must be clear, correct, and unambiguous. Any typographical or grammatical errors should be corrected at revision, so please note any specific errors here.

Reviewer #1: Yes

Reviewer #2: Yes

Reviewer #3: Yes

Reviewer #4: Yes

**********

6. Review Comments to the Author

Please use the space provided to explain your answers to the questions above. You may also include additional comments for the author, including concerns about dual publication, research ethics, or publication ethics. (Please upload your review as an attachment if it exceeds 20,000 characters)

Reviewer #1: 1. Table 3 remove the t-value from the table.

2. Table 4; Present only the beta and a 95% CI. Remove Se, Wald chi square, df and p value.

3. table 4 is described as interaction effects between group and time. Are you sure that is what you are presenting here? Please write out the model and show which parameters are included in the table.

4. How were the subjects who dropped out handled? If these are excluded then this is not an intention to treat analysis and this should be acknowledged as a potential source of bias.

5. Please also acknowledge as a limitation the very small size of the control group, which was not stratified by length of long COVID.

Reviewer #2: (No Response)

Reviewer #3: The authors have thoroughly and satisfactorily addressed my previous concerns regarding the study design and the intervention's clinical role.

Specifically, the authors provided crucial context regarding the lack of standardized pulmonary rehabilitation programs in the study's region during the data collection period. This adequately justifies the composition of the standard care control arm and resolves my ethical and methodological concerns. Furthermore, the revised Introduction accurately positions Incentive Spirometry not as a replacement for comprehensive pulmonary rehabilitation, but as a practical, low-cost, and accessible adjunct for resource-limited settings.

These clarifications significantly strengthen the manuscript's clinical relevance and scientific integrity. I have no further concerns and recommend the manuscript for publication.

Reviewer #4: Dear colleagues, thank you for giving me the opportunity to review your article. Basically, the article is of interest in the sense of post-covid rehabilitation possibilities. I had not the opportunity to review previous versions, but this version is much improved. I would have some minor suggestions to even more improve your article:

1. “Study on Post-Acute COVID-19 Syndrome in Improvement of 2 COVID-19 Rehabilitated Patients by Respiratory Training.”

The article title can be misleading and not informative enough what the article is about so I would suggest you to rephrase it.

2. “Currently, no definitive treatment for Long COVID.”

This is not a valid sentence, correct it.

3. “Respiratory training—commonly used to manage symptoms in chronic pulmonary disease—is considered a potentially beneficial intervention for Long COVID; however, supporting evidence remains limited.”

This is not truth, evidence exist so please rephrase this sentence and add new references. That should be corrected throughout the article.

4. “provides patients with an alternative choice”

No truth because it was not compared with pulmonary rehabilitation. You should omit this sentence.

5. “Participants were randomly assigned to groups based on recovery duration.”

If they were assigned by recovery duration what was random? The later explanation in the article is correct, but this sentence is not.

6. “The flow diagram of participant enrollment and allocation in figure 1.”

Incorrect sentence, please correct it.

Best regards

**********

7. PLOS authors have the option to publish the peer review history of their article (what does this mean?). If published, this will include your full peer review and any attached files.

If you choose “no”, your identity will remain anonymous but your review may still be made public.

**Do you want your identity to be public for this peer review?**  For information about this choice, including consent withdrawal, please see our Privacy Policy.

Reviewer #1: No

Reviewer #2: **Yes:** Madhu Kalyan Pendurthi

Reviewer #3: **Yes:** Dapanaduwage Amila Vindana Rathnapala

Reviewer #4: No

**********

[NOTE: If reviewer comments were submitted as an attachment file, they will be attached to this email and accessible via the submission site. Please log into your account, locate the manuscript record, and check for the action link "View Attachments". If this link does not appear, there are no attachment files.]

To ensure your figures meet our technical requirements, please review our figure guidelines: https://journals.plos.org/plosone/s/figures

You may also use PLOS’s free figure tool, NAAS, to help you prepare publication quality figures: https://journals.plos.org/plosone/s/figures#loc-tools-for-figure-preparation.

NAAS will assess whether your figures meet our technical requirements by comparing each figure against our figure specifications.

# Author comment: Author response to Decision Letter 3

**DOI:** 10.1371/journal.pone.0351553.r006

24 Apr 2026

Reviewer #1:

1. Table 3 remove the t-value from the table.

Reply: Thank you for the reviewer’s comment. The t-values have been removed from Table 3. (line367)

2. Table 4; Present only the beta and a 95% CI. Remove Se, Wald chi square, df and p value.

Reply: Thank you for the reviewer’s suggestion. We have revised Table 4 to present only the β coefficients and 95% confidence intervals, and have removed the SE, Wald chi-square, degrees of freedom, and p-values accordingly. (line 414)

3. table 4 is described as interaction effects between group and time. Are you sure that is what you are presenting here? Please write out the model and show which parameters are included in the table.

Reply: Thank you for the reviewer’s comment. We confirm that the results presented in Table 4 represent the interaction effects (group × time) in the GEE analysis.

In our study, the GEE model included group, time, and the group × time interaction term as the main independent variables. The β coefficients presented in Table 4 correspond to the interaction terms, reflecting the differences in the magnitude of change before and after the intervention between each EG and the CG. A clarification has also been added below Table 4. (line 416-419)

4. How were the subjects who dropped out handled? If these are excluded then this is not an intention to treat analysis and this should be acknowledged as a potential source of bias.

Reply: Thank you for the reviewer’s comment. In this study, participants who dropped out were excluded from the final analysis; therefore, a per-protocol analysis was conducted rather than an intention-to-treat (ITT) analysis. We acknowledge that this approach may introduce attrition bias, particularly if the characteristics of participants who dropped out differ from those who completed the study, which may affect the internal validity of the results. This potential limitation has been addressed in both the Methods and Limitations sections to provide a more comprehensive explanation. (line 142 and line 564-566)

5. Please also acknowledge as a limitation the very small size of the control group, which was not stratified by length of long COVID.

Reply: Thank you for the reviewer’s comment. This study adopted a quasi-experimental design, with the primary aim of comparing intervention effects across different recovery durations; therefore, stratification based on recovery time was applied only within the EGs. The CG was used as a reference for the natural progression of symptoms without intervention and was not further stratified. We agree that the small sample size of the CG and the lack of stratification by duration of long COVID may affect the stability of between-group comparisons and the internal validity of the study, thereby influencing the interpretation of the results. This limitation has been acknowledged in the revised manuscript. (line 560-564)

Reviewer #2: (No Response)

Reviewer #3: The authors have thoroughly and satisfactorily addressed my previous concerns regarding the study design and the intervention's clinical role.

Specifically, the authors provided crucial context regarding the lack of standardized pulmonary rehabilitation programs in the study's region during the data collection period. This adequately justifies the composition of the standard care control arm and resolves my ethical and methodological concerns. Furthermore, the revised Introduction accurately positions Incentive Spirometry not as a replacement for comprehensive pulmonary rehabilitation, but as a practical, low-cost, and accessible adjunct for resource-limited settings.

These clarifications significantly strengthen the manuscript's clinical relevance and scientific integrity. I have no further concerns and recommend the manuscript for publication.

Reply: Thank you for the reviewer’s comments. We will continue to refine our work and contribute further evidence in this field in future studies.

Reviewer #4: Dear colleagues, thank you for giving me the opportunity to review your article. Basically, the article is of interest in the sense of post-covid rehabilitation possibilities. I had not the opportunity to review previous versions, but this version is much improved. I would have some minor suggestions to even more improve your article:

1. “Study on Post-Acute COVID-19 Syndrome in Improvement of 2 COVID-19 Rehabilitated Patients by Respiratory Training.”

The article title can be misleading and not informative enough what the article is about so I would suggest you to rephrase it.

Reply: Thank you for the reviewer’s suggestion. We agree that the original title was not sufficiently precise and did not fully reflect the focus of the study. Therefore, we have revised the title to “Effects of Incentive Spirometer Training on Dyspnea and Functional Status in Patients with Long COVID,” emphasizing the main intervention and key outcome measures (dyspnea and functional status) to improve clarity and informativeness.

2. “Currently, no definitive treatment for Long COVID.”

This is not a valid sentence, correct it.

Reply: Thank you for the reviewer’s suggestion. We have corrected the grammatical error and revised the sentence to avoid an overly absolute statement. (line 86)

3. “Respiratory training—commonly used to manage symptoms in chronic pulmonary disease—is considered a potentially beneficial intervention for Long COVID; however, supporting evidence remains limited.”

This is not truth, evidence exist so please rephrase this sentence and add new references. That should be corrected throughout the article.

Reply: Thank you for the reviewer’s comment. We agree that existing studies have supported the potential benefits of respiratory training for long COVID. Therefore, we have revised the statement to emphasize the heterogeneity of the current evidence and the lack of consistent conclusions, and have added relevant references to provide a more comprehensive and accurate description. (line87-91)

4. “provides patients with an alternative choice”

No truth because it was not compared with pulmonary rehabilitation. You should omit this sentence.

Reply: Thank you for the reviewer’s suggestion. We agree with this concern and have removed the statement accordingly. (line 111)

5. “Participants were randomly assigned to groups based on recovery duration.”

If they were assigned by recovery duration what was random? The later explanation in the article is correct, but this sentence is not.

Reply: Thank you for the reviewer’s comment. We agree that the original statement was not sufficiently precise and may have caused misunderstanding regarding the group allocation process. In this study, participants were first randomly assigned to the experimental and control groups, and the experimental group was subsequently stratified based on recovery duration. Therefore, the grouping based on recovery duration was not part of the randomization process. The relevant sentence has been revised to more clearly describe the study design. (line120 and line 258-260)

6. “The flow diagram of participant enrollment and allocation in figure 1.”

Incorrect sentence, please correct it.

Reply: Thank you for the reviewer’s comment. We have revised the sentence for clarity and accuracy. (line 126)

Attachment Submitted filename: Response to Reviewers 0420.docx

# Peer review: Decision Letter 3

**DOI:** 10.1371/journal.pone.0351553.r007

**Authors:** Toufic Ahmad Chaaban

18 May 2026

PONE-D-25-41209R3Effects of Incentive Spirometer Training on Dyspnea and Functional Status in Patients with Long COVIDPLOS One

Dear Dr. Hsieh,

Thank you for submitting your manuscript to PLOS ONE. After careful consideration, we feel that it has merit but does not fully meet PLOS ONE’s publication criteria as it currently stands. Therefore, we invite you to submit a revised version of the manuscript that addresses the points raised during the review process.

==============================

**ACADEMIC EDITOR:** Dear Authors, please make suggested revisions according to reviewers' comments or write a detailed rebuttal on a point-by-point basis.

==============================

Please submit your revised manuscript by Jul 02 2026 11:59PM. If you will need more time than this to complete your revisions, please reply to this message or contact the journal office at plosone@plos.org. When you're ready to submit your revision, log on to https://www.editorialmanager.com/pone/ and select the 'Submissions Needing Revision' folder to locate your manuscript file.

Please include the following items when submitting your revised manuscript:

- A letter that responds to each point raised by the academic editor and reviewer(s). You should upload this letter as a separate file labeled 'Response to Reviewers'.
- A marked-up copy of your manuscript that highlights changes made to the original version. You should upload this as a separate file labeled 'Revised Manuscript with Track Changes'.
- An unmarked version of your revised paper without tracked changes. You should upload this as a separate file labeled 'Manuscript'.

If you would like to make changes to your financial disclosure, please include your updated statement in your cover letter. Guidelines for resubmitting your figure files are available below the reviewer comments at the end of this letter.

If applicable, we recommend that you deposit your laboratory protocols in protocols.io to enhance the reproducibility of your results. Protocols.io assigns your protocol its own identifier (DOI) so that it can be cited independently in the future. For instructions see: https://journals.plos.org/plosone/s/submission-guidelines#loc-laboratory-protocols. Additionally, PLOS ONE offers an option for publishing peer-reviewed Lab Protocol articles, which describe protocols hosted on protocols.io. Read more information on sharing protocols at https://plos.org/protocols?utm_medium=editorial-email&utm_source=authorletters&utm_campaign=protocols.

As the corresponding author, your ORCID iD is verified in the submission system and will appear in the published article. PLOS supports the use of ORCID, and we encourage all coauthors to register for an ORCID iD and use it as well. Please encourage your coauthors to verify their ORCID iD within the submission system before final acceptance, as unverified ORCID iDs will not appear in the published article. *Only*  the individual author can complete the verification step; PLOS staff *cannot*  verify ORCID iDs on behalf of authors.

We look forward to receiving your revised manuscript.

Kind regards,

Davor Plavec, MD, MSc, PhD, Prof.

Academic Editor

PLOS One

**Journal Requirements:**

If the reviewer comments include a recommendation to cite specific previously published works, please review and evaluate these publications to determine whether they are relevant and should be cited. There is no requirement to cite these works unless the editor has indicated otherwise.

**Additional Editor Comments:**

Dear Authors,

please make suggested revisions according to reviewers' comments or write a detailed rebuttal on a point-by-point basis.

[Note: HTML markup is below. Please do not edit.]

Reviewers' comments:

Reviewer's Responses to Questions

**Comments to the Author**

1. If the authors have adequately addressed your comments raised in a previous round of review and you feel that this manuscript is now acceptable for publication, you may indicate that here to bypass the “Comments to the Author” section, enter your conflict of interest statement in the “Confidential to Editor” section, and submit your "Accept" recommendation.

Reviewer #1: (No Response)

Reviewer #3: All comments have been addressed

Reviewer #4: (No Response)

**********

2. Is the manuscript technically sound, and do the data support the conclusions?

The manuscript must describe a technically sound piece of scientific research with data that supports the conclusions. Experiments must have been conducted rigorously, with appropriate controls, replication, and sample sizes. The conclusions must be drawn appropriately based on the data presented.

Reviewer #1: Yes

Reviewer #3: Partly

Reviewer #4: No

**********

3. Has the statistical analysis been performed appropriately and rigorously?

Reviewer #1: I Don't Know

Reviewer #3: N/A

Reviewer #4: I Don't Know

**********

4. Have the authors made all data underlying the findings in their manuscript fully available?

The PLOS Data policy requires authors to make all data underlying the findings described in their manuscript fully available without restriction, with rare exception (please refer to the Data Availability Statement in the manuscript PDF file). The data should be provided as part of the manuscript or its supporting information, or deposited to a public repository. For example, in addition to summary statistics, the data points behind means, medians and variance measures should be available. If there are restrictions on publicly sharing data—e.g. participant privacy or use of data from a third party—those must be specified.

Reviewer #1: No

Reviewer #3: Yes

Reviewer #4: Yes

**********

5. Is the manuscript presented in an intelligible fashion and written in standard English?

PLOS ONE does not copyedit accepted manuscripts, so the language in submitted articles must be clear, correct, and unambiguous. Any typographical or grammatical errors should be corrected at revision, so please note any specific errors here.

Reviewer #1: Yes

Reviewer #3: Yes

Reviewer #4: Yes

**********

6. Review Comments to the Author

Please use the space provided to explain your answers to the questions above. You may also include additional comments for the author, including concerns about dual publication, research ethics, or publication ethics. (Please upload your review as an attachment if it exceeds 20,000 characters)

Reviewer #1: Thank you for the revisions.

I think there is a problem with the values for the PCFS interaction terms presented in Table 4. These do not seem to line up with what would be expected given the results presented in Table3. Please verify.

The other values presented are close to what is presented in Table 3.

Reviewer #3: The authors have successfully maintained the crucial clarifications requested in the previous review rounds, effectively resolving my primary methodological and ethical concerns.

Specifically, the manuscript correctly contextualizes the control arm by acknowledging the absence of standardized pulmonary rehabilitation programs during the study period. This provides a solid ethical and scientific justification for the study design based on the clinical reality of the time.

Furthermore, the authors have appropriately defined the role of Incentive Spirometry. By framing it as a practical, self-administered adjunct rather than a replacement for comprehensive pulmonary rehabilitation, the study offers a highly relevant, low-cost option. This distinction is particularly valuable for optimizing respiratory care in resource-limited settings where formal, multidisciplinary rehabilitation programs may be inaccessible to patients.

The revisions have significantly strengthened the manuscript's clinical applicability and scientific integrity. The authors have adequately addressed all of my feedback, and I have no further concerns. I recommend this article for publication.

Reviewer #4: Dear colleagues,

Thank you for giving me the opportunity to review your article. This is an interesting idea but unfortunately, I don’t believe that you have scientifically proof your conclusion: “In conclusion, IS represents an effective and practical strategy for alleviating dyspnea and improving functional limitations in individuals with Long COVID.“

The design of your study has many limitations as you have correctly stated in the limitations section but there is also one big limitation and you have not mentioned it. The lung function analysis of the patients with CO diffusion capacity which is known to be the important parameter considering the exercise limitation in post-COVID patients. From my point of view, no studies of post-COVID respiratory investigations should be done or at least published without the DLco data. Also, the intervention itself could have important placebo effect that was not possible to exclude by the study design.

If you can correct for previously stated problems, I would be glad to re-review your article.

Best regards

**********

7. PLOS authors have the option to publish the peer review history of their article (what does this mean?). If published, this will include your full peer review and any attached files.

If you choose “no”, your identity will remain anonymous but your review may still be made public.

**Do you want your identity to be public for this peer review?**  For information about this choice, including consent withdrawal, please see our Privacy Policy.

Reviewer #1: No

Reviewer #3: **Yes:** Dapanaduwage Amila Vindana Rathnapala

Reviewer #4: No

**********

[NOTE: If reviewer comments were submitted as an attachment file, they will be attached to this email and accessible via the submission site. Please log into your account, locate the manuscript record, and check for the action link "View Attachments". If this link does not appear, there are no attachment files.]

To ensure your figures meet our technical requirements, please review our figure guidelines: https://journals.plos.org/plosone/s/figures

You may also use PLOS’s free figure tool, NAAS, to help you prepare publication quality figures: https://journals.plos.org/plosone/s/figures#loc-tools-for-figure-preparation.

NAAS will assess whether your figures meet our technical requirements by comparing each figure against our figure specifications.

# Author comment: Author response to Decision Letter 4

**DOI:** 10.1371/journal.pone.0351553.r008

23 May 2026

Reviewer #1: Thank you for the revisions.

I think there is a problem with the values for the PCFS interaction terms presented in Table 4. These do not seem to line up with what would be expected given the results presented in Table3. Please verify.

The other values presented are close to what is presented in Table 3.

Reply: Thank you for your careful review and for identifying this issue. After re-examining the analysis, we found that the previous GEE model for the PCFS scale had been conducted using an ordinal-variable approach, whereas Table 3 presented the PCFS results as continuous mean score changes. This difference in modeling strategy may have contributed to inconsistency in the interpretation of the interaction coefficients presented in Table 4.

To improve the consistency and interpretability of the results, we reanalyzed the PCFS outcomes using a continuous-variable GEE model and updated the corresponding interaction coefficients in Table 4 accordingly. We sincerely appreciate the reviewer’s careful observation, which helped us further improve the clarity of the manuscript. (P.19 Table4)

Reviewer #3: The authors have successfully maintained the crucial clarifications requested in the previous review rounds, effectively resolving my primary methodological and ethical concerns.

Specifically, the manuscript correctly contextualizes the control arm by acknowledging the absence of standardized pulmonary rehabilitation programs during the study period. This provides a solid ethical and scientific justification for the study design based on the clinical reality of the time.

Furthermore, the authors have appropriately defined the role of Incentive Spirometry. By framing it as a practical, self-administered adjunct rather than a replacement for comprehensive pulmonary rehabilitation, the study offers a highly relevant, low-cost option. This distinction is particularly valuable for optimizing respiratory care in resource-limited settings where formal, multidisciplinary rehabilitation programs may be inaccessible to patients.

The revisions have significantly strengthened the manuscript's clinical applicability and scientific integrity. The authors have adequately addressed all of my feedback, and I have no further concerns. I recommend this article for publication.

Reply: We sincerely thank the reviewer for the thoughtful comments and positive evaluation of our manuscript. Your feedback has been highly valuable in strengthening the clinical applicability and scientific rigor of this study. We are grateful for your encouragement and will continue striving to improve the quality and clinical relevance of our future research.

Reviewer #4: Dear colleagues,

Thank you for giving me the opportunity to review your article. This is an interesting idea but unfortunately, I don’t believe that you have scientifically proof your conclusion: „In conclusion, IS represents an effective and practical strategy for alleviating dyspnea and improving functional limitations in individuals with Long COVID.“

The design of your study has many limitations as you have correctly stated in the limitations section but there is also one big limitation and you have not mentioned it. The lung function analysis of the patients with CO diffusion capacity which is known to be the important parameter considering the exercise limitation in post-COVID patients. From my point of view, no studies of post-COVID respiratory investigations should be done or at least published without the DLco data. Also, the intervention itself could have important placebo effect that was not possible to exclude by the study design.

If you can correct for previously stated problems, I would be glad to re-review your article.

Best regards

Reply: We sincerely thank the reviewer for the thoughtful and constructive comments. We agree that the conclusions of our study should be interpreted cautiously given the limitations of the study design. Accordingly, we have revised the limitation and conclusions throughout the manuscript to avoid overly definitive statements regarding the effectiveness of IS intervention.

Regarding the absence of DLco assessment, we fully acknowledge that diffusion capacity is an important parameter when evaluating pulmonary impairment and exercise limitation in patients with post-COVID conditions. However, the primary aim of this study was to evaluate the feasibility and potential benefits of a simple, low-cost, home-based respiratory training intervention that could be implemented in non-hospitalized individuals recovering from COVID-19.

During the study period (2023–2024), most participants had already returned to community or home settings and were not hospitalized. Performing DLco measurements would have required participants to repeatedly return to the hospital pulmonary function laboratory, which posed practical difficulties and potentially increased unnecessary exposure risks during the post-pandemic period.

In addition, at the time the study protocol was initially designed, there was still limited evidence regarding standardized respiratory assessment strategies for Long COVID. Therefore, DLco measurement was not incorporated into the original study design. We appreciate the reviewer for highlighting this important issue, and we agree that future studies should incorporate comprehensive pulmonary function assessments, including DLco analysis, to further clarify the physiological mechanisms underlying symptom improvement. (P.25 line580-585)

We also agree that placebo effects and expectation-related responses could not be fully excluded because of the open-label design and the absence of a sham intervention group. Therefore, we have further emphasized this issue in the Limitations section and revised the interpretation of the findings accordingly. (P.26 line 577-579)

Attachment Submitted filename: Response to Reviewer 0520.pdf

# Peer review: Decision Letter 4

**DOI:** 10.1371/journal.pone.0351553.r009

**Authors:** Toufic Ahmad Chaaban

28 May 2026

Effects of Incentive Spirometer Training on Dyspnea and Functional Status in Patients with Long COVID

PONE-D-25-41209R4

Dear Dr. Hsieh,

We’re pleased to inform you that your manuscript has been judged scientifically suitable for publication and will be formally accepted for publication once it meets all outstanding technical requirements.

Within one week, you’ll receive an e-mail detailing the required amendments. When these have been addressed, you’ll receive a formal acceptance letter and your manuscript will be scheduled for publication.

An invoice will be generated when your article is formally accepted. Please note, if your institution has a publishing partnership with PLOS and your article meets the relevant criteria, all or part of your publication costs will be covered. Please make sure your user information is up-to-date by logging into Editorial Manager at Editorial Manager® and clicking the ‘Update My Information' link at the top of the page. For questions related to billing, please contact billing support.

If your institution or institutions have a press office, please notify them about your upcoming paper to help maximize its impact. If they’ll be preparing press materials, please inform our press team as soon as possible -- no later than 48 hours after receiving the formal acceptance. Your manuscript will remain under strict press embargo until 2 pm Eastern Time on the date of publication. For more information, please contact onepress@plos.org.

Kind regards,

Davor Plavec, MD, MSc, PhD, Prof.

Academic Editor

PLOS One

Additional Editor Comments (optional):

Reviewers' comments:

Reviewer's Responses to Questions

**Comments to the Author**

1. If the authors have adequately addressed your comments raised in a previous round of review and you feel that this manuscript is now acceptable for publication, you may indicate that here to bypass the “Comments to the Author” section, enter your conflict of interest statement in the “Confidential to Editor” section, and submit your "Accept" recommendation.

Reviewer #1: All comments have been addressed

**********

2. Is the manuscript technically sound, and do the data support the conclusions?

The manuscript must describe a technically sound piece of scientific research with data that supports the conclusions. Experiments must have been conducted rigorously, with appropriate controls, replication, and sample sizes. The conclusions must be drawn appropriately based on the data presented.

Reviewer #1: Yes

**********

3. Has the statistical analysis been performed appropriately and rigorously?

Reviewer #1: Yes

**********

4. Have the authors made all data underlying the findings in their manuscript fully available?

The PLOS Data policy requires authors to make all data underlying the findings described in their manuscript fully available without restriction, with rare exception (please refer to the Data Availability Statement in the manuscript PDF file). The data should be provided as part of the manuscript or its supporting information, or deposited to a public repository. For example, in addition to summary statistics, the data points behind means, medians and variance measures should be available. If there are restrictions on publicly sharing data—e.g. participant privacy or use of data from a third party—those must be specified.

Reviewer #1: Yes

**********

5. Is the manuscript presented in an intelligible fashion and written in standard English?

PLOS ONE does not copyedit accepted manuscripts, so the language in submitted articles must be clear, correct, and unambiguous. Any typographical or grammatical errors should be corrected at revision, so please note any specific errors here.

Reviewer #1: Yes

**********

6. Review Comments to the Author

Please use the space provided to explain your answers to the questions above. You may also include additional comments for the author, including concerns about dual publication, research ethics, or publication ethics. (Please upload your review as an attachment if it exceeds 20,000 characters)

Reviewer #1: (No Response)

**********

7. PLOS authors have the option to publish the peer review history of their article (what does this mean?). If published, this will include your full peer review and any attached files.

If you choose “no”, your identity will remain anonymous but your review may still be made public.

**Do you want your identity to be public for this peer review?**  For information about this choice, including consent withdrawal, please see our Privacy Policy.

Reviewer #1: No

**********

# Editor report: Acceptance letter

**DOI:** 10.1371/journal.pone.0351553.r010

**Authors:** Toufic Ahmad Chaaban

PONE-D-25-41209R4

PLOS One

Dear Dr. Hsieh,

I'm pleased to inform you that your manuscript has been deemed suitable for publication in PLOS One. Congratulations! Your manuscript is now being handed over to our production team.

At this stage, our production department will prepare your paper for publication. This includes ensuring the following:

* All references, tables, and figures are properly cited

* All relevant supporting information is included in the manuscript submission,

* There are no issues that prevent the paper from being properly typeset

You will receive further instructions from the production team, including instructions on how to review your proof when it is ready. Please keep in mind that we are working through a large volume of accepted articles, so please give us a few days to review your paper and let you know the next and final steps.

Lastly, if your institution or institutions have a press office, please let them know about your upcoming paper now to help maximize its impact. If they'll be preparing press materials, please inform our press team within the next 48 hours. Your manuscript will remain under strict press embargo until 2 pm Eastern Time on the date of publication. For more information, please contact onepress@plos.org.

You will receive an invoice from PLOS for your publication fee after your manuscript has reached the completed accept phase. If you receive an email requesting payment before acceptance or for any other service, this may be a phishing scheme. Learn how to identify phishing emails and protect your accounts at https://explore.plos.org/phishing.

If we can help with anything else, please email us at customercare@plos.org.

Thank you for submitting your work to PLOS ONE and supporting open access.

Kind regards,

PLOS ONE Editorial Office Staff

on behalf of

Dr. Davor Plavec

Academic Editor

PLOS One
